# Triglyceride-Rich Lipoproteins Modulate the Distribution and Extravasation of Ly6C/Gr1^low^ Monocytes

**DOI:** 10.1016/j.celrep.2015.08.020

**Published:** 2015-09-03

**Authors:** Maha F. Saja, Lucie Baudino, William D. Jackson, H. Terence Cook, Talat H. Malik, Liliane Fossati-Jimack, Marieta Ruseva, Matthew C. Pickering, Kevin J. Woollard, Marina Botto

**Affiliations:** 1 Centre for Complement and Inflammation Research, Division of Immunology and Inflammation, Department of Medicine, Imperial College London, Du Cane Road, London W12 ONN, UK; 2 Renal and Vascular Inflammation Section, Division of Immunology and Inflammation, Department of Medicine, Imperial College London, Du Cane Road, London W12 ONN, UK

## Abstract

Monocytes are heterogeneous effector cells involved in the maintenance and restoration of tissue integrity. However, their response to hyperlipidemia remains poorly understood. Here, we report that in the presence of elevated levels of triglyceride-rich lipoproteins, induced by administration of poloxamer 407, the blood numbers of non-classical Ly6C/Gr1^low^ monocytes drop, while the number of bone marrow progenitors remains similar. We observed an increased crawling and retention of the Gr1^low^ monocytes at the endothelial interface and a marked accumulation of CD68^+^ macrophages in several organs. Hypertriglyceridemia was accompanied by an increased expression of tissue, and plasma CCL4 and blood Gr1^low^ monocyte depletion involved a pertussis-toxin-sensitive receptor axis. Collectively, these findings demonstrate that a triglyceride-rich environment can alter blood monocyte distribution, promoting the extravasation of Gr1^low^ cells. The behavior of these cells in response to dyslipidemia highlights the significant impact that high levels of triglyceride-rich lipoproteins may have on innate immune cells.

## Highlights

• Hypertriglyceridemia promotes the extravasation of Gr1^low^ monocytes
• CD68^+^ tissue macrophages accumulate in the absence of overt inflammation
• Hypertriglyceridemia is associated with increased CCL4 levels
• CCL4 is involved in the migration of Gr1^low^ monocytes

## Graphical Abstract

**Figure:**
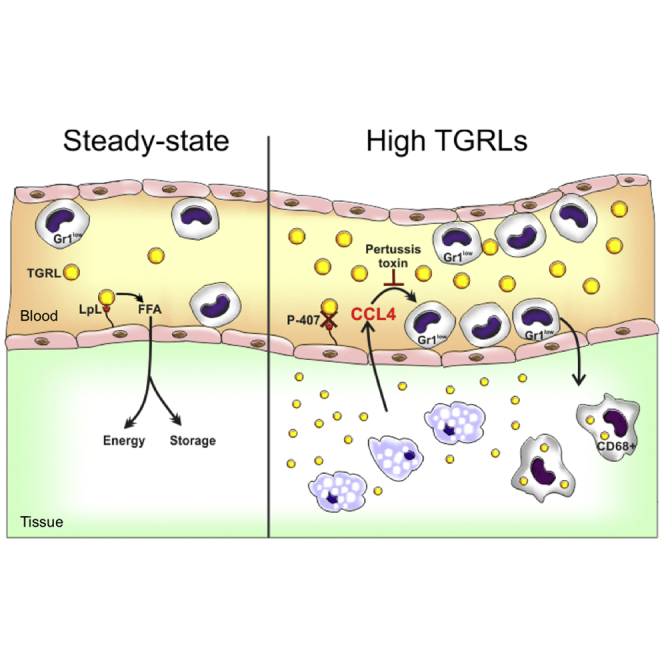

## Teaser

High levels of triglyceride-rich lipoproteins have been implicated in the pathogenesis of cardiovascular complications. Saja et al. demonstrate that hypertriglyceridemia promotes extravasation of Gr1^low^ monocytes and accumulation of tissue macrophages. Hypertriglyceridemia was associated with increased levels of CCL4, which contributes to the migration of Gr1^low^ monocytes.

## Introduction

Marked elevations in triglyceride-rich lipoprotein (TGRL) levels are observed in individuals with rare genetic disorders such as familial lipoprotein lipase deficiency (Benlian et al., 1996) and when a common genetic disorder occurs in association with an acquired secondary form of hypertriglyceridemia such as diabetes or alcohol consumption (Pejic and Lee, 2006). Furthermore, abnormalities in TGRL levels are commonly observed in patients with persistent infections like HIV (Oh and Hegele, 2007) or chronic inflammatory conditions such as systemic lupus erythematosus (SLE) (Bruce, 2005). Although elevated TGRL levels are thought to contribute to the increased risk of cardiovascular complications observed in all these conditions (Benlian et al., 1996; Bruce, 2005), the pathogenic impact of an abnormal TGRL profile remains poorly understood.

The mononuclear phagocyte system (MPS) plays a central role in the maintenance of tissue integrity. In a hyperlipidemic environment, cells of the MPS ingest excess lipids that activate them through a variety of signaling pathways, leading to increased secretion of pro-inflammatory cytokines and eventually cell death (Moore and Tabas, 2011). The notion of toxic and inflammatory effects of lipid metabolites has been supported by an extensive literature using murine models of hyperlipidemia such as strains genetically deficient in either the low-density lipoprotein receptor (LDLR) or apolipoprotein E (ApoE). These models replicate human hypercholesterolemic states and the associated inflammatory response, but they do not recapitulate triglyceride-rich dyslipidemia. Moreover, the nature of the lipid responsible for the MPS responses in hyperlipidemia remains unresolved; both inflammatory and anti-inflammatory effects have been attributed to cholesterol (Spann and Glass, 2013). Finally, the MPS cell type responding to hyperlipidemia remains poorly understood. Most work has focused on dendritic cells and tissue macrophages, while much less attention has been given to blood monocytes. However, reports of postprandial activation of monocytes from acute changes in TGRLs (Gower et al., 2011) suggest that these cells may be important in the handling of circulating lipids.

Monocytes, identified as CD11b^+^CD115^+^ cells, are a heterogeneous population of blood leukocytes with phagocytic and immunomodulatory properties. At least two phenotypically and functionally distinct monocyte subsets have been described in humans, rats, pigs, and mice (Geissmann et al., 2003; Yona and Jung, 2010; Ziegler-Heitbrock, 2014), indicating evolutionary conservation. The murine monocyte subpopulations have been termed “classical” and “non-classical” based on differences in surface markers and functional properties. The classical monocytes express lower levels of CX3CR1 and higher levels of the C-C chemokine receptor 2 (CCR2) and lymphocyte antigen 6c (Ly6C) (or the myeloid differentiation antigen Gr1) and are defined as CX3CR1^int^CCR2^high^Gr1^high^ (abbreviated as Gr1^high^). They are considered to be equivalent to CD14^high^ human monocytes (Cros et al., 2010). The Gr1^high^ cells represent the inflammatory monocyte subtype and are actively recruited to inflamed tissue, where they may give rise to macrophages. Their behavior appears to be distinct from the second blood monocyte subpopulation, identified as CX3CR1^high^CCR2^low^Gr1^low^ (abbreviated as Gr1^low^). The equivalent cells in humans are identified as CD14^low^CD16^high^ (Cros et al., 2010). The Gr1^low^ monocytes have been shown to patrol the luminal surface of endothelial cells, acting as “housekeepers” of the vasculature (Auffray et al., 2007; Carlin et al., 2013b). Their migratory properties remain elusive, and the possibility they may sense and respond to different environmental stimuli in the absence of inflammation is unexplored.

Given the fact that in steady-state conditions, monocytes do not contribute to the maintenance of most peripheral tissue macrophages (Hashimoto et al., 2013; Yona et al., 2013) but are exposed to changes in plasma lipids, their behavior during hyperlipidemia requires investigation. Here, we report that a hyper-TGRL environment promotes differential migration of blood monocytes. Hyper-TGRL induced Gr1^low^ monocytes to extravasate into surrounding tissue. This process was associated with increased CCL4 levels and was partially dependent on a pertussis toxin (PT)-sensitive receptor axis. Our findings indicate that Gr1^low^ monocytes, in addition to patrolling the endothelial luminal surface (Auffray et al., 2007; Carlin et al., 2013b), are likely to have an extravascular role under hyper-TGRL conditions.

## Results

### Elevated TGRL Levels Alter Blood Monocyte Subset Distribution

To mimic the increased TGRL levels observed in many patients with metabolic disorders, we used the poloxamer 407 (P-407)-induced model of dyslipidemia (Johnston, 2004). This chemically induced model of hyperlipidemia involves the intraperitoneal (i.p.) administration of the non-ionic surfactant P-407 to wild-type C57BL/6 (B6) mice on chow diet and allows for a dose-controlled dyslipidemia. We first established the dosing regimen that resulted in a stable increase in TGRL levels. As previously reported (Johnston, 2004), we found that administration of P-407 (0.5 g/kg every second day) to B6 mice for 28 days resulted in an average plasma triglyceride (TG) concentration of 2,691.2 ± 501.8 mg/dl compared with 80.2 ± 6.26 mg/dl in PBS-treated controls and a modest increase in plasma cholesterol (CHOL) (324 ± 37.1 mg/dl in P-407-treated mice versus 106.2 ± 3.27 mg/dl in PBS-treated mice [mean ± SEM]) (Figures S1A–S1D). Chromatography showed that in P-407-treated animals, all of the TG was associated with the VLDL fraction (Figures S1E and S1F), consisting mainly of Apo-E and apolipoprotein C-III (APOC3) (Figure S1G). Importantly, the P-407-induced dyslipidemia did not trigger an overt inflammatory response (Figure S1H). The body weight of the P-407-injected animals did not change (Figure S1I) and no evidence of renal damage was detected as judged by the absence of hematuria and albuminuria (data not shown). As previously reported (Johnston et al., 1993), there was a mild splenomegaly in the P-407-treated group when compared to controls (Figure S1J). Histological analysis after 4-week treatment showed some cells with a characteristic foamy cytoplasm in the spleen, liver, and heart (Figure S1K), indicating increased tissue lipid load and foam cell formation.

**Figure 1:**
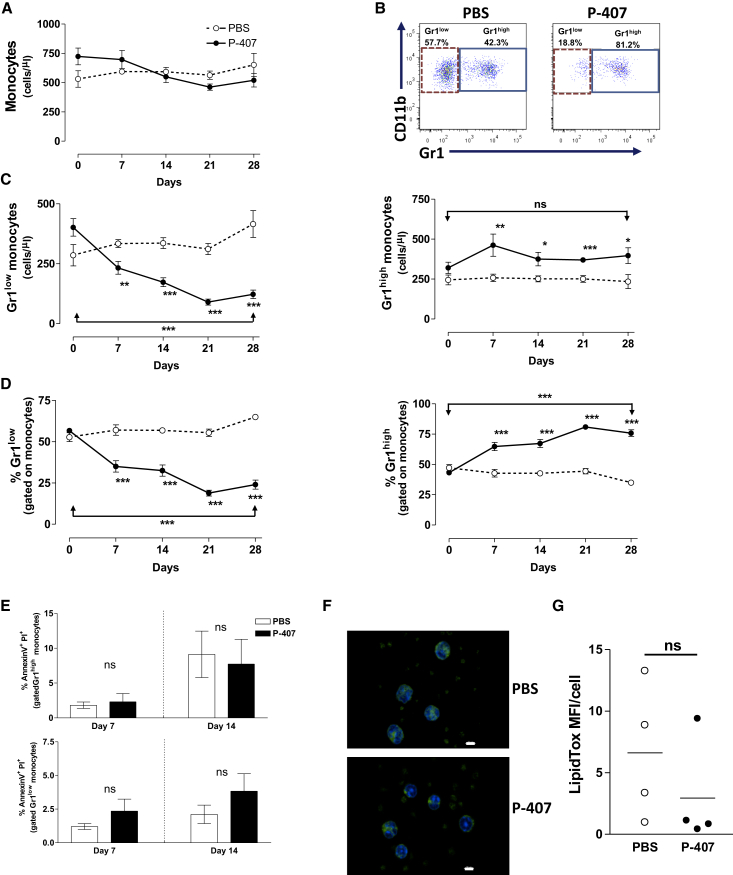
Characterization of Blood Monocytes in P-407-Treated Mice (A) Quantitative analysis of total monocyte numbers in B6 mice treated with P-407 or PBS for 28 days. Circulating monocytes were identified by their low SSC and co-expression of CD115 and CD11b. (B) Representative dot plots showing the frequencies of Gr1^high^ and Gr1^low^ monocyte subsets at the end of the time course. (C and D) Numbers (C) and relative frequencies (D) of Gr1^low^ and Gr1^high^ blood monocytes. P-407-treated mice kept on a chow diet had significantly fewer Gr1^low^ and more Gr1^high^ monocytes at each time point. Plots represent data pooled from four different experiments and show the mean values ± SE for at least seven mice per time point. The p value of the comparison between day 0 and day 28 is indicated (^∗^p < 0.05; ^∗∗^p < 0.01, and ^∗∗∗^p < 0.001; unpaired t test). (E) Frequency of Annexin V^+^/PI^+^ monocytes in the two monocyte subpopulations 7 and 14 days after P-407 treatment. Results are expressed as mean ± SE, n = 4 per time point (unpaired t test). (F) Representative images of neutral lipid content in blood leukocytes after 28 days of PBS or P-407 treatment. Leukocytes were stained for neutral lipid accumulation using the LipidTox kit according to manufacturer’s instructions. Neutral lipid droplets are shown in green. Scale bar, 10 μm. (G) Quantification of the lipid content. Data are expressed as mean fluorescent intensity (MFI) per cell (mean of three fields per mouse, 15 cells/field, and four mice per group; unpaired t test). Horizontal bars represent means. ns, nonsignificant. See also Figures S1 and S2.

Diet-induced hypercholesterolemia in ApoE-deficient mice has been shown to induce monocytosis associated mainly with a shift toward an increased frequency of the Gr1^high^ subset compared with Gr1^low^ fraction (Swirski et al., 2007; Tacke et al., 2007). The increase in TGRLs induced by P-407 treatment did not alter the total number of blood monocytes (Figure 1A), but the distribution of the two major monocyte subsets showed a different pattern (Figure 1B). We observed a progressive and marked drop in the numbers of Gr1^low^ monocytes starting as early as 7 days post-P-407 injections with a relatively modest increase in the number of Gr1^high^ monocytes (Figures 1C and 1D). At the end point, the P-407-treated mice had dropped their Gr1^low^ monocyte numbers to less than half compared to the starting point (p < 0.0001), while the number of Gr1^high^ monocytes was not significantly different (p = 0.25). In contrast, other peripheral blood cells (total white cell, B and T cells, and polymorphonuclear leukocytes) did not show consistent or reproducible changes in their numbers or frequencies (Figures S2A–S2H). The drop in circulating Gr1^low^ monocytes under the P-407 treatment could not be explained by a selective toxic effect of the dyslipidemia as the percentages of the Annexin V^+^/PI^+^ monocytes were not markedly different between the two monocyte subpopulations, irrespective of the treatment (Figure 1E). Additionally, in vitro exposure of peripheral blood mononuclear cells (PBMCs) to P-407 concentrations ranging from 50 μg/ml to 10 mg/ml did not affect the survival of the cells (Figure S2I). We then investigated a model of diet-dependent hyperlipidemia using the LDLR-deficient (*Ldlr*^−/−^) mice that develop hypercholesterolemia and a modest hypertriglyceridemia on a high-fat (HF) diet (Figures S2J and S2K). Animals were euthanized after 50 days on the diet, an age at which *Ldlr*^−/−^ mice do not develop advanced atherosclerotic lesions (Ma et al., 2012) or monocytosis (Murphy et al., 2011) (Figure S2L). Under these conditions, we observed significantly fewer Gr1^low^ monocytes in the *Ldlr*^−/−^ mice kept on an HF diet compared to those maintained on a chow diet (Figures S2M and S2N), indicating that the changes in monocyte subsets in P-407-treated animals were the result of the dyslipidemia and not an adverse effect of P-407.

Consistent with the inhibitory effect of P-407 on the capillary endothelial lipoprotein lipase (Johnston, 2004), a known mediator of TG hydrolysis, we also found that P-407 did not induce accumulation of neutral lipid content in leukocytes (Figures 1F and 1G). On the contrary, there was a trend toward a decreased amount of lipid in the blood cells of the P-407-treated animals. Taken together these data indicate that the increase in TGRLs modulates the steady-state distribution of the blood monocyte subsets without altering other leukocytes. We then explored whether the changes triggered by the P-407 treatment occurred in the bone marrow (BM) or in the periphery.

### The P-407-Induced Dyslipidemic Environment Does Not Affect the Generation of Gr1^low^ Monocytes

**Figure 2:**
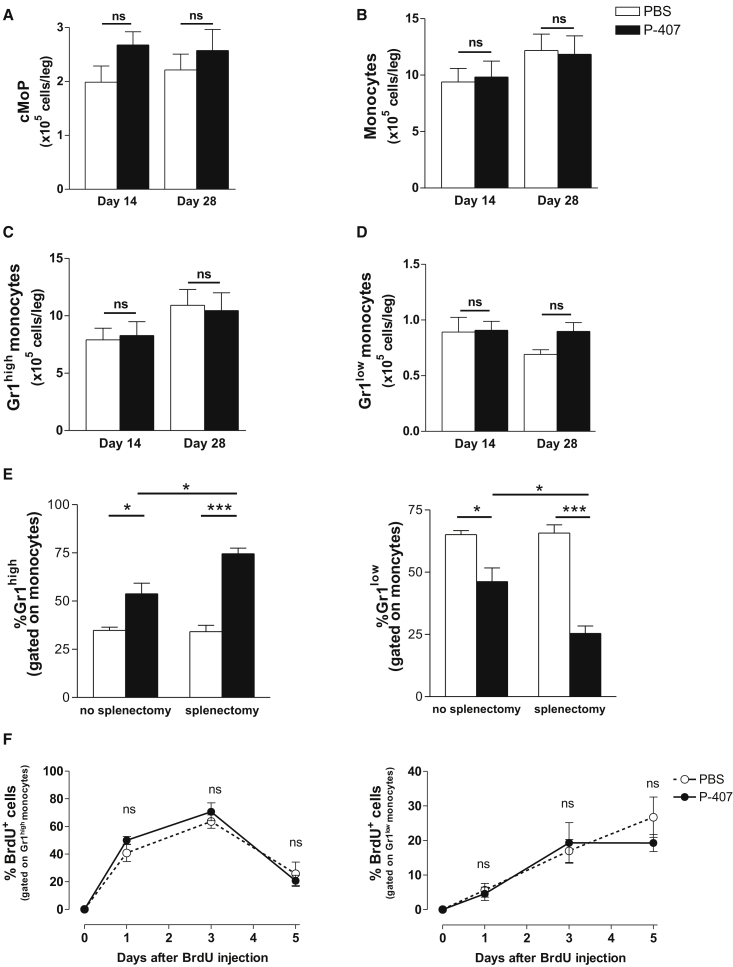
Modulation of BM and Blood Monocyte Dynamics in P-407-Treated Mice (A–D) Quantitative analysis of cMoPs (A), monocytes (B), and Gr1^high^ (C) and Gr1^low^ monocytes (D) in the BM after 14 and 28 days of P-407 or PBS treatment. Results are expressed as mean ± SE, n = 4 per time point. p values are indicated (unpaired t test); ns, nonsignificant. (E) Frequencies of Gr1^high^ and Gr1^low^ monocyte subsets after splenectomy in mice treated with PBS or P-407 for 14 days. Results are expressed as mean ± SE, n = 4. ^∗^p < 0.05 and ^∗∗∗^p < 0.001 (unpaired t test). (F) Percentage of BrdU incorporation into Gr1^high^ (left panel) and Gr1^low^ (right panel) monocytes over a period of 5 days following a single pulse of BrdU administered i.p. in three doses of 2 mg, 3 hr apart. Data are representative of two independent experiments. Results are expressed as mean ± SE, n = 3 in each group (unpaired t test); ns, nonsignificant. See also Figure S3.

We first examined the frequency of the BM-resident founder cells termed common monocyte progenitor (cMoP) (Hettinger et al., 2013). The analysis of the BM at 2 and 4 weeks failed to show any significant difference between the two experimental groups in the numbers of cMoPs, total monocytes, and monocyte subpopulations (Figures 2A–2D), demonstrating that the drop in Gr1^low^ monocytes could not be the result of an impaired production in the BM. As the spleen can contribute to the clearance of apoptotic monocytes (Getts et al., 2014) and at the same time act as a “reservoir” for the Gr1^high^ monocytes (Swirski et al., 2009), we assessed the effect of splenectomy on the monocyte distribution. Splenectomized mice treated with P-407 still had a drop in the percentage and number of Gr1^low^ monocytes when compared to the PBS-treated group (Figures 2E and S3A), confirming that P-407 did not promote the splenic clearance of apoptotic Gr1^low^ monocytes (see also Figure 1E).

Gr1^high^ monocytes have been shown to be the precursors of the steady-state Gr1^low^ cells (Tacke et al., 2006; Yona et al., 2013). Therefore, we used a 5-bromo-2′-deoxyuridine (BrdU) pulsing regimen to investigate whether the elevated TGRLs prevented Gr1^high^ > Gr1^low^ conversion or increased the half-life of Gr1^high^ blood monocytes. Mice were pulsed with three i.p. injections of 2 mg BrdU (3 hr apart), and treatment with P-407 or PBS was started on the same day. As shown in Figures 2F and S3B, the BrdU incorporation into the two monocyte subsets was tracked over a period of 5 days. By day 1, the vast majority of BrdU^+^ monocytes were Gr1^high^ (Figure 2F, left panel). This continued for 3 days when a significant drop of the BrdU^+^ monocytes was noted in both experimental groups, indicating that the dyslipidemic environment had not altered the half-life of the Gr1^high^ subset and thus this could not be the explanation for the drop in the Gr1^low^ compartment. A small fraction of the Gr1^low^ subset showed BrdU staining by day 1, and this gradually increased over 5 days in the PBS-treated group (Figure 2F, right panel), confirming the delayed BrdU incorporation into the Gr1^low^ compartment (Yona et al., 2013). The kinetic of the BrdU incorporation in the Gr1^low^ fraction differed slightly in the P-407-treated mice and the expected increase at day 5 was not observed (Figure 2F, right panel), suggesting the possibility of a different steady-state behavior of this monocyte compartment in the dyslipidemic environment. To investigate this, we studied effects of elevated TGRLs on the mobility of Gr1^low^ monocytes in vivo.

### Increased TGRL Levels Promote CX3CR1^high^ Monocyte Crawling and Tissue Accumulation of CD68^+^ Macrophages

**Figure 3:**
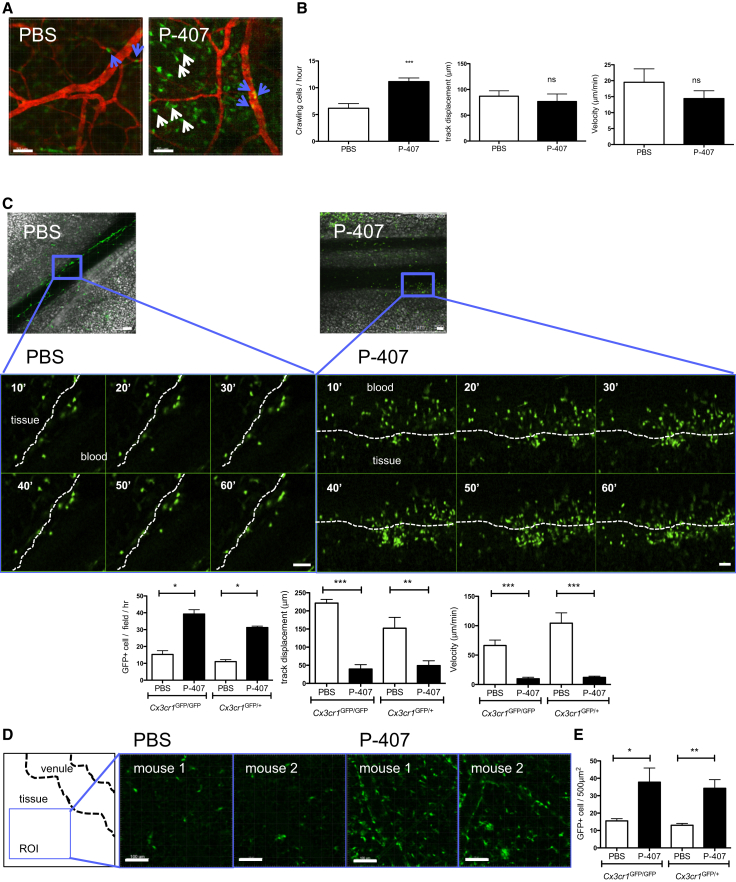
In Vivo Behavior of CX3CR1^high^ Monocytes under P-407-Induced Dyslipidemia *Cx3cr1*^gfp/+^ mice were treated with PBS or P-407 for 7 days and ear dermis vasculature imaged by intravital microscopy. (A) Examples of intravascular crawling cells (blue arrow) or tissue cells (white arrow) with PBS or P-407 treatment. Scale bars, 50 μm. (B) Quantitative representation of the number of intravascular crawling cells per hour, crawling velocity and track displacement after each treatment. n = 3 per group. ^∗∗∗^p < 0.001 from PBS control. ns, nonsignificant. Scale bars, 50 μm. (C) Same as (A) and (B) except that representative mesentery vasculature from *Cx3cr1*^gfp/gfp^ or *Cx3cr1*^gfp/+^ mice were imaged over 60 min. Region of interest (ROI) was selected which demonstrates gallery over time of CX3CR1^high^ cell accumulation at the endothelial interface (dotted line) following P-407 treatment. Representative of four mice; ^∗∗^p < 0.01. Scale bar, 50 μm. (D and E) As in (C), where (D) shows examples of tissue from an ROI (500 μm^2^) away from the venule showing accumulation of tissue GFP^+^ cells after P-407 treatment. Two mice representative of four. Scale bar, 100 μm. (E) Quantitative representation of the number of tissue GFP^+^ cells in PBS- and P-407-treated *Cx3cr1*^gfp/gfp^ or *Cx3cr1*^gfp/+^ mice. n = 3 per group. ^∗^p < 0.05 and ^∗∗^p < 0.01. See also Figure S4.

Gr1^low^ monocytes are thought to monitor endothelial integrity (Auffray et al., 2007; Carlin et al., 2013b). To examine the effect of the P-407-induced hyper-TGRL environment on the Gr1^low^ monocytes at the endothelial interface, we used the Cx3cr1^gfp^ reporter mouse strain. This has been widely used to discriminate the two monocyte subpopulations (Geissmann et al., 2003) and enables Gr1^low^CX3CR1^high^ monocytes to be tracked in situ by intravital microscopy (Auffray et al., 2007; Cros et al., 2010). Analysis of monocyte behavior in vivo showed that the number of intravascular crawling GFP^+^ cells per hour in the ear dermis was higher in the mice treated with P-407 for 7 days compared to the PBS counterparts (Figures 3A and 3B; Movies S1 and S2). Analysis of the intravital images revealed an increased accumulation of extravascular GFP^+^ cells with a migratory behavior in the P-407-treated animals (Figures 3A and S4A). We then performed in situ experiments in the mesentery of *Cx3cr1*^gfp/gfp^ and *Cx3cr1*^gfp/+^ mice (Figures 3C–3E) and found a marked accumulation of GFP^+^ cells at the endothelial interface over 1 hr in P-407-treated *Cx3cr1*^gfp/+^ and *Cx3cr1*^gfp/gfp^ mice (Figure 3C; Movies S3 and S4). This was accompanied by decreased velocity and track displacement (Figure 3C), both indicators of enhanced dwell time and endothelial retention. In addition, we observed again a significant increase in tissue (extravascular) GFP^+^ cells with a migratory behavior (Figures 3D, 3E, and S4B). Since there was no difference between *Cx3cr1*^gfp/+^ and *Cx3cr1*^gfp/gfp^ mice, this implies that under our hyper-TGRL conditions, the Gr1^low^ monocytes had acquired the ability to extravasate independently of CX3CL1 signaling.

**Figure 4:**
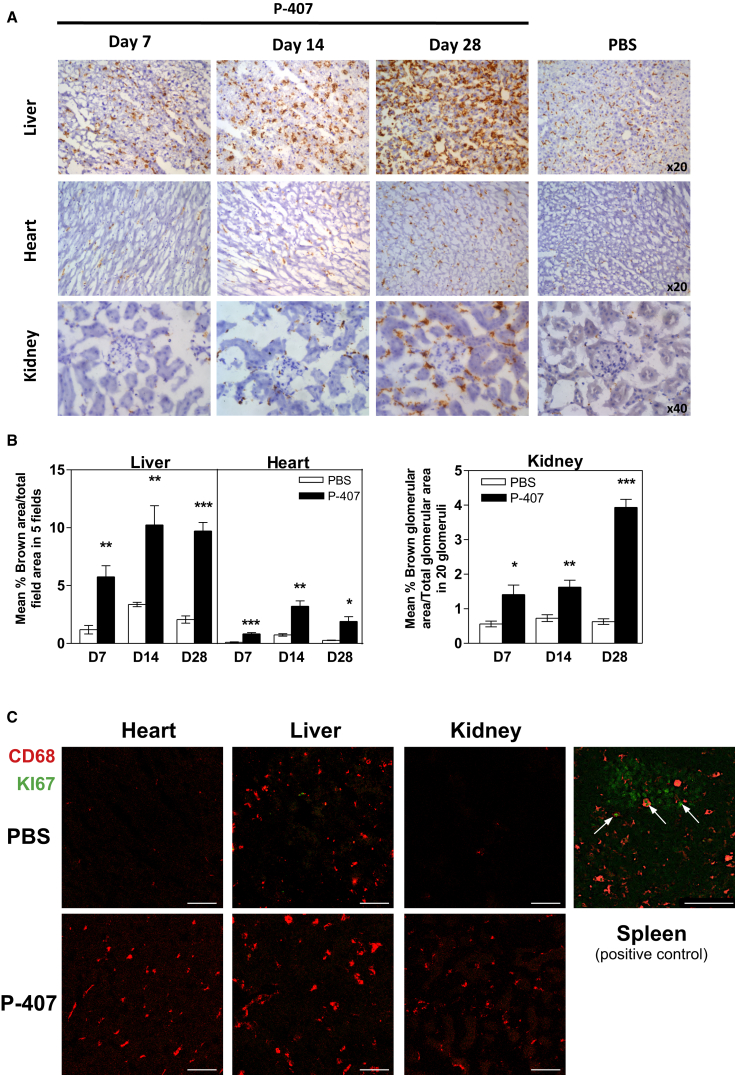
Tissue Accumulation of CD68^+^ Cells under P-407 Treatment (A) Representative photomicrographs of CD68 staining (brown) of PLP fixed liver, heart, and kidney sections after 7, 14, and 28 days of P-407 administration and after PBS treatment for 28 days. Magnification as indicated. (B) Quantitative analysis of CD68^+^ cells in (A) specimens showing accumulation of CD68^+^ macrophages in the P-407-treated animals. For liver and heart, data are expressed as mean percentage ± SE of the brown-stained area in a selected field/total field area (five different fields per section). For the kidney, data represent mean percentage ± SE of the brown-stained glomerular area/total glomerular area for 20 glomeruli per section. Values represent the mean ± SE of at least four mice per group. p value by unpaired t test is shown (^∗^p < 0.05, ^∗∗^p < 0.01, and ^∗∗∗^p < 0.001). (C) Representative images of the Ki-67 staining (green) and CD68 (red) of the sections (A) following 28 days of treatment with P-407 or PBS. Splenic sections were used as positive controls, and Ki-67^+^ cells are indicated with arrows. Scale bars, 75 μm. See also Figure S4.

To extend the intravital microscopy findings to other organs, we then stained liver, heart, and kidney with an antibody against CD68, a widely used marker for tissue macrophages. The immunohistochemical quantification at different time points revealed a striking and gradually increasing accumulation of CD68^+^ cells in the P-407-injected animals compared to the PBS-treated controls (Figures 4A and 4B). F4/80 staining of the liver confirmed the increased presence of macrophages (Figure S4C). In agreement with the intravital findings, the number of CD68^+^ cells was already significantly increased 1 week after P-407 injections.

As recent papers have highlighted the self-renewal properties of tissue macrophages (Epelman et al., 2014), we used Ki-67, a well-known marker of proliferation, and found no evidence of proliferation (Figure 4C). Collectively, these data indicated that the drop in Gr1^low^ blood monocytes under hyper-TGRL conditions could be due to increased extravasation and tissue accumulation.

### Gr1^low^ Monocytes Are Recruited into Tissues under Increased TGRL Conditions

**Figure 5:**
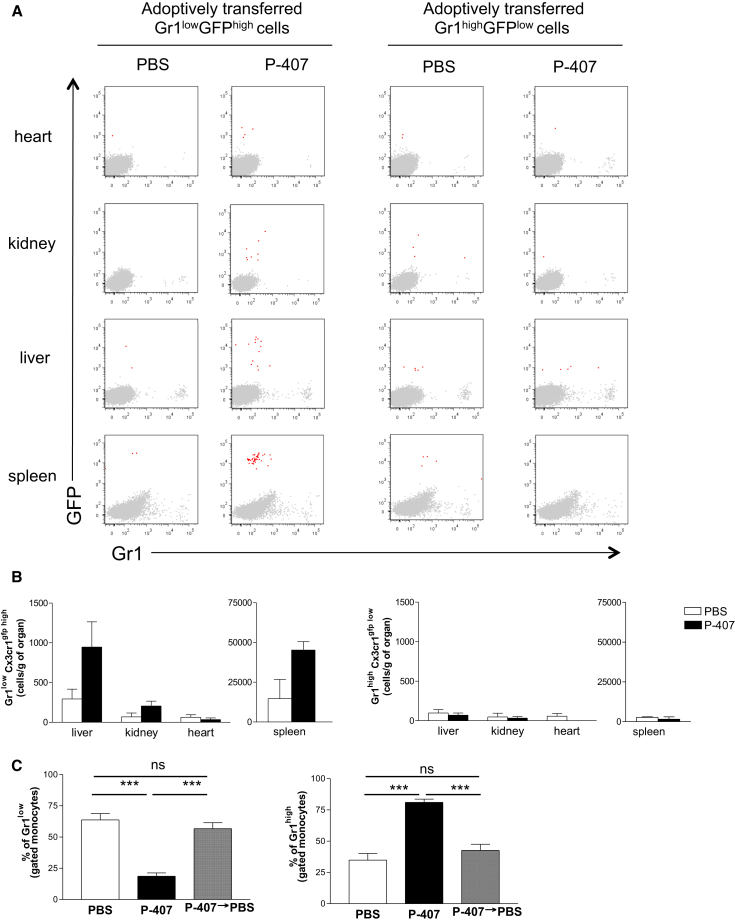
Tracing Monocyte Subset Migration by Adoptive Transfer (A) Gr1^low^GFP^high^ or Gr1^high^GFP^low^ blood monocytes were sorted from CD45.2 *Cx3cr1*^gfp/gfp^ mice. 0.1 × 10^6^ Gr1^low^GFP^high^ or Gr1^high^GFP^low^ sorted monocytes were injected intravenously into CD45.1.B6 mice. At 16 hr, mice were injected with CD11b antibody to exclude blood contamination. Mice were perfused with PBS, and organs (liver, heart, kidney, and spleen) were analyzed for monocyte migration. Dot plot overlays display total cells (gray) and CD45.2^+^CD11b^−^GFP^+^ cells (red). (B) Quantitative representation of (A). Data are from two independent experiments (n = 4); values represent the mean ± SE of number of cells per gram of organ. (C) B6 mice were treated with P-407 for 21 days or for 14 days followed by 7-day treatment with PBS. Mice treated with PBS for 21 days were used as controls. Data are expressed as percentage of Gr1^low^ and Gr1^high^ blood monocytes. Values represent mean ± SE, n = 4 mice per group. Significant p values are indicated (^∗∗∗^p < 0.001; ns, nonsignificant; unpaired t test). See also Figure S5.

To formally demonstrate that Gr1^low^ monocytes were indeed trafficking into the organs in the P-407-treated mice, we performed BM transplant and adoptive transfer experiments using the *Cx3cr1*^gfp/gfp^ mice. Two months after the engraftment of the *Cx3cr1*^gfp/gfp^ BM cells into B6 recipients, we detected a large number of GFP^+^ cells in all the organs prior to any P-407 intervention (Figure S4D), probably as a result of the damage caused by irradiation and repopulation of tissue resident macrophages from BM progenitors (Hashimoto et al., 2013), and thus, we abandoned this approach. We next adoptively transferred Gr1^low^GFP^high^ or Gr1^high^GFP^low^ monocytes, isolated from CD45.2*Cx3cr1*^gfp/gfp^ mice, into CD45.1B6 animals pretreated with either P-407 or PBS for 2 weeks. Recipients were sacrificed 16 hr after the adoptive transfer. To exclude the contamination from circulating monocytes, we also injected an anti-CD11b antibody that labeled blood monocytes immediately prior to the perfusion of the organs. Adoptively transferred Gr1^low^GFP^high^ monocytes were detected in the liver, spleen, and kidney of P-407-treated animals to a much greater extent than in the PBS-treated recipients (Figures 5A and 5B). There was no obvious recruitment in the heart, possible because of different kinetics of macrophage accumulation (Figure 5B). Of note, we found no increase of Gr1^high^GFP^low^ monocytes in the P-407-treated organs (Figures 5A and 5B), corroborating our earlier assumption that it was mainly the Gr1^low^ subpopulation that had extravasated in the tissues in response to the hyper-TGRL environment. Furthermore, Gr1^low^GFP^high^ monocytes isolated from P-407-treated CD45.2*Cx3cr1*^gfp/gfp^ mice did not extravasate when adoptively transferred into PBS-treated recipients (Figure S5A). The monocyte expression of CD11b, CCR2, CD68, LFA-1, and CCR5 (Figures S5B–S5F) did not change in response to the P-407 treatment, demonstrating that neither the P-407-induced hyper-TGRL environment nor the compound itself had altered the phenotype of the monocytes. To determine if the TGRL-induced drop in the number of Gr1^low^ monocytes was a reversible process, we treated mice with P-407 for 2 weeks and then stopped the treatment. On stopping the P-407 administration, lipid levels returned to normal by 72 hr (see Figure S1) and the Gr1^low^ fraction returned to pre-treatment levels by 2 weeks (Figure 5C).

**Figure 6:**
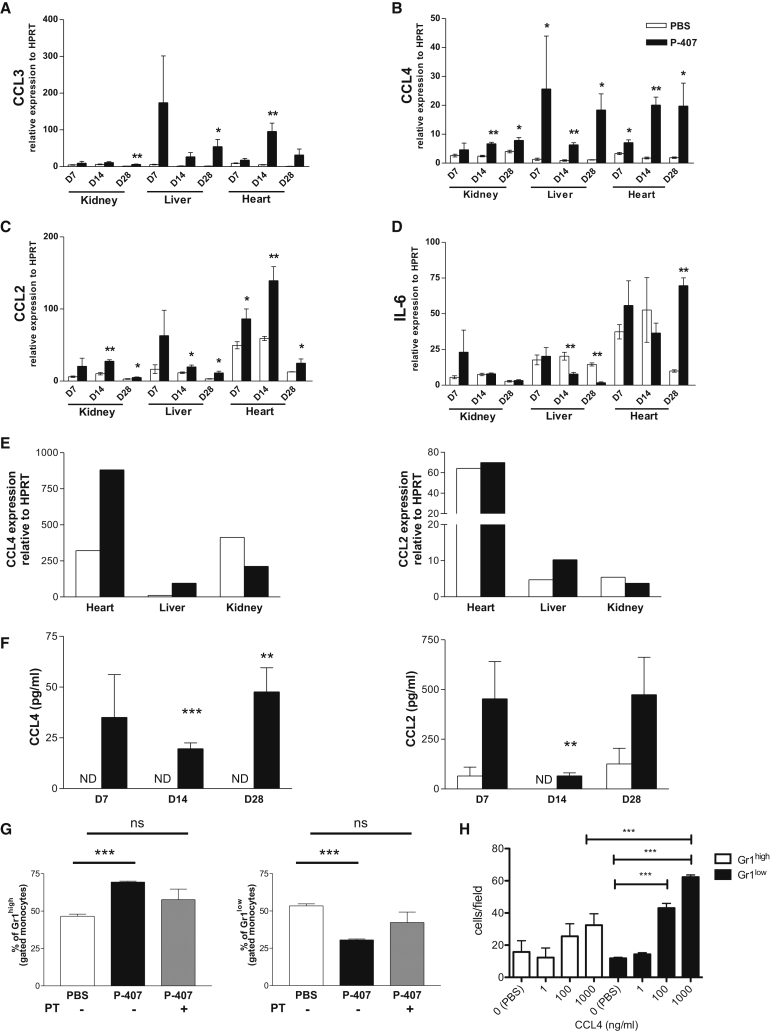
Chemokine Expression Triggered by P-407 Treatment (A–D) The effect of P-407 on mRNA expression of chemokine/cytokine was analyzed by RT-PCR. Relative expression of (A) CCL3, (B) CCL4, (C) CCL2, and (D) IL-6 to the housekeeping gene HPRT. Kidney, liver, and heart specimens from mice treated with P-407 were compared with those from PBS-treated animals at each time point. (E) CCL4 and CCL2 mRNA expression relative to HPRT in CD45^+^F4/80^+^ sorted tissue macrophages from liver, heart, and kidney after 14 days of P-407 or PBS treatment. Data are pooled from three animals per condition and representative of two independent experiments. (F) Plasma concentrations of CCL4 and CCL2 after 7, 14, and 28 days of P-407 or PBS treatment. Values represent mean ± SE, n = 4 mice per group. (G) Frequencies of Gr1^high^ and Gr1^low^ monocyte subsets in P-407-treated B6 mice injected with PT or PBS. PBS-treated mice were used as controls. Values represent mean ± SE, n = 3 mice per group. Results are representative of three independent experiments. Significant p values are indicated (^∗^p < 0.05, ^∗∗^p < 0.01, and ^∗∗∗^p < 0.001; unpaired t test). ND, nondetectable; ns, nonsignificant. (H) Transwell migration assay. Fluorescence-activated cell-sorted Gr1^high^ and Gr1^low^ monocytes were added to a transwell chamber for 2 hr in the presence of recombinant mouse CCL4 (1–1,000 ng/ml) or PBS (0). The number of migrated cells per field was quantified (five fields per sample); pool of n = 3 mice in triplicate. Data are mean ± SE. ^∗∗∗^p < 0.001 (unpaired t test). See also Figure S6.

To investigate the mechanism(s) of the extravasation we quantified tissue mRNA expression of a large panel of chemokines/cytokines. As shown in Figure 6, this analysis showed a marked upregulation of CCL4 in the organs from the P-407-treated animals compared to those from the PBS controls. CCL2 and CCL3 also showed some increased expression. Of note, IL-6 mRNA expression showed a very different pattern and was reduced in the liver from P-407-treated animals (Figure 6D), confirming that the P-407 treatment did not elicit an overt inflammatory response. In keeping with the intravital data, the expression of CX3CL1 (fractalkine) was not affected by the P-407 treatment (Figure S6A). Similarly, other chemokines such as CXCL9, CXCL1, and CCL5 were expressed at similar levels in the two experimental groups (Figures S6B–S6D). As macrophages can produce a large amount of CCL4 (Maurer and von Stebut, 2004) and were increased in tissues following P-407 treatment, we hypothesized that they could represent a potential source of the CCL4. We therefore analyzed CCL4 mRNA expression in sorted CD45^+^F4/80^+^ tissue macrophages after 2 weeks of PBS or P-407 treatment. We found that macrophages from heart and liver of P-407-treated animals had a more than 2-fold increase in CCL4 expression compared to those from PBS-treated controls (Figure 6E). We observed only a slight increase in CCL2 expression in the liver macrophages from P-407-treated mice (Figure 6E). We then measured CCL2 and CCL4 plasma levels. In keeping with the gene expression data, we detected CCL4 only in the animals treated with P-407 and not in the PBS controls (Figure 6F), demonstrating that a hyper-TGRL environment could induce the production of this chemokine. We also detected an increase in CCL2 levels in the P-407 mice, but the increase reached statistical significance only at day 14 (Figure 6F).

CCR5, which binds CCL4 and CCL3, was not selectively expressed in Gr1^low^ monocytes, regardless of the lipid levels (Figure S5F), making it an improbable candidate. Moreover, CX3CR1 expression in both monocyte subsets was lower following P-407 treatment, confirming that CX3CL1 signaling was not driving the extravasation (Figure S5G). To support the idea that a chemokine/chemokine receptor axis may contribute to the migration of Gr1^low^ monocytes into the peripheral tissue in P-407-treated mice, we assessed whether the drop in the Gr1^low^ compartment could be rectified by PT, a potent inhibitor of Gα_i_-coupled receptor signaling, including chemokine receptors. A single administration of PT after 10 days of P-407 treatment prevented further extravasation of the Gr1^low^ monocytes, and the percentage of Gr1^low^ monocytes increased to levels similar to those in PBS-treated mice (Figure 6G). Additionally, transwell chemotaxis assays with fluorescence-activated cell-sorted Gr1^low^ or Gr1^high^ monocytes and recombinant mouse CCL4 confirmed that CCL4 preferentially induces migration of Gr1^low^ monocytes (Figure 6H), while CCL2 induces mainly migration of Gr1^high^ (Figure S6E). Collectively, these findings suggest that elevated levels of TGRLs are capable of promoting a differential migration of blood Gr1^low^ monocytes into the tissue that is likely to be mediated, at least in part, by CCL4.

## Discussion

In this study, we show that increased levels of TGRLs promote the migration of Gr1^low^ monocytes from the blood compartment into the surrounding tissue. The drop in the blood Gr1^low^ subset was accompanied by an increased crawling of these monocytes at the endothelial interface and a striking accumulation of CD68^+^ tissue macrophages in the heart, liver, and kidney. The changes in the monocyte subset distribution were not driven by systemic inflammation but were mediated, at least in part, by chemokines, mainly CCL4, via G-protein-coupled receptor(s).

While the cardiovascular disease (CVD) risks associated with increased levels of cholesterol-enriched lipoproteins, in particular low-density lipoprotein (LDL), are well known and have been extensively studied, the health-related consequences of elevated levels of TGRLs such as very low-density lipoprotein (VLDL) have been less well characterized and remain controversial (Sarwar et al., 2007). Increased TGRL levels are a key feature of the metabolic syndrome (Ninomiya et al., 2004) and may contribute to the associated morbidity. In addition, high TGRL levels are increasingly recognized as an independent risk factor for CVD. To investigate the impact of a hyper-TGRL environment, we used a murine model of P-407-induced hyper-TGRL (Johnston, 2004). We considered that the P-407 model was informative as (1) it induces a predominant increase in plasma TGRL, reaching levels similar to those detected in patients with familial hypertriglyceridemia or other genetic disorders of the triglyceride metabolism (Benlian et al., 1996; Pejic and Lee, 2006); (2) there is no overt inflammatory response, a confounding factor in other model of murine hyperlipidemia (Getz and Reardon, 2006); and (3) P-407 did not induce monocytosis or changes in monocyte surface phenotype, as previously reported in *Ldlr*^−/−^ or *ApoE*^−/−^ mice (Swirski et al., 2007; Tacke et al., 2007; Wu et al., 2009) and in human monocytes after a lipid-rich meal (Foster et al., 2013; Gower et al., 2011). Using the P-407 model, we demonstrated the migration of non-classical Gr1^low^ monocytes into surrounding tissues.

The relationship between blood monocytes and tissue macrophages remains enigmatic. There is evidence that, under steady-state conditions, monocytes are restricted to the blood compartment and adult tissue macrophages are embryonic in origin (Ginhoux and Jung, 2014). However, in certain tissue compartments, such as the skin and most notably the gut (Jaensson et al., 2008; Bain et al., 2014), there are experimental data indicating that circulating monocytes contribute significantly to the tissue macrophage compartment. These diverse findings suggest that blood monocyte migration to tissue compartments is influenced by environmental and cell-specific factors. The majority of the work has focused on the classical Gr1^high^ monocytes that can rapidly respond to inflammatory and bacterial signals. However, non-classical Gr1^low^ monocytes make up 40%–50% of the monocyte population in mice and ∼10% in humans (Yona and Jung, 2010), and their functions are less well defined. There are reports that they can migrate in atherosclerotic settings (Nahrendorf et al., 2007) and can respond to viral and TLR7/8 cues (Carlin et al., 2013b; Cros et al., 2010), but it is their ability to patrol or survey the endothelial interface (Auffray et al., 2007; Carlin et al., 2013b; Cros et al., 2010) that has become widely recognized. Whether they can migrate and contribute to tissue macrophages under steady-state conditions remains unclear. Unexpectedly, our data indicated that the behavior of Gr1^low^ monocytes was markedly altered within a high-TGRL environment. We observed that in a high-TGRL environment, Gr1^low^ monocytes were depleted from the blood due to their retention at the endothelial interface. This was accompanied by a marked accumulation of CD68^+^ tissue macrophages in the heart, liver, and kidney. Adoptive transfer experiments of fluorescence-activated cell-sorted monocytes showed that, under our experimental conditions, Gr1^low^ monocytes were able to extravasate. This finding, together with the results of the Ki67 staining, ruled out the possibility that the increased number of tissue macrophages was due to in situ proliferation, as noted during inflammation (Davies et al., 2013).

We excluded the possibility that our findings were a direct effect of P-407. Consistent with the findings in patients with familial hypercholesterolemia (Mosig et al., 2009), we observed a similar drop in the Gr1^low^ monocytes in pre-atherosclerotic *Ldlr*^−/−^ mice. Increased numbers of tissue macrophages have previously been described in high-fat-fed models (Fink et al., 2014; Lohmann et al., 2009). The production of monocytes is finely tuned by BM stem cell precursors, which can be influenced by dyslipidemia (Murphy et al., 2011). However, the P-407-induced increase in TGRLs did not cause obvious changes in the number of cMoPs or BM mature monocytes. Moreover, splenectomy did not alter the response of the Gr1^low^ monocytes to the hyper-TGRL environment, excluding splenic scavenging or extramedullary hematopoiesis as potential mechanisms. Collectively, our observations point toward a scenario where non-classical Gr1^low^ monocytes specifically respond to a rise in TGRL levels and, in the absence of overt inflammatory cues, extravasate into peripheral tissue and organs, contributing to the increased number of tissue CD68^+^ and F4/80^+^ macrophages. Our data, therefore, support the notion of a more dynamic role of Gr1^low^ monocytes in response to dyslipidemia rather than being just intravascular housekeepers or “blood macrophages” (Carlin et al., 2013b; Yona and Jung, 2010). Whether these cells extravasate in response to milder dyslipidemia or whether scavenging lipids in the tissue is part of their housekeeping functions requires further investigations.

Activation of Gr1^low^ monocytes and their human counterparts has been shown in murine lupus models and patients with SLE (Amano et al., 2005; Cros et al., 2010; Nakatani et al., 2010; Santiago-Raber et al., 2009; Yoshimoto et al., 2007), suggesting that Gr1^low^ monocytes might contribute to tissue injury (Misharin et al., 2014). In support of this, blockade of CX3CR1, which is highly expressed on Gr1^low^ monocytes, reduced monocyte recruitment to the kidney and the resulting inflammation (Inoue et al., 2005; Nakatani et al., 2010). Therefore, it is tempting to speculate that in conditions where there is abnormal lipid metabolism, such as chronic renal diseases, extravasated Gr1^low^ monocytes may convert into CD68^+^/F480^+^ macrophages and contribute to tissue damage. Monocyte-specific lineage reporters will be necessary to uncover the contribution of Gr1^low^ monocytes to tissue homeostasis and pathology under hypertriglyceridemia.

We identified CCL4 as one of the potential molecules mediating the changes in the distribution of blood monocytes during hypertriglyceridemia. CCL4 is known to bind CCR5, a chemokine receptor expressed by both monocyte subpopulations (Tacke et al., 2007; Weber et al., 2000), a finding confirmed by us. TGRL levels did not alter the monocyte phenotype or modulate CCR5 expression, making it an unlikely candidate for the CCL4-mediated effect. However, administration of PT rectified some of the drop in blood Gr1^low^ monocytes, confirming that a Gα_i_ chemokine-receptor axis was involved. CCL4 can be produced by different cell types, including macrophages (Maurer and von Stebut, 2004). We found that CCL4 expression by macrophages from the heart and liver was enhanced in the hyper-TGRL environment. This suggests that a positive feedback loop may exist: foam cell formation, triggered by the hyperlipidemia, initiates Gr1^low^ monocyte recruitment that in turn enhances foam cell formation, perpetuating the process. Considering that CCL4 has been shown to be expressed by macrophages in response to modified LDL (Wiesner et al., 2010) and that its receptor, CCR5, mediates Gr1^low^ recruitment in atherosclerosis (Tacke et al., 2007), our data raise the possibility that this chemokine may play a key role in the increased CVD risk observed in chronic diseases associated with elevated TGRLs.

In conclusion, our study demonstrates that high TGRL levels alter monocyte subset distribution by promoting the extravasation of the non-classical Gr1^low^ subset. These findings highlight the impact that an abnormal TGRL profile may have on the intravascular and extravascular behavior of blood monocytes.

## Experimental Procedures

### Mice

C57BL/6, C57BL/6.CD45.1, C57BL/6.LDLR-deficient (*Ldlr*^−/−^), and B6.129P-Cx3cr1tm1Litt/J (*Cx3cr1*^gfp/gfp^) mice were used.

### Poloxamer 407 Administration

Mice were injected i.p. with 10 mg Poloxamer 407 (Pluronic F-127, Sigma-Aldrich) solution or PBS every second day.

### In Vitro Experiments

PBMCs from B6 mice were cultured overnight in the presence of P-407 concentration ranging from 50 μg/ml to 10 mg/ml and then stained for propidium iodide (PI) and Annexin V. PBMCs from B6 mice treated with PBS or P-407 for 28 days were stained for neutral lipid using the LipidTox kit (Invitrogen).

### Cell Sorting and Adoptive Transfer Experiment

0.1 × 10^6^ of fluorescence-activated cell-sorted Gr1^low^GFP^high^ or Gr1^high^GFP^low^ monocytes from CD45.2 *Cx3cr1*^gfp/gfp^ were injected intravenously into congenic CD45.1 mice. Organs were collected 16 hr later.

### PT

Mice treated for 10 days with P-407 were injected intravenously with 0.2 μg PT (Tocris Bioscience) or PBS. One day later, monocytes subsets were assessed by fluorescence-activated cell sorting analysis.

### Splenectomy

Splenectomy was performed, and 4 weeks later, mice were treated with P-407 or PBS for 2 weeks. Monocyte subsets were then assessed by flow cytometry.

### BrdU Pulsing

Three doses of BrdU (BD Pharmingen) were administered i.p. 3 hr apart. BrdU incorporation in blood monocytes was assessed by bleeding the mice 1, 3, and 5 days following BrdU injection.

### Intravital Microscopy

Intravital microscopy was performed in mesentery and dermal ear microcirculation of *Cx3cr1*^gfp/+^ and *Cx3cr1*^gfp/gfp^ mice as previously described (Carlin et al., 2013a). See Supplemental Experimental Procedures for details.

### Transwell Migration Assay

Gr1^low^ and Gr1^high^ blood monocytes were sorted using Aria II FACS (Becton-Dickson). 1 × 10^5^ cells per well were seeded into 3-μm-pore transwell inserts (Corning), using a chemoattractant gradient of 1–1,000 ng/ml of recombinant mouse CCL4 or CCL2 (R&D Systems) or PBS and incubated at 37°C for 2 hr. Transwell inserts were fixed and slides were imaged using a 10× objective on an Olympus BX51 widefield fluorescence microscope and nuclei were manually counted from five fields.

### Statistical Analysis

Comparisons between two groups were performed using two-tailed unpaired Student’s t test or Mann-Whitney test as indicated in the figure legend. Statistically significant is defined as p < 0.05. N for each experiment is given in the figure or figure legends. ^∗^p < 0.05; ^∗∗^p < 0.01, ^∗∗∗^p < 0.001.

## Author Contributions

M.F.S. and L.B. conducted the experiments and analyzed the data; T.M., L.F.-J., M.R., and W.D.J. performed some experiments; M.C.P. assisted with data interpretation and edited the paper; H.T.C. analyzed the histology; and K.J.W and M.B. designed the experiments and wrote the paper.

## Supplemental Information

Document S1. Supplemental Experimental Procedures and Figures S1–S6

Movie S1. Monocyte Patrolling in Control-Treated CX3CR1^GFP^ Ear Dermis, Related to Figure 3 Green is CX3CR1^high^ (GFP^+^) monocyte/macrophage. Red, 70 kDa dextran. Scale bar, 50 μm. Time, hr:min:s.

Movie S2. Monocyte/Macrophage in 7-Day P-407-Treated CX3CR1^GFP^ Ear Dermis, Related to Figure 3 Green is CX3CR1^high^ (GFP^+^) monocyte/macrophage. Red, 70 kDa dextran. Scale bar, 50 μm. Time, hr:min:s.

Movie S3. Monocyte Patrolling in Control-Treated CX3CR1^GFP^ Mesentery Venule, Related to Figure 3 Green is CX3CR1^high^ (GFP^+^) monocyte/macrophage. Scale bar, 50 μm. Time, hr:min:s.

Movie S4. Monocyte Endothelial Accumulation in 7-Day P-407-Treated CX3CR1^GFP^ Mesentery Venule, Related to Figure 3 Green is CX3CR1^high^ (GFP^+^) monocyte/macrophage. Red, 70 kDa dextran. Scale bar, 50 μm. Time, hr:min:s.

Document S2. Article plus Supplemental Information
