# Supplementary material for: Triglyceride-Rich Lipoproteins Modulate the Distribution and Extravasation of Ly6C/Gr1low Monocytes
Source: Cell Rep. 2015 Sep 3;12(11):1802–15. doi: 10.1016/j.celrep.2015.08.020 (PMC4590546; doi:10.1016/j.celrep.2015.08.020)
Supplement: Document S1. Supplemental Experimental Procedures and Figures S1–S6 [file mmc1.pdf]

Cell Reports

Supplemental Information

Triglyceride-Rich Lipoproteins Modulate the Distribution and Extravasation of Ly6C/Gr1^{low} Monocytes

Maha F. Saja, Lucie Baudino, William D. Jackson, H. Terence Cook, Talat H. Malik, Liliane Fossati-Jimack, Marieta Ruseva, Matthew C. Pickering, Kevin J. Woollard, and Marina Botto

Supplemental figure legends

Figure S1. P-407-induced dyslipidemia, Related to Figure 1. Total plasma TG (**A**) and CHOL (**B**) in B6 mice injected i.p. thrice weekly for 28 days with 10mg (equivalent to 0.5 g/kg) of P-407 or PBS while consuming a standard chow diet. TG and CHOL were always measured 24hrs after the P-407 injection and were significantly increased at each time point. Data are expressed as mean ±SE, n=4, unpaired t-test. Data representative of 2 independent experiments. *P<0.05, **P<0.01,*** P<0.001. (**C** and **D**) TG (**C**) and CHOL (**D**) levels following a single i.p. injection of P-407 (0.5 g/kg) or PBS. Lipid levels were measured at 0, 6, 24, 48, 72, and 96 hours post injection. Lipid concentrations peaked at 24hrs post P-407 injection and dropped sharply by 48hrs. Data are expressed as mean ±SE, n=3 in P-407 treated group. (**E** and **F**) Lipoprotein distribution of TG (**E**) and CHOL (**F**) in P-407-induced dyslipidemia assessed by chromatography on a Superose 6 HR 10/30 size-exclusion column. Pooled plasma (n=3 for each group) after 28 days. Very-low density lipoprotein (VLDL) corresponds to fractions 5 to 10, LDL to fractions 11-19 and high-density lipoprotein (HDL) to fractions 20-30. (**G**) Serum levels of apolipoprotein C-III (APOC3) and apolipoprotein E (APOE) after 7, 14 and 28 days of treatment with PBS or 0.5 g/kg P-407. Data are expressed as mean ±SE, n=4, unpaired t-test. *P<0.05, **P<0.01, ***P<0.001. (**H**) Circulating levels of IL-6, TNF-α and serum amyloid P component (SAP) at different time points during P-407 treatment. Results are expressed as mean ±SE, n=4 per time point, unpaired t-test. **P<0.01, ns = non significant. (**I** and **J**) Body (**I**) and spleen (**J**) weights at baseline and after 28 days of 0.5 g/kg P-407 treatment. Data are expressed as mean ±SE, n=6, unpaired t-test, **P<0.01. (**K**) Representative staining of liver (PAS), kidney, heart and spleen (H&E) after 28 days of P-407 injections. Arrows indicate foam cells in the P-407-treated group.

Figure S2. Peripheral blood cells in P-407-treated mice and blood monocyte distribution in high fat fed *Ldlr-/-* mice, Related to Figure 1 Quantitative analysis (numbers and percentages) of white blood cells (WBCs) (**A**), monocytes (**B**), T cells (**C** and **D**), B cells (**E** and **F**), PMNs (**G** and **H**) in mice injected with P-407 (closed circles) or PBS (open circles) over a period

of 28 days. Values represent mean ±SE, pooled data from 4 independent experiments, at least 7 mice per group at each time point, unpaired t-test. (**I**) Degree of survival of peripheral blood mononuclear cells over a range of P-407 concentrations (from 50 mg/ml to 10mg/ml). Frequency of Annexin $V^{+}PI^{+}$ monocytes (gated $CD11b^{+}CD115^{+}$ cells) at each P-407 concentration is represented. Representative data of 3 independent experiments. (**J** and **K**) Total plasma TG (**J**) and CHOL (**K**) in *Ldlr*-/- mice on high fat (HF) or low fat (LF) diets at day 0 and day 50. (**L**) Total number of blood monocytes in HF- or LF-fed *Ldlr*-/- mice after 50 days. (**M** and **N**) Quantitative analysis (numbers and percentages) of $Ly6C^{high}$ (equivalent to $Gr1^{high}$) and $Ly6C^{low}$ (equivalent to $Gr1^{low}$) monocytes in *Ldlr*-/- mice on a HF or LF diet for 50 days. (**J**-**N**) Data are expressed as mean ±SE, n=5, unpaired t-test. *P<0.05; **P<0.01, ***P<0.001.

Figure S3. Splenectomy and BrdU pulse experiment, Related to Figure 2. (**A**) Number of $Gr1^{high}$ and $Gr1^{low}$ monocytes after splenectomy in mice treated with PBS or P-407 for 14 days. Results are expressed as mean ±SE, n=3, *** P<0.0001, ns = non significant. (**B**) Representative dot plots showing BrdU incorporation into $Gr1^{high}$ and $Gr1^{low}$ blood monocytes over a period of 5 days following a single pulse of BrdU administered i.p. in three doses of 2mg, 3 hours apart. Percentages of BrdU incorporation in $Gr1^{low}$ monocytes (red rectangle) and $Gr1^{high}$ monocytes (blue rectangle) are indicated.

Figure S4. Behaviour of GFP^{+} cells in extravascular tissue and quantitative analysis of $F4/80^{+}$ cells, Related to Figures 3-4. Migratory behaviour of GFP^{+} cells in extravascular tissue of P-407- and PBS-treated C*x3cr1*$^{gfp/+}$ mice; **(A)** ear dermis: velocity, displacement and number of GFP+ cells; **(B)** mesentery tissue: velocity and displacement of GFP+ cells. **(C)** Representative photomicrographs and quantitative analysis of F4/80 staining (brown) of liver sections from PBS- and P-407-treated mice (14 days). Data expressed as mean percentage ±SE of brown-stained area in a selected field/total field area (5 fields per section), n=3. Data expressed as mean ±SE, n=4, unpaired t-test. * P<0.05; ***P<0.001, NS = non significant. **(D)** Bone marrow transplant. B6 mice were reconstituted with bone marrow cells from *Cx3cr1*$^{gfp/gfp}$ mice. Two months later, prior to any treatment, two mice were

culled and tissue macrophages analysed. Representative dot plots showing GFP$^+$ and F4/80$^+$ cells (gated CD45$^+$ cells).

Figure S5. Monocyte adoptive transfer and expression of surface markers on peripheral blood monocyte subsets following P-407 treatment, Related to Figure 5. (**A**) FACS-sorted Gr1^{low}GFP^{high} (0.1x10^6) from CD45.2*Cx3cr1*$^{gfp/gfp}$ mice were injected i.v. into CD45.1B6 mice. 'P-407-recipient' represents PBS-exposed monocytes transferred into mice treated with P-407 for 14 days. 'P-407-donor' indicates monocytes isolated from P-407-treated mice (14 days) transferred to PBS-treated recipients. To exclude blood contamination an antibody against CD11b was injected prior to sacrificing the mice at 16hrs. Quantitative representation of number of cells in the liver per g of organ, n = 2-4, values represent the mean ±SE. **(B-F)** Expression of CD11b (**B**), CCR2 (**C**), CD68 (**D**), LFA1 (**E**) and CCR5 **(F)** on Gr1^{high} and Gr1^{low} monocytes in mice injected for 28 days with PBS or P-407. (**G**) CX3CR1 expression on Gr1^{high} and Gr1^{low} monocytes from *Cx3cr1*$^{gfp/+}$ mice. Values represent mean ±SE, unpaired t-test, n=3 per group, ns = non significant, *P<0.05; ***P<0.001. Data expressed as delta MFI (MFI=MFI-isotype control).

Figure S6. Chemokine expression triggered by P-407 treatment, Related to Figure 6. The effect of P-407 on mRNA expression of chemokine/cytokine was analysed by RT-PCR. Relative expression of (**A**) CX3CL1, (**B**) CXCL9, (**C**) CCL5, (**D**) CXCL1 to the house keeping gene HPRT. Kidney, liver, and heart specimens from mice treated with P-407 were compared with those from PBS-treated animals at each time point. Values represent mean ±SE, n= 4 mice per group, P values were all non significant, unpaired t-test. **(E).** FACS-sorted Gr1^{high} and Gr1^{low} monocytes (1x10^5) were added to 3µm transwells for 2hrs in the presence of recombinant mouse CCL2 (range 1-1000ng/ml) or PBS. The number of migrated cells/field was quantified (5 fields per sample), n=3 mice pooled in triplicate. Values represent mean ±SE, *P<0.05; *P<0.01 and ***P<0.001, unpaired t-test.

Figure S1

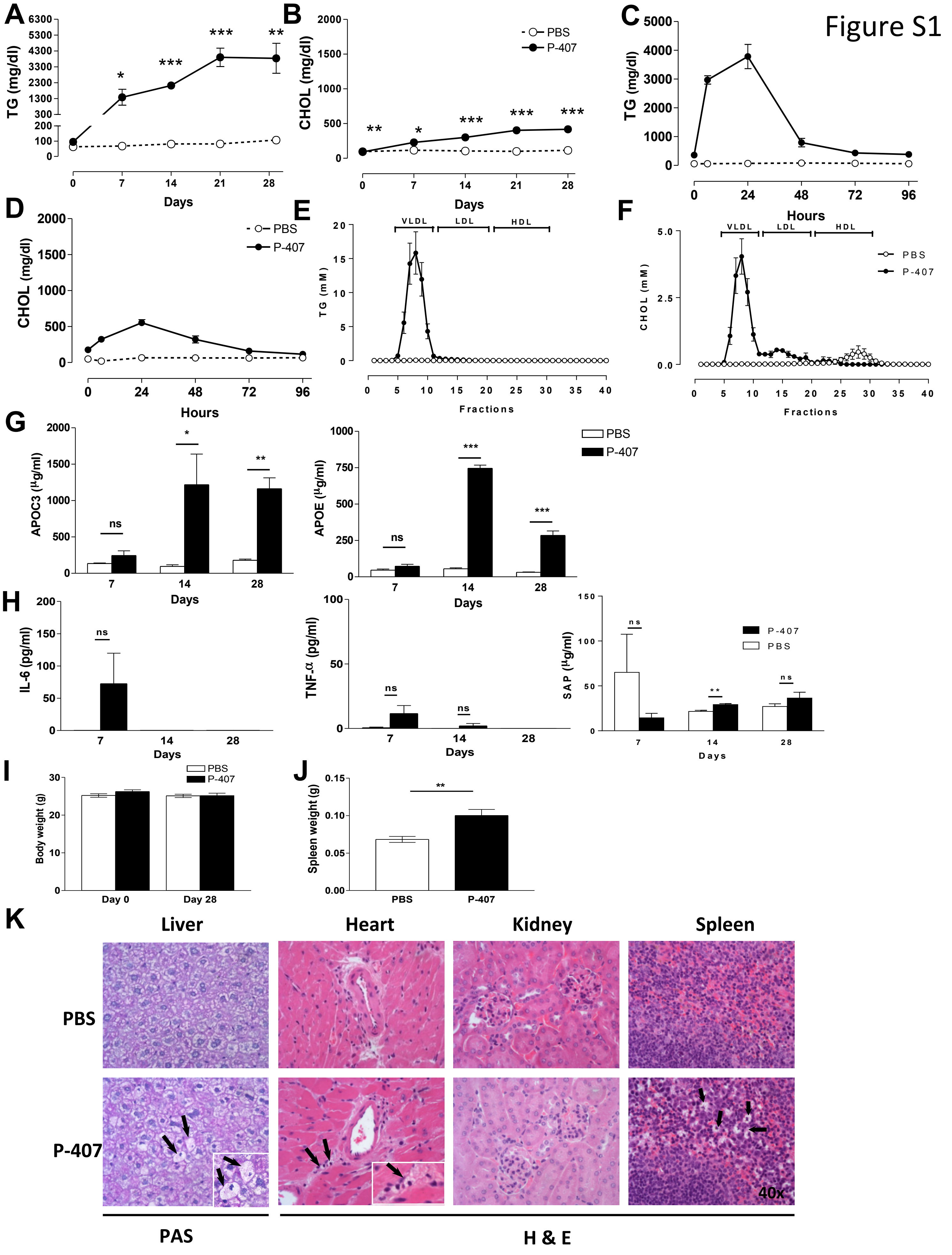
A
B
C
D
E
F
G
H
I
J
K
TG (mg/dl)
CHOL (mg/dl)
Days
Hours
PBS
P-407
VLDL
LDL
HDL
TG (mM)
CHOL (mM)
Fractions
APOC3 (μg/ml)
APOE (μg/ml)
IL-6 (pg/ml)
TNF-α (pg/ml)
SAP (μg/ml)
ns
Body weight (g)
Day 0
Day 28
Spleen weight (g)
Liver
Heart
Kidney
Spleen
40x
PAS
H & E

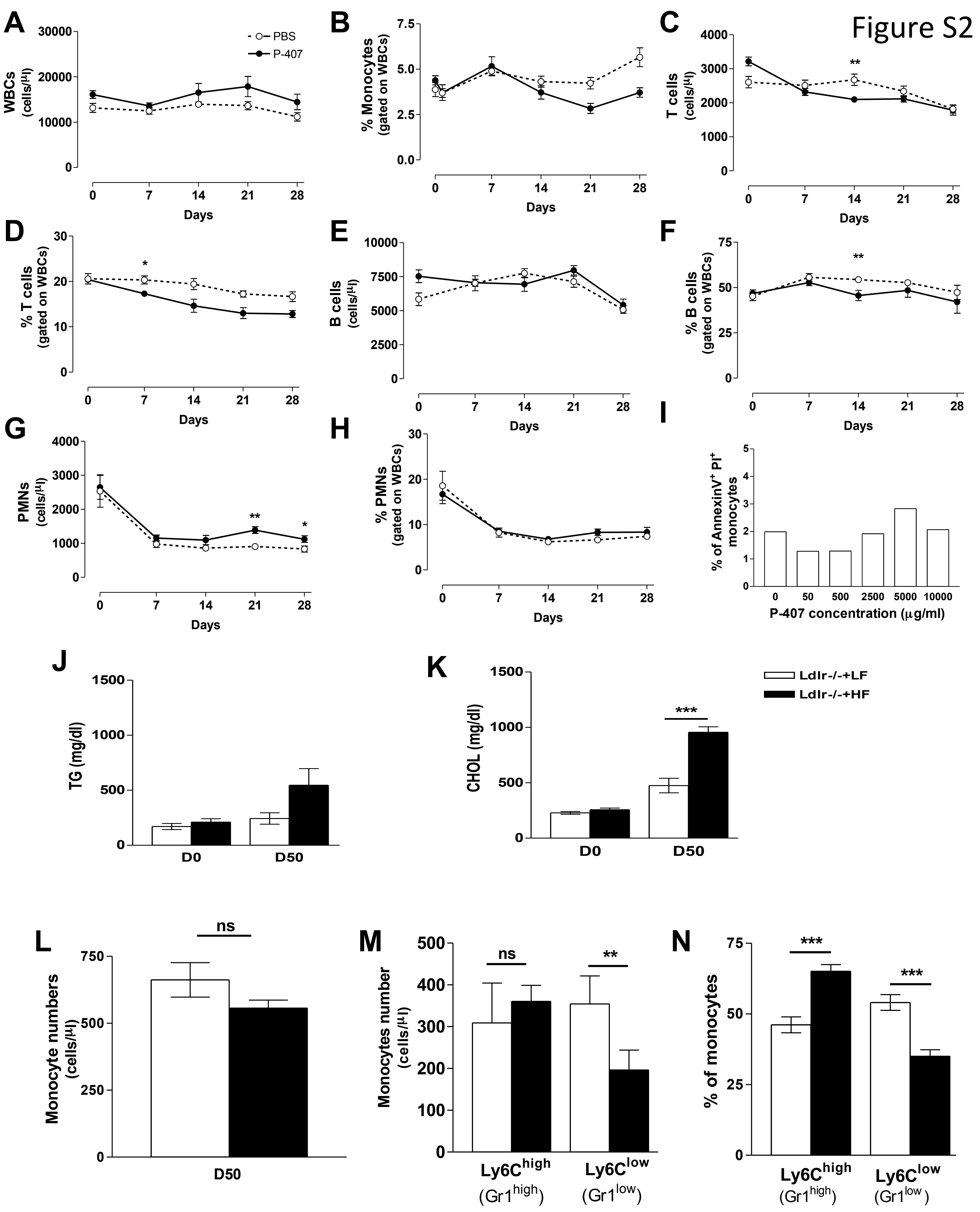

Figure S2
A
B
C
D
E
F
G
H
I
J
K
L
M
N
PBS
P-407
WBCs (cells/μl)
% Monocytes (gated on WBCs)
T cells (cells/μl)
% T cells (gated on WBCs)
B cells (cells/μl)
% B cells (gated on WBCs)
PMNs (cells/μl)
% PMNs (gated on WBCs)
% of AnnexinV+ PI+ monocytes
P-407 concentration (μg/ml)
Days
Ldlr-/-+LF
Ldlr-/-+HF
TG (mg/dl)
CHOL (mg/dl)
D0
D50
Monocyte numbers (cells/μl)
Monocytes number (cells/μl)
% of monocytes
Ly6C high (Gr1 high)
Ly6C low (Gr1 low)
ns

Figure S3

A

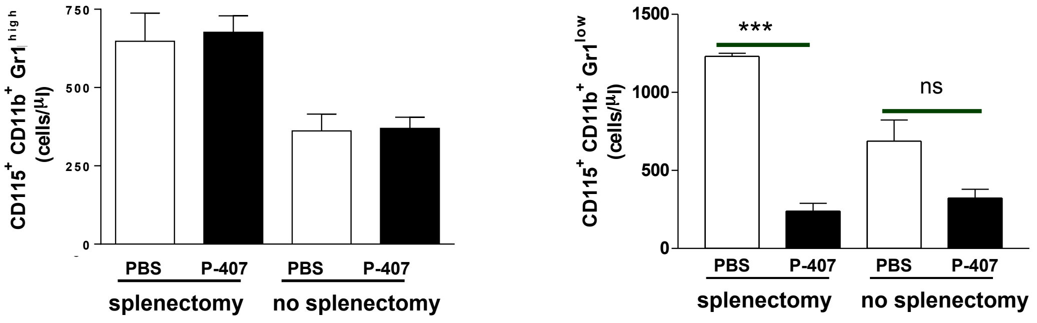
CD115+ CD11b+ Gr1 high (cells/µl)
750
500
250
0
PBS
P-407
PBS
P-407
splenectomy
no splenectomy
CD115+ CD11b+ Gr1 low (cells/µl)
1500
1000
500
0
***
ns
PBS
P-407
PBS
P-407
splenectomy
no splenectomy

B

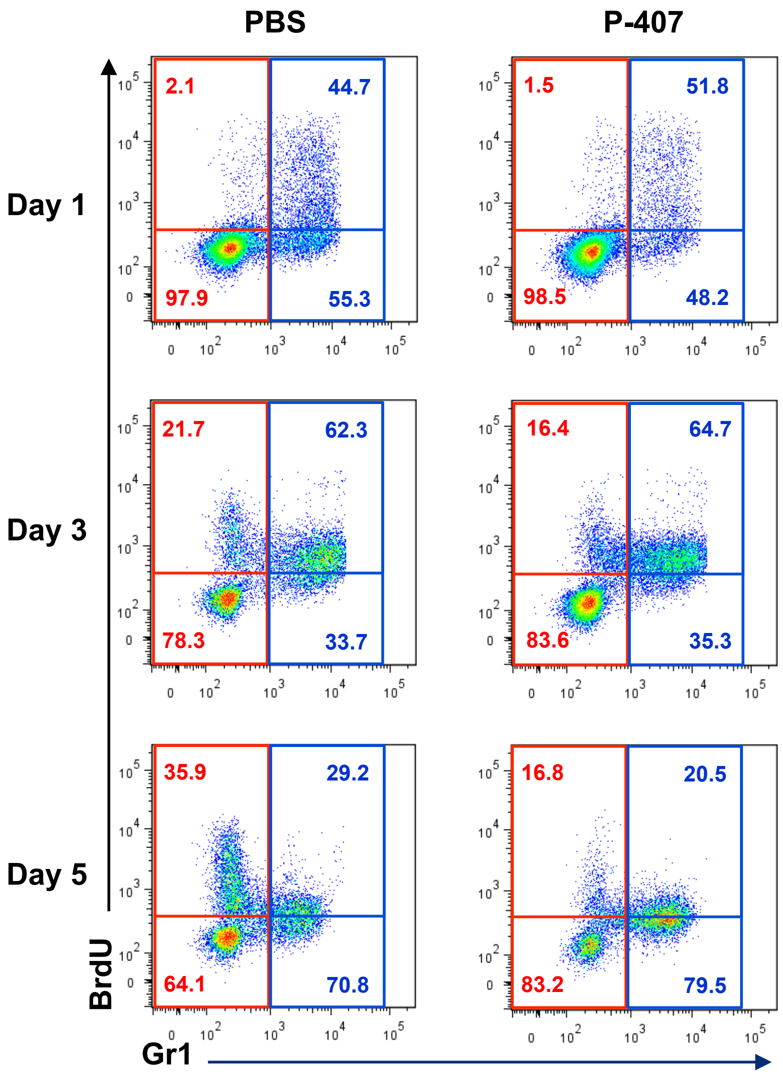
PBS
P-407
Day 1
Day 3
Day 5
BrdU
Gr1
2.1
44.7
97.9
55.3
1.5
51.8
98.5
48.2
21.7
62.3
78.3
33.7
16.4
64.7
83.6
35.3
35.9
29.2
64.1
70.8
16.8
20.5
83.2
79.5

Figure S4

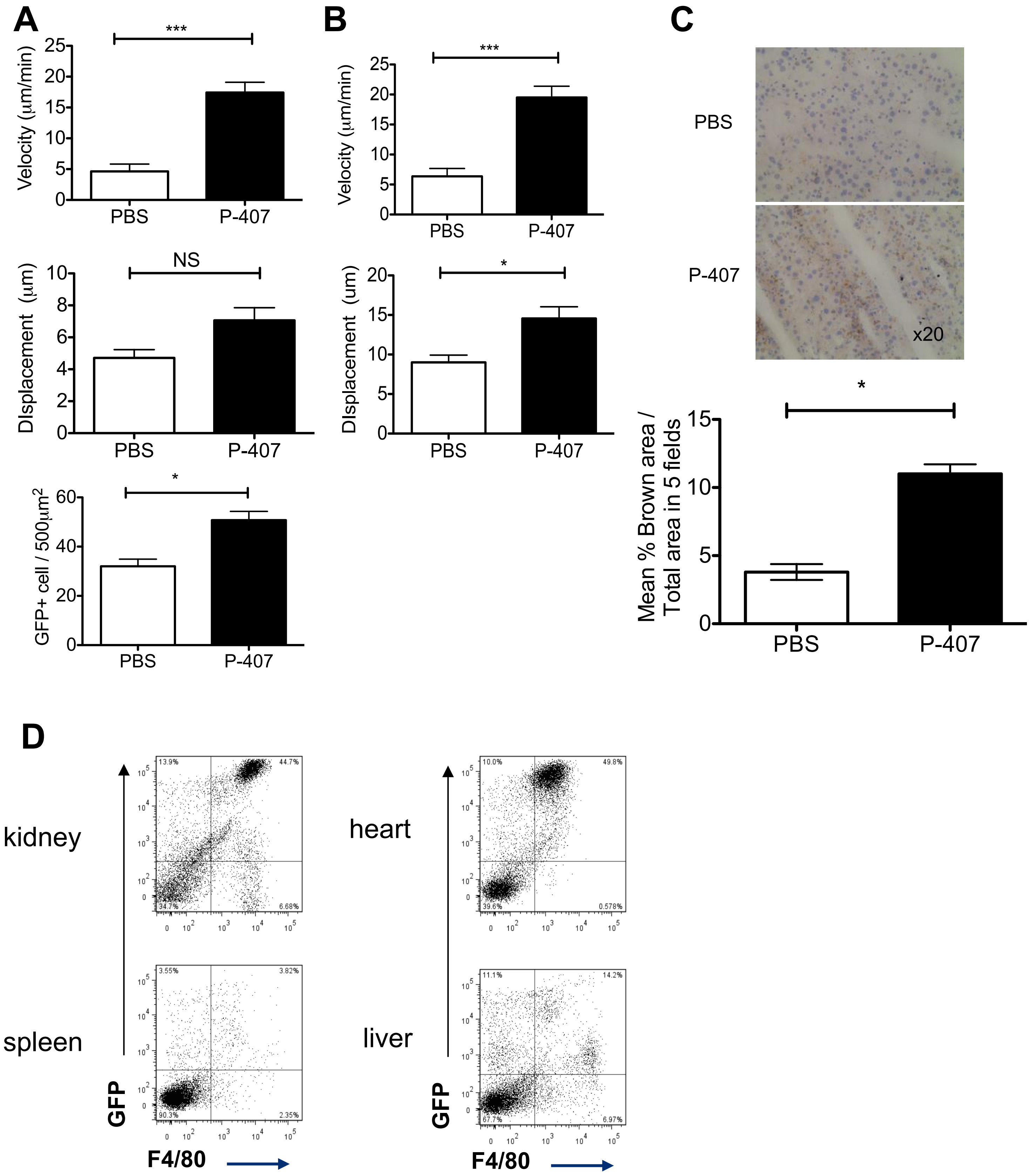
A
***
Velocity (μm/min)
25
20
15
10
5
0
PBS
P-407
NS
DIsplacement (μm)
10
8
6
4
2
0
PBS
P-407
*
GFP+ cell / 500μm2
60
40
20
0
PBS
P-407
B
***
Velocity (μm/min)
25
20
15
10
5
0
PBS
P-407
*
DIsplacement (um)
20
15
10
5
0
PBS
P-407
C
PBS
P-407
x20
*
Mean % Brown area / Total area in 5 fields
15
10
5
0
PBS
P-407
D
kidney
13.9%
44.7%
34.7%
6.68%
spleen
3.55%
3.82%
90.3%
2.35%
heart
10.0%
49.8%
39.6%
0.578%
liver
11.1%
14.2%
67.7%
6.97%
GFP
F4/80

Figure S5

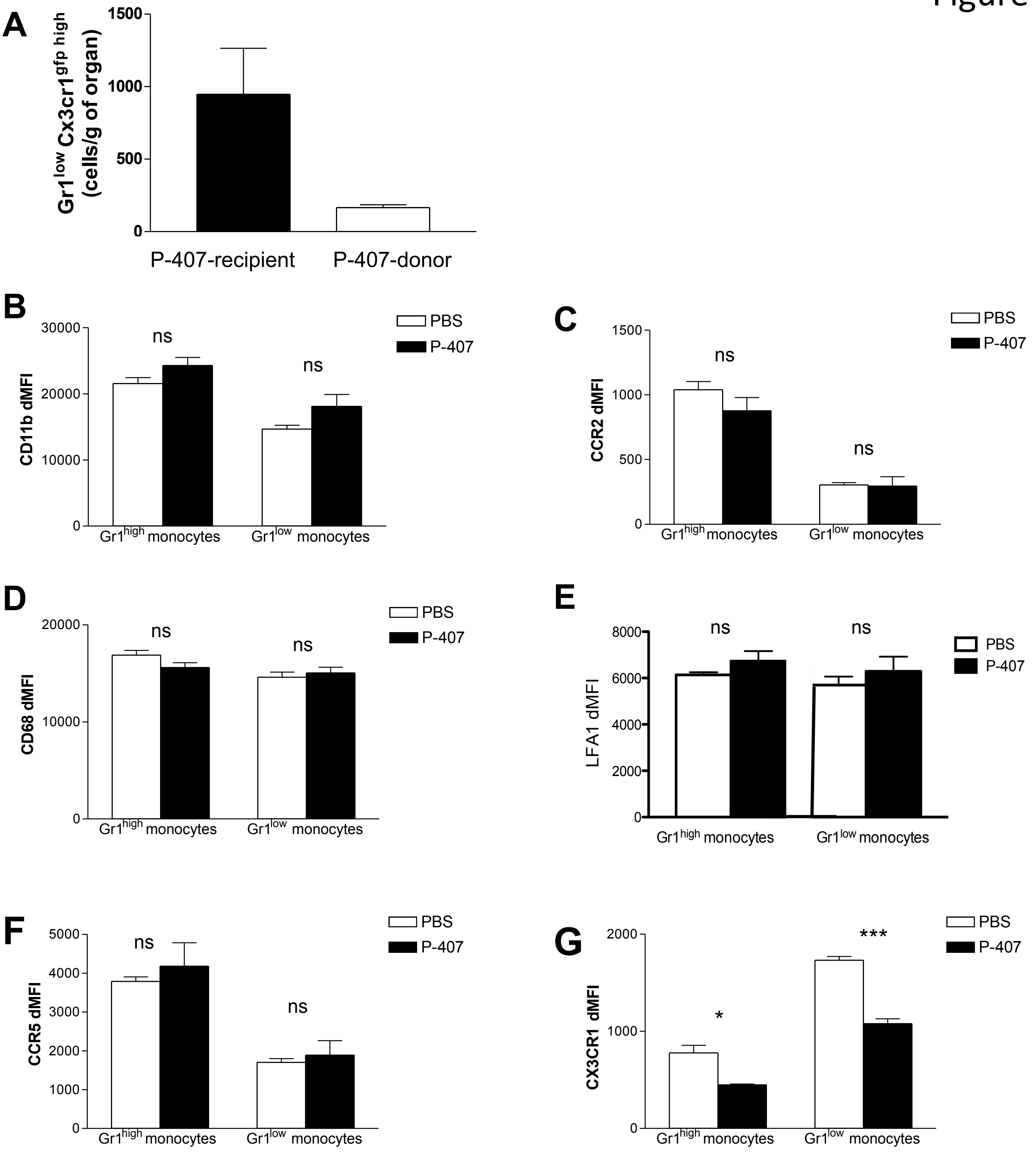
A
Gr1low Cx3cr1gfp high (cells/g of organ)
1500
1000
500
0
P-407-recipient
P-407-donor
B
CD11b dMFI
30000
20000
10000
0
ns
ns
PBS
P-407
Gr1high monocytes
Gr1low monocytes
C
CCR2 dMFI
1500
1000
500
0
ns
ns
PBS
P-407
Gr1high monocytes
Gr1low monocytes
D
CD68 dMFI
20000
10000
0
ns
ns
PBS
P-407
Gr1high monocytes
Gr1low monocytes
E
LFA1 dMFI
8000
6000
4000
2000
0
ns
ns
PBS
P-407
Gr1 high monocytes
Gr1 low monocytes
F
CCR5 dMFI
5000
4000
3000
2000
1000
0
ns
ns
PBS
P-407
Gr1high monocytes
Gr1low monocytes
G
CX3CR1 dMFI
2000
1000
0
*
***
PBS
P-407
Gr1high monocytes
Gr1low monocytes

Figure S6

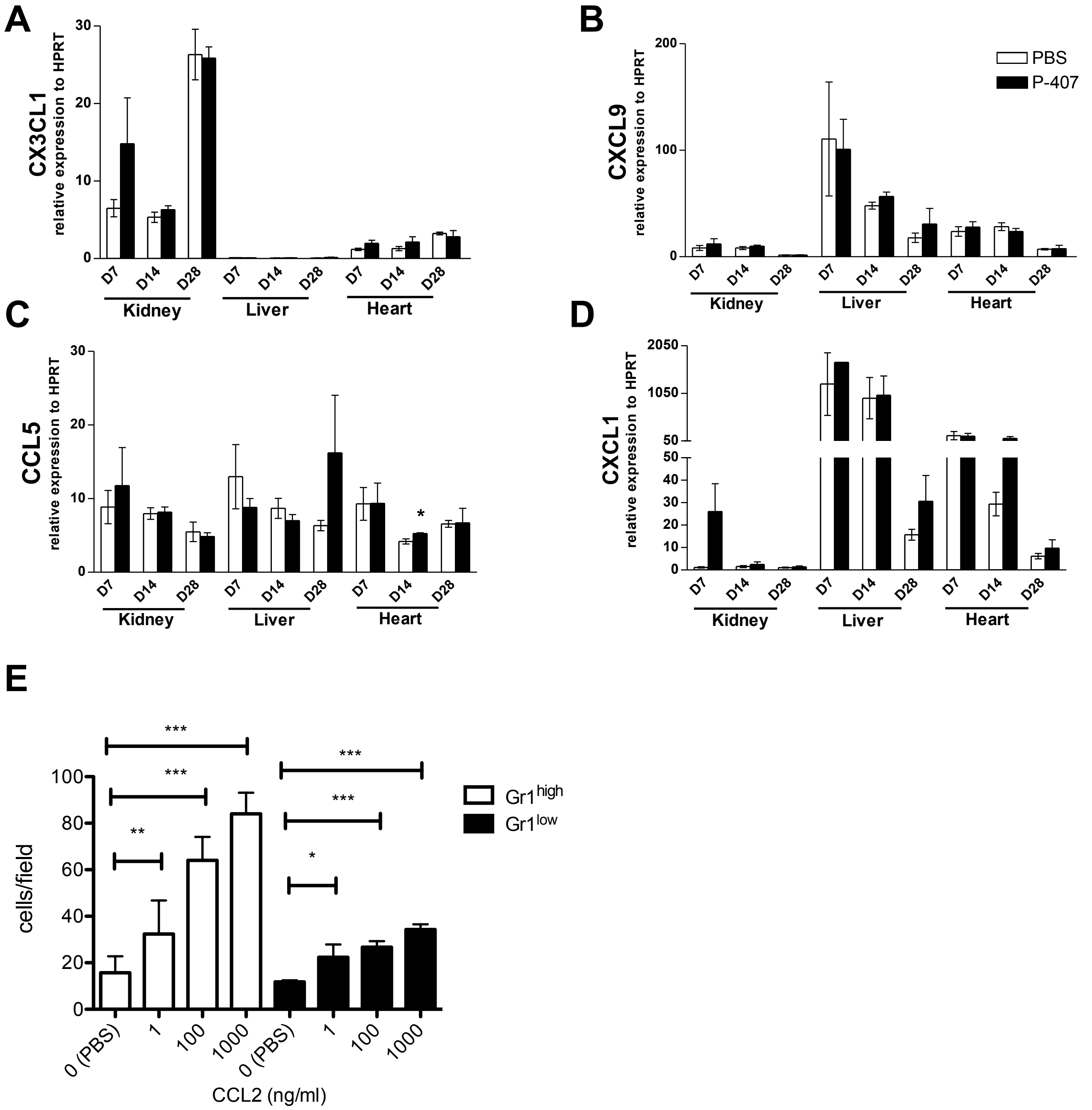

A
CX3CL1
relative expression to HPRT
B
CXCL9
relative expression to HPRT
PBS
P-407
C
CCL5
relative expression to HPRT
D
CXCL1
relative expression to HPRT
D7
D14
D28
Kidney
Liver
Heart
E
cells/field
Gr1high
Gr1low
0 (PBS)
1
100
1000
CCL2 (ng/ml)

Supplemental movies legends

Movie S1, related to Figure 3. Monocyte patrolling in control treated $CX3CR1^{GFP}$ ear dermis. Green is $CX3CR1^{high}$ (GFP+) monocyte/macrophage. Red = 70kDa Dextran. Scale bar = 50µm. Time = Hours:Mins:Secs.

Movie S2, related to Figure 3. Monocyte/macrophage in 7day P-407 (10mg) treated $CX3CR1^{GFP}$ ear dermis. Green is $CX3CR1^{high}$ (GFP+) monocyte/macrophage. Red = 70kDa Dextran. Scale bar = 50µm. Time = Hours:Mins:Secs.

Movie S3, related to Figure 3. Monocyte patrolling in control treated $CX3CR1^{GFP}$ mesentery venule. Green is $CX3CR1^{high}$ (GFP+) monocyte/macrophage. Scale bar = 50µm. Time = Hours:Mins:Secs.

Movie S4, related to Figure 3. Monocyte endothelial accumulation in 7day P-407 (10mg) treated $CX3CR1^{GFP}$ mesentery venule. Green is $CX3CR1^{high}$ (GFP+) monocyte/macrophage. Red = 70kDa Dextran. Scale bar = 50µm. Time = Hours:Mins:Secs.

Supplemental Experimental Procedures

Mice

C57BL/6 and C57BL/6.CD45.1 mice were purchased from Charles River. C57BL/6.LDLR-deficient mice (*Ldlr*$^{-/-}$) and B6.129P-*Cx3cr1*^{tm1Litt}/J (*Cx3cr1*$^{gfp/gfp}$) were purchased from Jackson Laboratory (Bar Harbor, USA). Mice of 8-12 weeks of age (weighing between 18-20g) were used for all experiments. All animals were housed in individually ventilated cages. All procedures were carried out according to the Institutional guidelines for the care and use of experimental animals and the ARRIVE guidelines. Animal studies were approved by the UK Home Office.

Poloxamer 407 administration and lipid levels

Poloxamer 407 (Pluronic F-127, Cat #P2443, Sigma-Aldrich, Dorset, UK) was dissolved overnight in cooled sterile PBS. Mice were injected i.p. with 200μl of P-407 solution (10mg, equivalent to 0.5 g/kg) or PBS every 2nd day. According to the experimental design blood samples were collected into heparin tubes at different time points prior to P-407 injection and 24hrs following drug administration. CHOL and TG levels were measured by a colorimetric assay using the Cholesterol and Triglyceride infinity Reagent (TR13421 & TR22421, respectively, Thermo-Scientific, Middletown, USA). CHOL and TG distribution in plasma lipoprotein fractions was determined on pooled sera by fast performance liquid chromatography gel filtration on a Superose 6 HR 10/30 size-exclusion column. Levels of plasma Apo-E and APOC3 were measured by ELISA (Cusabio, Hubei Province, P.R. China).

In vitro experiments

PBMC from B6 mice were cultured overnight in the presence of P-407 concentration ranging from 50ug/ml to 10mg/ml and then stained for propidium iodide (PI) and Annexin V. PBMC of B6 mice treated with PBS or P-407 for 28 days were stained for neutral lipid using the LipidTox kit (Invitrogen, Paisley, UK).

Bone marrow transplant

Eight week-old female mice were irradiated at 8 Gy using a 137Cs c-ray source and reconstituted with 10^7 bone marrow cells from *Cx3cr1*$^{gfp/gfp}$ mice. Two months later 3 mice were culled and organs (liver, heart, kidney and spleen) were digested at 37^C for 30 min in PBS containing 3% FCS, 1mg/ml collagenase D (Roche), 100U/ml DNaseI (Roche) and 2.4mg/ml Dispase. Cells were analysed by flow cytometry.

Flow cytometry

Peripheral blood was collected from the tail vein or by cardiac puncture under terminal anaesthesia in 5% EDTA tubes and immediately placed on ice. For bone marrow staining, femurs and tibia were harvested and bone marrow flushed. Red cells were lysed using FACS lysing buffer (BD) in accordance to manufacturer's instructions. The following antibodies were used: anti-CD115 (AFS98), anti-Gr1 (RB6-8C5), anti-CD11b (M1-70), anti-CCR5 (HM-CCR5), anti-Ly6C (HK1.4), anti-F4/80 (BM8), anti-CD117 (2B8), anti-LFA1 (M17/4) (all from eBiosciences); anti-CD19 (1D3), anti-CD45.2 (104), anti-CD62L (MEL-14) (all from BD Pharmingen); anti-CCR2 (475301) (R&D systems); anti-CD68 (FA11) and anti-thy1.2 (53-2.1) (Biolegend). Staining was

performed in the presence of a saturating concentration of 2.4G2 monoclonal antibody (anti-CD16/32). In the blood, monocyte subsets were identified as $CD11b^{+}CD115^{+}$ and $Gr1^{high}$ or $CD11b^{+}CD115^{+}$ and $Gr1^{low}$. In the bone marrow cMoPs were identified as $CD117^{+}CD115^{+}Ly6C^{+}CD11b^{-}$ cells, monocytes as $CD117$-$CD115^{+}CD11b^{+}$. Quantification of cell numbers in blood was performed by using AccuCheck counting beads (PCB100, Invitrogen). Flow cytometry was performed with a BD FACSVerse (BD Biosciences, CA, USA). Data were analysed using FlowJo software, version 7.6.5 (TreeStar Inc, Ashland, OR, USA).

Histology and immunohistochemistry

For staining, tissues were fixed in PLP (Periodate-Lysine-Paraformaldehyde) followed by 7% sucrose. Acetone fixed-5µm thick sections were then blocked with 10% milk and 0.03% hydrogen peroxide, sequentially. The sections were stained with anti-CD68 (FA-11, AbD serotec, MCA 1957) and developed using the Polink-2 plus HRP detection kit (GBI Labs, # D46-18). Analysis was performed using a Zeiss microscope. Quantification was carried as previously described (Bhatia et al., 2007). Paraffin sections were stained with H&E or Period Acid-Schiff (PAS) as indicated in the figure legend. For Ki-67 staining, cryostat sections of snap frozen tissue were fixed with acetone and blocked with 20% normal rabbit serum. Sections were sequentially stained with rat mAb against CD68 (Alexa Fluor® 488, FA-11, Biolegend), F4/80 (Biolegend, BM8) and Ki67 (eFluor® 570, SolA15, eBioscience). Coverslips mounted using Vectashield with DAPI (Vector Laboratories). Confocal fluorescence images were acquired on a Leica SP5 confocal microscope with a 40 or 63 x oil immersion objective.

Cell sorting and adoptive transfer experiment

Gr1^{low}GFP^{high} or Gr1^{high}GFP^{low} blood monocytes were sorted from CD45.2*Cx3cr1*$^{gfp/gfp}$ mice using Aria II FACS (Becton-Dickson). 0.1x10^6 Gr1^{low}GFP^{high} or Gr1^{high}GFP^{low} sorted monocytes (>95% purity) were injected i.v. into congenic CD45.1 mice. The same protocol was used to isolated Gr1^{low}GFP^{high} monocytes from CD45.2*Cx3cr1*$^{gfp/gfp}$ mice treated with P-407 for 14 days. At 16hrs mice were injected with 3μg of antibody against CD11b to exclude blood contamination. Mice were perfused with PBS and organs (liver, heart, kidney and spleen) were digested in PBS containing 3% FCS, 1mg/ml collagenase D (Roche), 100U/ml DNaseI (Roche) and 2.4mg/ml Dispase at 37^C for 30 min. The digested cell suspension was then passed through 70um cells strainers. Tissue macrophages from PBS or P-407 treated animals were identified using antibodies against CD45 and F4/80 and sorted using Aria II FACS. Pre-injection of 3μg of antibody against CD115 was used to exclude blood contamination in tissue preparation.

qPCR

RNA was purified using TRIzol reagent (Invitrogen, Paisley, UK) and treated with DNase I (Ambion). RNA was quantified by absorbance spectroscopy (Nanodrop) and cDNA synthesized by reverse-transcriptase reaction (Bio-rad). qRNA analysis was performed using the Real-time PCR Detection System and Brillant II SYBR Green (Agilent technology). mRNA was quantified using a standard curve generated with serial dilutions of input cDNA and normalized using HPRT mRNA concentration. The data are

expressed as relative expression to the HPRT housekeeping gene. Primers are listed below.

	forward primer	reverse primer
CCL2	AGGTGTCCCAAAGAAGCTGTA	ATGTCTGGACCCATTCCTTCT
CCL3	TGCCCTTGCTGTTCTTCTCT	CCCAGGTCTCTTTGGAGTCA
CCL4	GCCCTCTCTCTCCTCTTGCT	GTCTGCCTCTTTTGGTCAGG
CCL5	CCCTCACCATCATCCTCACT	CCCTCACCATCATCCTCACT
CX3CL1	ATTTGTGTACTCTGCTGCC	TCTCCAGGACAATGGCAC
CXCL9	TTTTCCTCTTGGGCATCATC	AGTCCGGATCTAGGCAGGTT
IL-6	TGATGGATGCTACCAAACTGG	TTCATGTACTCCAGGTAGCTATGG
HPRT1	TCAGTCAACGGGGGACATAAA	GGGGCTGTACTGCTTAACCAG

Cytokine levels

Plasma was collected on day 1, day 7 , day 14 and day 28 from mice treated with P-407 or PBS. IL-8, CCL2, IL-6 and TNF-α were measured using a bead multiplex assay (eBioscience) according to the manufacturer's instructions. Bead fluorescence emission was detected using FACSVerse (BD Biosciences) and data analysed using Flow Cytomix (eBiosciences). CCL4 and serum amyloid P componenet (SAP) were measured by ELISA (R&D system and Genway, respectively).

Pertussis Toxin

Mice treated for 10 days with P-407 were injected intravenously with 0.2ug of pertussis toxin (Tocris bioscience) or PBS. One day later monocytes subsets were assessed by FACS analysis.

Splenectomy

Splenectomy was performed and 4 weeks later mice were treated with P-407 or PBS for 2 weeks. Monocytes subsets were then assessed by flow cytometry.

BrdU pulsing

BrdU pulsing was done as described by Yona et al (Yona et al., 2013). Three doses of 2mg BrdU (5-bromo-2-deoxyuridine, BD Pharmingen) were administered i.p. 3 hrs apart. BrdU incorporation in blood monocytes was assessed by bleeding the mice 1, 3, and 5 days following BrdU injection. Monocytes were then stained for BrdU according to manufacturer's instructions (APC BrdU Flow Kit, BD Pharmingen).

Intravital microscopy and image analyses of Ear and Mesentery

Intravital microscopy was performed in mesentery and dermal ear microcirculation of *Cx3cr1*$^{gfp/+}$ and *Cx3cr1* $^{gfp/gfp}$ mice, as previously described (Carlin et al., 2013a). Mice were treated with PBS or P-407 (10mg) for 7 days and under anesthesia mesentery exposed by minimal surgical intervention or ear placed on custom microscopy plate for imaging. Animals were kept warmed and tissue superinfused with warmed fresh saline. For dermal ear imaging, mice were injected with 70kDa Tetramethylrhodamine-dextran (Molecular Probes) to label vasculature. Over 1hr, tissue vasculature was

imaged using Leica SP5 confocal microscope using 20x objective. Imaging exposure was kept minimal to prevent phototoxicity. Areas of interest were analysed using Imaris (Bitplane, version 7.7) and numbers of cells analysed within defined region of interest (ROI). Tracking of cell motility in four dimensions was performed using Imaris. Moving cells were automatically and manually tracked. Track displacement (distance between first and last cell's position) and velocity were calculated from these values.

Transwell migration assay

$Gr1^{low}$ and $Gr1^{high}$ blood monocytes were sorted using Aria II FACS (Becton-Dickson). 1 x 10^5 cells per well were seeded into 3µm-pore transwell inserts (Corning), using a chemoattractant gradient of 1-1000 ng/ml of recombinant mouse CCL4 or CCL2 (R&D systems) or PBS and incubated at 37°C for 2 hours. Non-adherent cells were removed and the transwell inserts were fixed in 4% paraformaldehyde (PFA). Non-migrated cells were removed using a cotton bud before the membranes were excised and mounted on microscope slides in Vectashield mounting media containing DAPI (Vector Labs). Slides were imaged using a 10X objective on an Olympus BX51 widefield fluorescence microscope and nuclei were manually counted from 5 fields.

Statistics

Comparisons between two groups were performed using two-tailed unpaired Student's *t*-test or Mann-Whitney test as indicated in the figure legend. Statistically significant is defined as P < 0.05. N for each experiment is given in the figure or figure legends. *P<0.05; **P<0.01, ***P<0.001

Supplemental References

Bhatia, V.K., Yun, S., Leung, V., Grimsditch, D.C., Benson, G.M., Botto, M.B., Boyle, J.J., and Haskard, D.O. (2007). Complement C1q reduces early atherosclerosis in low-density lipoprotein receptor-deficient mice. Am J Pathol *170*, 416-426.

Yona, S., Kim, K.W., Wolf, Y., Mildner, A., Varol, D., Breker, M., Strauss-Ayali, D., Viukov, S., Guilliams, M., Misharin, A., *et al.* (2013). Fate mapping reveals origins and dynamics of monocytes and tissue macrophages under homeostasis. Immunity *38*, 79-91.
