# Supplementary figures and images for: Triglyceride-Rich Lipoproteins Modulate the Distribution and Extravasation of Ly6C/Gr1low Monocytes
Source: Cell Rep. 2015 Sep 3;12(11):1802–15. doi: 10.1016/j.celrep.2015.08.020 (PMC4590546; doi:10.1016/j.celrep.2015.08.020)

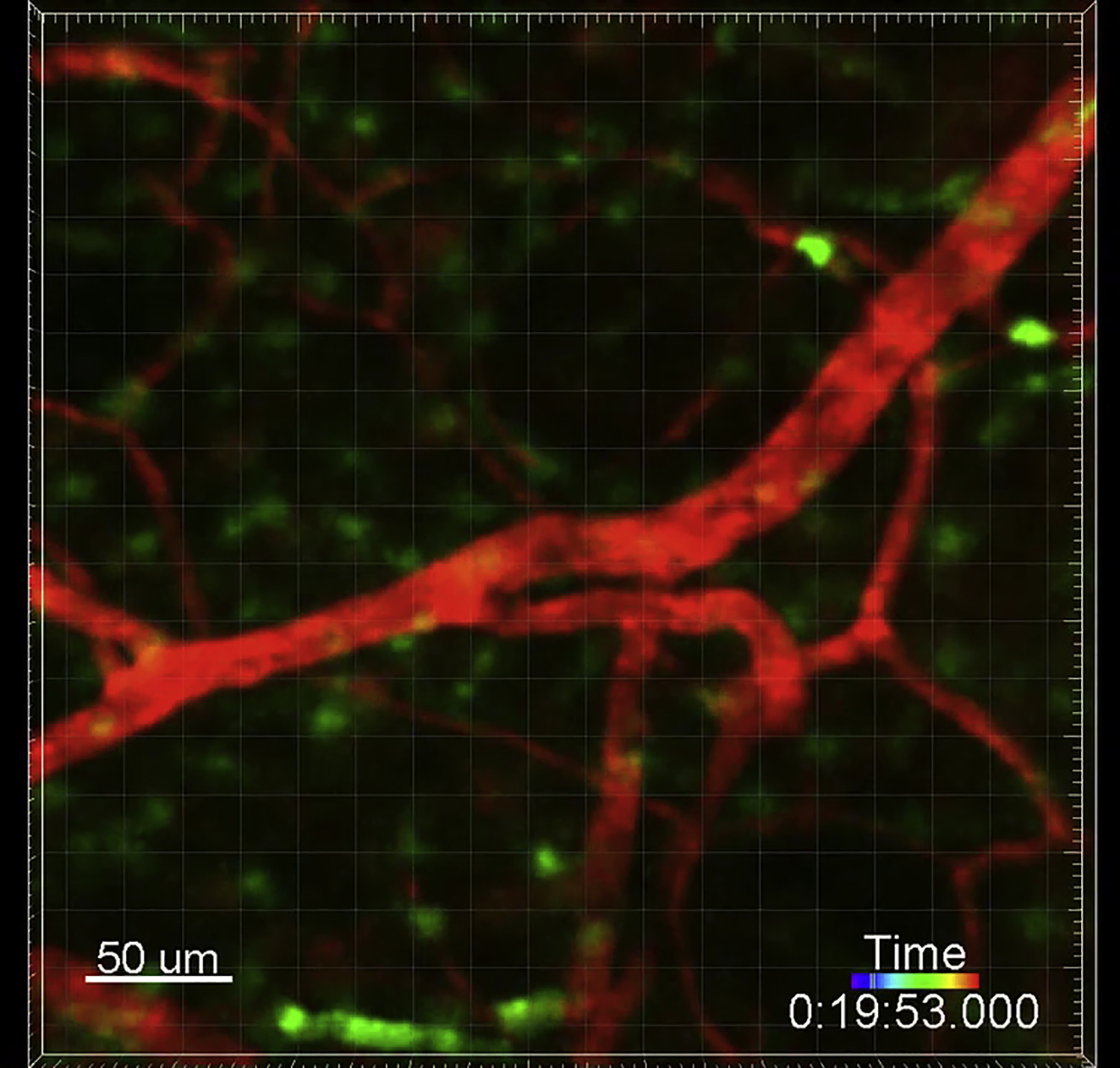

Supplement: Movie S1. Monocyte Patrolling in Control-Treated CX3CR1GFP Ear Dermis, Related to Figure 3 — Green is CX3CR1high (GFP+) monocyte/macrophage. Red, 70 kDa dextran. Scale bar, 50 μm. Time, hr:min:s. [file mmc2.jpg]

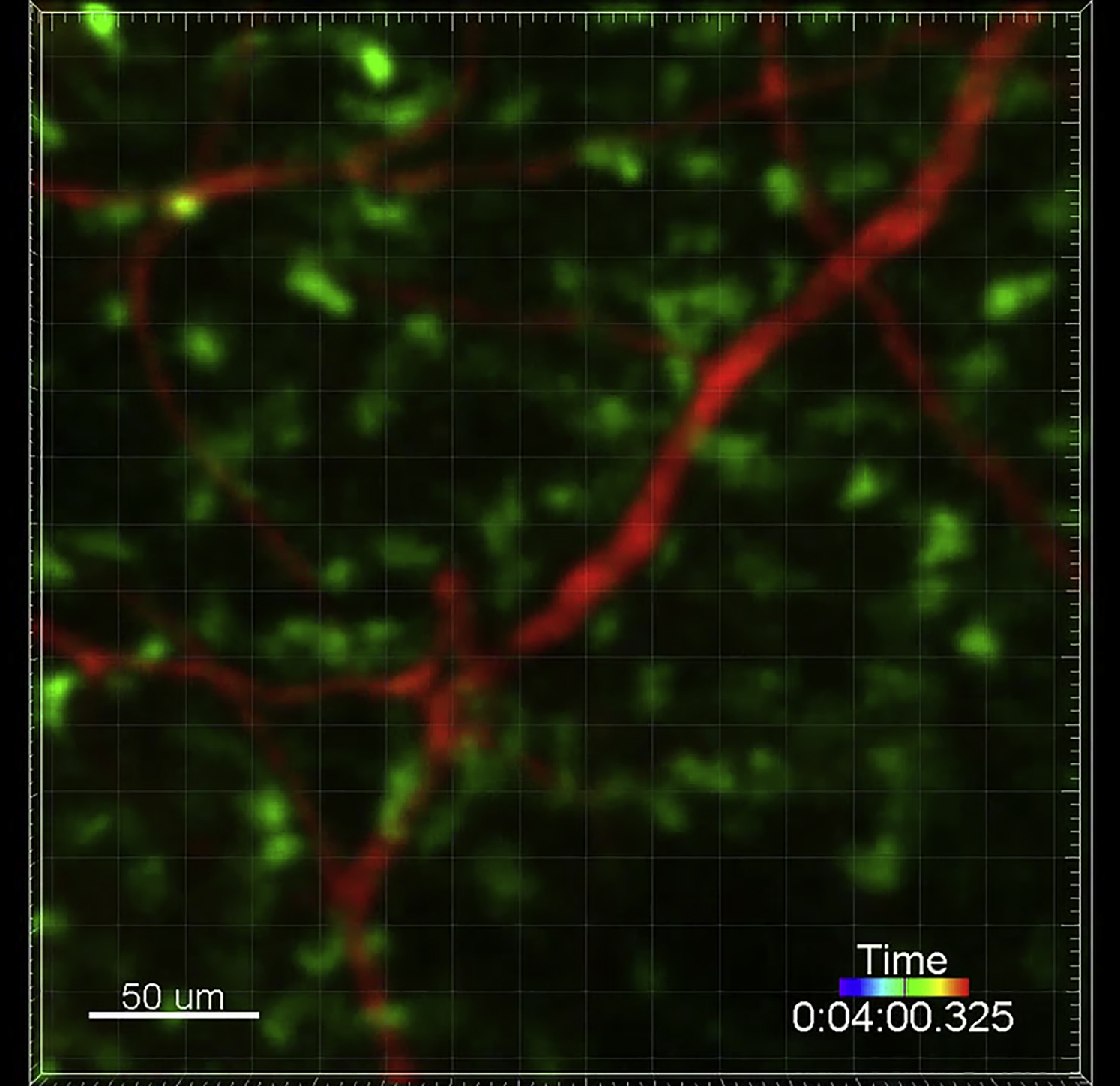

Supplement: Movie S2. Monocyte/Macrophage in 7-Day P-407-Treated CX3CR1GFP Ear Dermis, Related to Figure 3 — Green is CX3CR1high (GFP+) monocyte/macrophage. Red, 70 kDa dextran. Scale bar, 50 μm. Time, hr:min:s. [file mmc3.jpg]

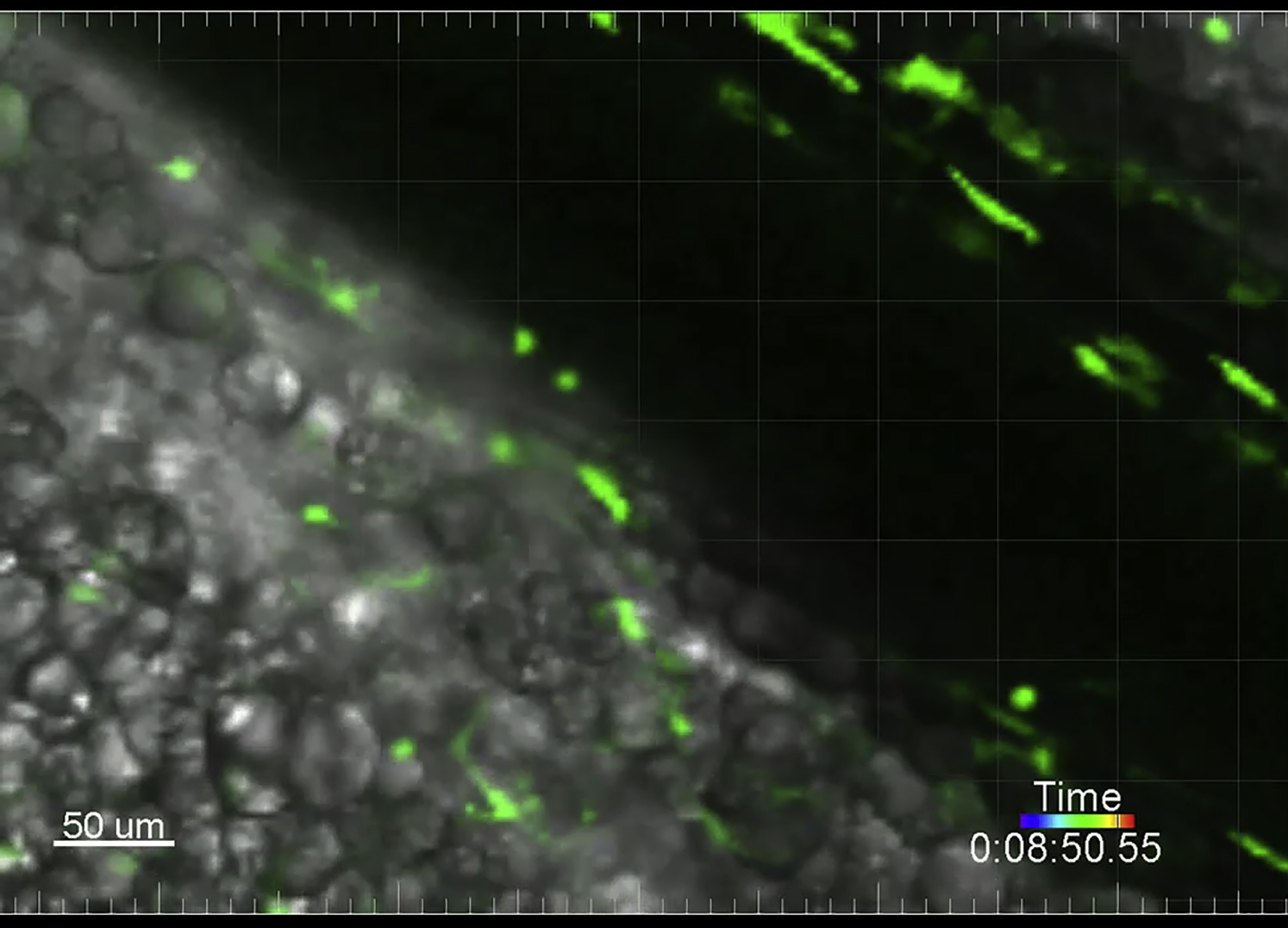

Supplement: Movie S3. Monocyte Patrolling in Control-Treated CX3CR1GFP Mesentery Venule, Related to Figure 3 — Green is CX3CR1high (GFP+) monocyte/macrophage. Scale bar, 50 μm. Time, hr:min:s. [file mmc4.jpg]

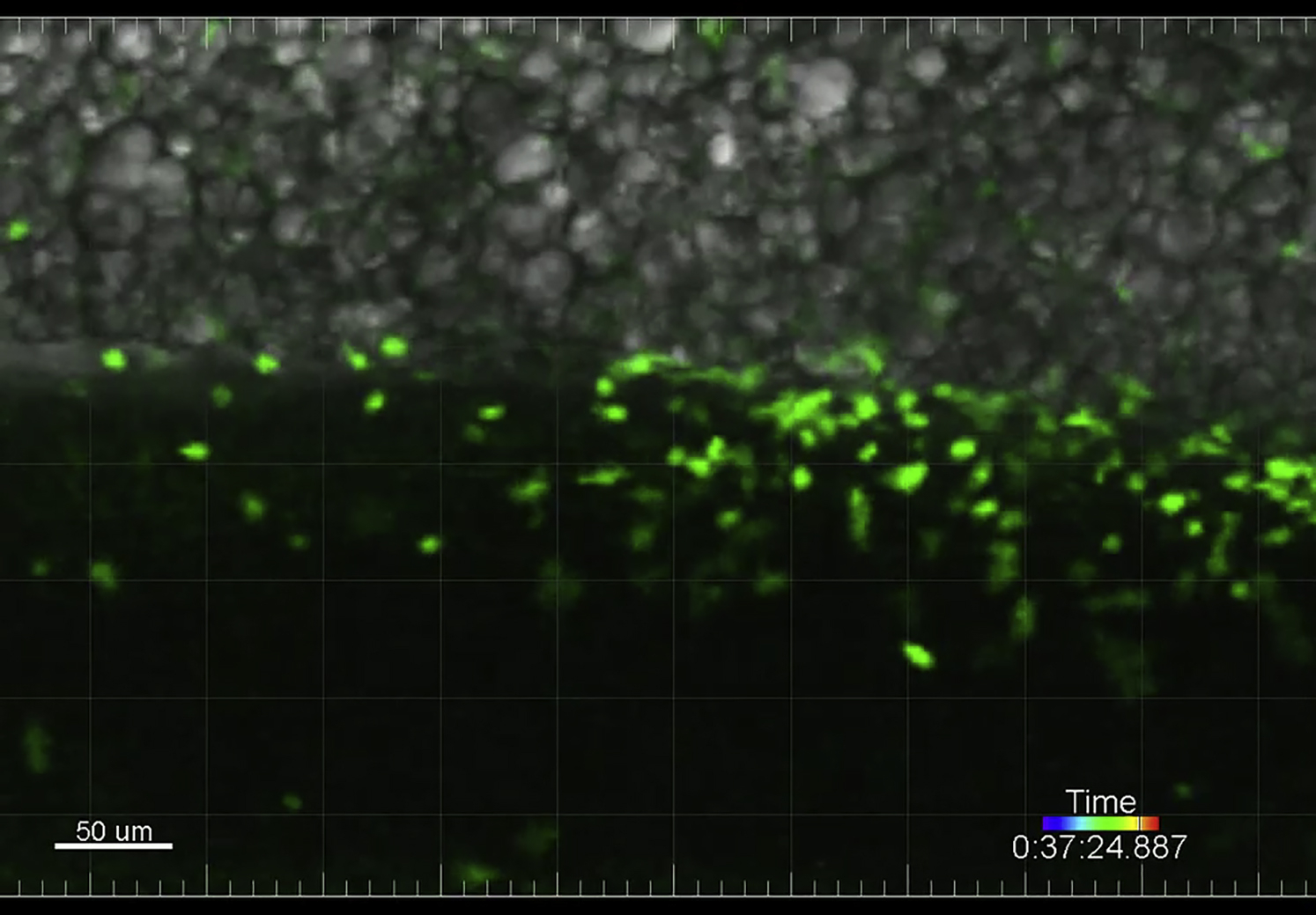

Supplement: Movie S4. Monocyte Endothelial Accumulation in 7-Day P-407-Treated CX3CR1GFP Mesentery Venule, Related to Figure 3 — Green is CX3CR1high (GFP+) monocyte/macrophage. Red, 70 kDa dextran. Scale bar, 50 μm. Time, hr:min:s. [file mmc5.jpg]
