# Supplementary material for: Triglyceride-Rich Lipoproteins Modulate the Distribution and Extravasation of Ly6C/Gr1low Monocytes
Source: Cell Rep. 2015 Sep 3;12(11):1802–15. doi: 10.1016/j.celrep.2015.08.020 (PMC4590546; doi:10.1016/j.celrep.2015.08.020)
Supplement: Document S2. Article plus Supplemental Information [file mmc6.pdf]

Cell Reports

Article

Triglyceride-Rich Lipoproteins Modulate the Distribution and Extravasation of Ly6C/Gr1^{low} Monocytes

Graphical Abstract

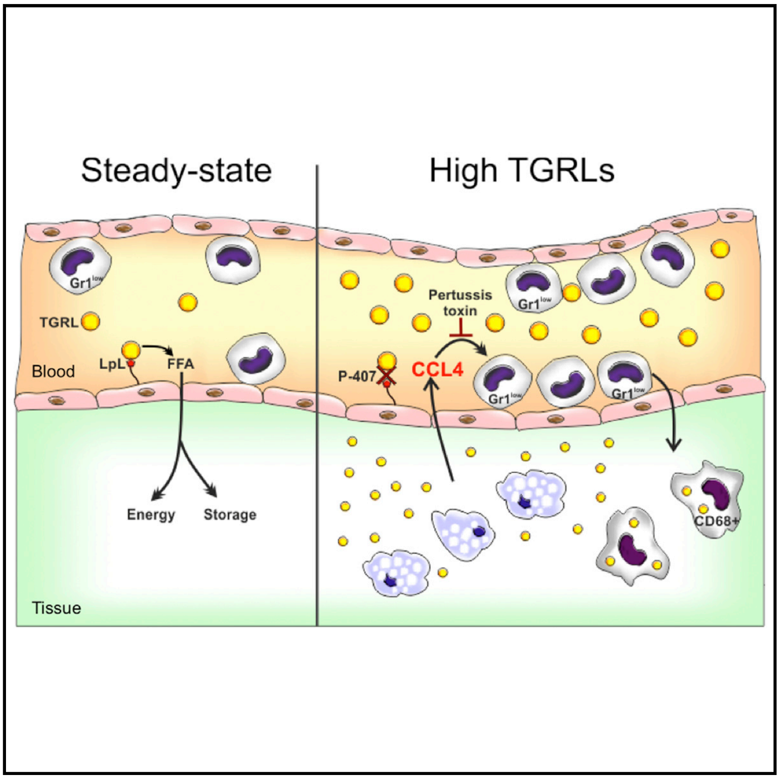

Highlights

- Hypertriglyceridemia promotes the extravasation of Gr1^{low} monocytes
- CD68$^+$ tissue macrophages accumulate in the absence of overt inflammation
- Hypertriglyceridemia is associated with increased CCL4 levels
- CCL4 is involved in the migration of Gr1^{low} monocytes

Authors

Maha F. Saja, Lucie Baudino, William D. Jackson, ..., Matthew C. Pickering, Kevin J. Woollard, Marina Botto

Correspondence

k.woollard@imperial.ac.uk (K.J.W.), m.botto@imperial.ac.uk (M.B.)

In Brief

High levels of triglyceride-rich lipoproteins have been implicated in the pathogenesis of cardiovascular complications. Saja et al. demonstrate that hypertriglyceridemia promotes extravasation of Gr1^{low} monocytes and accumulation of tissue macrophages. Hypertriglyceridemia was associated with increased levels of CCL4, which contributes to the migration of Gr1^{low} monocytes.

CrossMark

Saja et al., 2015, Cell Reports *12*, 1802–1815
September 22, 2015 ©2015 The Authors
http://dx.doi.org/10.1016/j.celrep.2015.08.020

CellPress

OPEN ACCESS
CellPress

Cell Reports
Article

Triglyceride-Rich Lipoproteins Modulate the Distribution and Extravasation of Ly6C/Gr1^{low} Monocytes

Maha F. Saja,[1,3] Lucie Baudino,[1,3] William D. Jackson,[2] H. Terence Cook,[1] Talat H. Malik,[1] Liliane Fossati-Jimack,[1] Marieta Ruseva,[1] Matthew C. Pickering,[1] Kevin J. Woollard,[2,4,*] and Marina Botto[1,4,*]
[1]Centre for Complement and Inflammation Research, Division of Immunology and Inflammation, Department of Medicine, Imperial College London, Du Cane Road, London W12 ONN, UK
[2]Renal and Vascular Inflammation Section, Division of Immunology and Inflammation, Department of Medicine, Imperial College London, Du Cane Road, London W12 ONN, UK
[3]Co-first author
[4]Co-senior author
*Correspondence: k.woollard@imperial.ac.uk (K.J.W.), m.botto@imperial.ac.uk (M.B.)
http://dx.doi.org/10.1016/j.celrep.2015.08.020
This is an open access article under the CC BY license (http://creativecommons.org/licenses/by/4.0/).

SUMMARY

Monocytes are heterogeneous effector cells involved in the maintenance and restoration of tissue integrity. However, their response to hyperlipidemia remains poorly understood. Here, we report that in the presence of elevated levels of triglyceride-rich lipoproteins, induced by administration of poloxamer 407, the blood numbers of non-classical Ly6C/Gr1^{low} monocytes drop, while the number of bone marrow progenitors remains similar. We observed an increased crawling and retention of the Gr1^{low} monocytes at the endothelial interface and a marked accumulation of CD68^{+} macrophages in several organs. Hypertriglyceridemia was accompanied by an increased expression of tissue, and plasma CCL4 and blood Gr1^{low} monocyte depletion involved a pertussis-toxin-sensitive receptor axis. Collectively, these findings demonstrate that a triglyceride-rich environment can alter blood monocyte distribution, promoting the extravasation of Gr1^{low} cells. The behavior of these cells in response to dyslipidemia highlights the significant impact that high levels of triglyceride-rich lipoproteins may have on innate immune cells.

INTRODUCTION

Marked elevations in triglyceride-rich lipoprotein (TGRL) levels are observed in individuals with rare genetic disorders such as familial lipoprotein lipase deficiency (Benlian et al., 1996) and when a common genetic disorder occurs in association with an acquired secondary form of hypertriglyceridemia such as diabetes or alcohol consumption (Pejic and Lee, 2006). Furthermore, abnormalities in TGRL levels are commonly observed in patients with persistent infections like HIV (Oh and Hegele, 2007) or chronic inflammatory conditions such as systemic lupus erythematosus (SLE) (Bruce, 2005). Although elevated TGRL levels are thought to contribute to the increased risk of cardiovascular complications observed in all these conditions (Benlian et al., 1996; Bruce, 2005), the pathogenic impact of an abnormal TGRL profile remains poorly understood.

The mononuclear phagocyte system (MPS) plays a central role in the maintenance of tissue integrity. In a hyperlipidemic environment, cells of the MPS ingest excess lipids that activate them through a variety of signaling pathways, leading to increased secretion of pro-inflammatory cytokines and eventually cell death (Moore and Tabas, 2011). The notion of toxic and inflammatory effects of lipid metabolites has been supported by an extensive literature using murine models of hyperlipidemia such as strains genetically deficient in either the low-density lipoprotein receptor (LDLR) or apolipoprotein E (ApoE). These models replicate human hypercholesterolemic states and the associated inflammatory response, but they do not recapitulate triglyceride-rich dyslipidemia. Moreover, the nature of the lipid responsible for the MPS responses in hyperlipidemia remains unresolved; both inflammatory and anti-inflammatory effects have been attributed to cholesterol (Spann and Glass, 2013). Finally, the MPS cell type responding to hyperlipidemia remains poorly understood. Most work has focused on dendritic cells and tissue macrophages, while much less attention has been given to blood monocytes. However, reports of postprandial activation of monocytes from acute changes in TGRLs (Gower et al., 2011) suggest that these cells may be important in the handling of circulating lipids.

Monocytes, identified as CD11b^{+}CD115^{+} cells, are a heterogeneous population of blood leukocytes with phagocytic and immunomodulatory properties. At least two phenotypically and functionally distinct monocyte subsets have been described in humans, rats, pigs, and mice (Geissmann et al., 2003; Yona and Jung, 2010; Ziegler-Heitbrock, 2014), indicating evolutionary conservation. The murine monocyte subpopulations have been termed "classical" and "non-classical" based on

CrossMark

differences in surface markers and functional properties. The classical monocytes express lower levels of CX3CR1 and higher levels of the C-C chemokine receptor 2 (CCR2) and lymphocyte antigen 6c (Ly6C) (or the myeloid differentiation antigen Gr1) and are defined as CX3CR1^{int}CCR2^{high}Gr1^{high} (abbreviated as Gr1^{high}). They are considered to be equivalent to CD14^{high} human monocytes (Cros et al., 2010). The Gr1^{high} cells represent the inflammatory monocyte subtype and are actively recruited to inflamed tissue, where they may give rise to macrophages. Their behavior appears to be distinct from the second blood monocyte subpopulation, identified as CX3CR1^{high}CCR2^{low} Gr1^{low} (abbreviated as Gr1^{low}). The equivalent cells in humans are identified as CD14^{low}CD16^{high} (Cros et al., 2010). The Gr1^{low} monocytes have been shown to patrol the luminal surface of endothelial cells, acting as "housekeepers" of the vasculature (Auffray et al., 2007; Carlin et al., 2013b). Their migratory properties remain elusive, and the possibility they may sense and respond to different environmental stimuli in the absence of inflammation is unexplored.

Given the fact that in steady-state conditions, monocytes do not contribute to the maintenance of most peripheral tissue macrophages (Hashimoto et al., 2013; Yona et al., 2013) but are exposed to changes in plasma lipids, their behavior during hyperlipidemia requires investigation. Here, we report that a hyper-TGRL environment promotes differential migration of blood monocytes. Hyper-TGRL induced Gr1^{low} monocytes to extravasate into surrounding tissue. This process was associated with increased CCL4 levels and was partially dependent on a pertussis toxin (PT)-sensitive receptor axis. Our findings indicate that Gr1^{low} monocytes, in addition to patrolling the endothelial luminal surface (Auffray et al., 2007; Carlin et al., 2013b), are likely to have an extravascular role under hyper-TGRL conditions.

RESULTS

Elevated TGRL Levels Alter Blood Monocyte Subset Distribution

To mimic the increased TGRL levels observed in many patients with metabolic disorders, we used the poloxamer 407 (P-407)-induced model of dyslipidemia (Johnston, 2004). This chemically induced model of hyperlipidemia involves the intraperitoneal (i.p.) administration of the non-ionic surfactant P-407 to wild-type C57BL/6 (B6) mice on chow diet and allows for a dose-controlled dyslipidemia. We first established the dosing regimen that resulted in a stable increase in TGRL levels. As previously reported (Johnston, 2004), we found that administration of P-407 (0.5 g/kg every second day) to B6 mice for 28 days resulted in an average plasma triglyceride (TG) concentration of 2,691.2 ± 501.8 mg/dl compared with 80.2 ± 6.26 mg/dl in PBS-treated controls and a modest increase in plasma cholesterol (CHOL) (324 ± 37.1 mg/dl in P-407-treated mice versus 106.2 ± 3.27 mg/dl in PBS-treated mice [mean ± SEM]) (Figures S1A–S1D). Chromatography showed that in P-407-treated animals, all of the TG was associated with the VLDL fraction (Figures S1E and S1F), consisting mainly of Apo-E and apolipoprotein C-III (APOC3) (Figure S1G). Importantly, the P-407-induced dyslipidemia did not trigger an overt inflammatory response (Figure S1H). The body weight of the P-407-injected animals did not change (Figure S1I) and no evidence of renal damage was detected as judged by the absence of hematuria and albuminuria (data not shown). As previously reported (Johnston et al., 1993), there was a mild splenomegaly in the P-407-treated group when compared to controls (Figure S1J). Histological analysis after 4-week treatment showed some cells with a characteristic foamy cytoplasm in the spleen, liver, and heart (Figure S1K), indicating increased tissue lipid load and foam cell formation.

Diet-induced hypercholesterolemia in ApoE-deficient mice has been shown to induce monocytosis associated mainly with a shift toward an increased frequency of the Gr1^{high} subset compared with Gr1^{low} fraction (Swirski et al., 2007; Tacke et al., 2007). The increase in TGRLs induced by P-407 treatment did not alter the total number of blood monocytes (Figure 1A), but the distribution of the two major monocyte subsets showed a different pattern (Figure 1B). We observed a progressive and marked drop in the numbers of Gr1^{low} monocytes starting as early as 7 days post-P-407 injections with a relatively modest increase in the number of Gr1^{high} monocytes (Figures 1C and 1D). At the end point, the P-407-treated mice had dropped their Gr1^{low} monocyte numbers to less than half compared to the starting point ($p < 0.0001$), while the number of Gr1^{high} monocytes was not significantly different ($p = 0.25$). In contrast, other peripheral blood cells (total white cell, B and T cells, and polymorphonuclear leukocytes) did not show consistent or reproducible changes in their numbers or frequencies (Figures S2A–S2H). The drop in circulating Gr1^{low} monocytes under the P-407 treatment could not be explained by a selective toxic effect of the dyslipidemia as the percentages of the Annexin V^{+}/PI^{+} monocytes were not markedly different between the two monocyte subpopulations, irrespective of the treatment (Figure 1E). Additionally, in vitro exposure of peripheral blood mononuclear cells (PBMCs) to P-407 concentrations ranging from 50 μg/ml to 10 mg/ml did not affect the survival of the cells (Figure S2I). We then investigated a model of diet-dependent hyperlipidemia using the LDLR-deficient (*Ldlr*$^{-/-}$) mice that develop hypercholesterolemia and a modest hypertriglyceridemia on a high-fat (HF) diet (Figures S2J and S2K). Animals were euthanized after 50 days on the diet, an age at which *Ldlr*$^{-/-}$ mice do not develop advanced atherosclerotic lesions (Ma et al., 2012) or monocytosis (Murphy et al., 2011) (Figure S2L). Under these conditions, we observed significantly fewer Gr1^{low} monocytes in the *Ldlr*$^{-/-}$ mice kept on an HF diet compared to those maintained on a chow diet (Figures S2M and S2N), indicating that the changes in monocyte subsets in P-407-treated animals were the result of the dyslipidemia and not an adverse effect of P-407.

Consistent with the inhibitory effect of P-407 on the capillary endothelial lipoprotein lipase (Johnston, 2004), a known mediator of TG hydrolysis, we also found that P-407 did not induce accumulation of neutral lipid content in leukocytes (Figures 1F and 1G). On the contrary, there was a trend toward a decreased amount of lipid in the blood cells of the P-407-treated animals. Taken together these data indicate that the increase in TGRLs modulates the steady-state distribution of the blood monocyte subsets without altering other leukocytes. We then explored whether the changes triggered by the P-407 treatment occurred in the bone marrow (BM) or in the periphery.

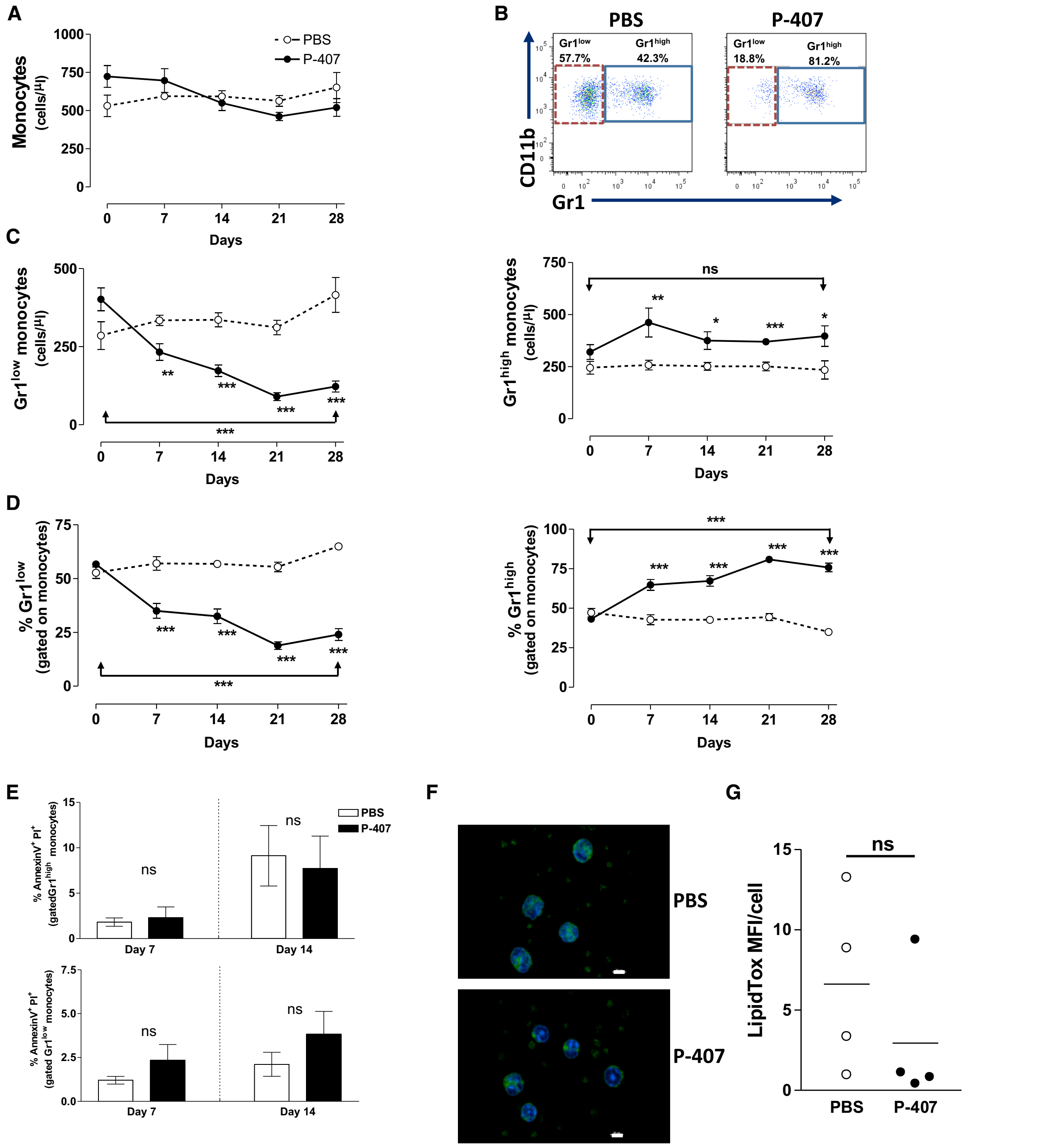

Figure 1. Characterization of Blood Monocytes in P-407-Treated Mice

(A) Quantitative analysis of total monocyte numbers in B6 mice treated with P-407 or PBS for 28 days. Circulating monocytes were identified by their low SSC and co-expression of CD115 and CD11b.

(B) Representative dot plots showing the frequencies of Gr1^{high} and Gr1^{low} monocyte subsets at the end of the time course.

(C and D) Numbers (C) and relative frequencies (D) of Gr1^{low} and Gr1^{high} blood monocytes. P-407-treated mice kept on a chow diet had significantly fewer Gr1^{low} and more Gr1^{high} monocytes at each time point. Plots represent data pooled from four different experiments and show the mean values ± SE for at least seven mice per time point. The p value of the comparison between day 0 and day 28 is indicated (*p < 0.05; **p < 0.01, and ***p < 0.001; unpaired t test).

(E) Frequency of Annexin V^{+}/PI^{+} monocytes in the two monocyte subpopulations 7 and 14 days after P-407 treatment. Results are expressed as mean ± SE, n = 4 per time point (unpaired t test).

(legend continued on next page)

The P-407-Induced Dyslipidemic Environment Does Not Affect the Generation of Gr1^{low} Monocytes

We first examined the frequency of the BM-resident founder cells termed common monocyte progenitor (cMoP) (Hettinger et al., 2013). The analysis of the BM at 2 and 4 weeks failed to show any significant difference between the two experimental groups in the numbers of cMoPs, total monocytes, and monocyte subpopulations (Figures 2A–2D), demonstrating that the drop in Gr1^{low} monocytes could not be the result of an impaired production in the BM. As the spleen can contribute to the clearance of apoptotic monocytes (Getts et al., 2014) and at the same time act as a "reservoir" for the Gr1^{high} monocytes (Swirski et al., 2009), we assessed the effect of splenectomy on the monocyte distribution. Splenectomized mice treated with P-407 still had a drop in the percentage and number of Gr1^{low} monocytes when compared to the PBS-treated group (Figures 2E and S3A), confirming that P-407 did not promote the splenic clearance of apoptotic Gr1^{low} monocytes (see also Figure 1E).

Gr1^{high} monocytes have been shown to be the precursors of the steady-state Gr1^{low} cells (Tacke et al., 2006; Yona et al., 2013). Therefore, we used a 5-bromo-2′-deoxyuridine (BrdU) pulsing regimen to investigate whether the elevated TGRLs prevented Gr1^{high} > Gr1^{low} conversion or increased the half-life of Gr1^{high} blood monocytes. Mice were pulsed with three i.p. injections of 2 mg BrdU (3 hr apart), and treatment with P-407 or PBS was started on the same day. As shown in Figures 2F and S3B, the BrdU incorporation into the two monocyte subsets was tracked over a period of 5 days. By day 1, the vast majority of BrdU$^+$ monocytes were Gr1^{high} (Figure 2F, left panel). This continued for 3 days when a significant drop of the BrdU$^+$ monocytes was noted in both experimental groups, indicating that the dyslipidemic environment had not altered the half-life of the Gr1^{high} subset and thus this could not be the explanation for the drop in the Gr1^{low} compartment. A small fraction of the Gr1^{low} subset showed BrdU staining by day 1, and this gradually increased over 5 days in the PBS-treated group (Figure 2F, right panel), confirming the delayed BrdU incorporation into the Gr1^{low} compartment (Yona et al., 2013). The kinetic of the BrdU incorporation in the Gr1^{low} fraction differed slightly in the P-407-treated mice and the expected increase at day 5 was not observed (Figure 2F, right panel), suggesting the possibility of a different steady-state behavior of this monocyte compartment in the dyslipidemic environment. To investigate this, we studied effects of elevated TGRLs on the mobility of Gr1^{low} monocytes in vivo.

Increased TGRL Levels Promote CX3CR1^{high} Monocyte Crawling and Tissue Accumulation of CD68$^+$ Macrophages

Gr1^{low} monocytes are thought to monitor endothelial integrity (Auffray et al., 2007; Carlin et al., 2013b). To examine the effect of the P-407-induced hyper-TGRL environment on the Gr1^{low} monocytes at the endothelial interface, we used the Cx3cr1^{gfp} reporter mouse strain. This has been widely used to discriminate the two monocyte subpopulations (Geissmann et al., 2003) and enables Gr1^{low}CX3CR1^{high} monocytes to be tracked in situ by intravital microscopy (Auffray et al., 2007; Cros et al., 2010). Analysis of monocyte behavior in vivo showed that the number of intravascular crawling GFP$^+$ cells per hour in the ear dermis was higher in the mice treated with P-407 for 7 days compared to the PBS counterparts (Figures 3A and 3B; Movies S1 and S2). Analysis of the intravital images revealed an increased accumulation of extravascular GFP$^+$ cells with a migratory behavior in the P-407-treated animals (Figures 3A and S4A). We then performed in situ experiments in the mesentery of *Cx3cr1*$^{gfp/gfp}$ and *Cx3cr1*$^{gfp/+}$ mice (Figures 3C–3E) and found a marked accumulation of GFP$^+$ cells at the endothelial interface over 1 hr in P-407-treated *Cx3cr1*$^{gfp/+}$ and *Cx3cr1*$^{gfp/gfp}$ mice (Figure 3C; Movies S3 and S4). This was accompanied by decreased velocity and track displacement (Figure 3C), both indicators of enhanced dwell time and endothelial retention. In addition, we observed again a significant increase in tissue (extravascular) GFP$^+$ cells with a migratory behavior (Figures 3D, 3E, and S4B). Since there was no difference between *Cx3cr1*$^{gfp/+}$ and *Cx3cr1*$^{gfp/gfp}$ mice, this implies that under our hyper-TGRL conditions, the Gr1^{low} monocytes had acquired the ability to extravasate independently of CX3CL1 signaling.

To extend the intravital microscopy findings to other organs, we then stained liver, heart, and kidney with an antibody against CD68, a widely used marker for tissue macrophages. The immunohistochemical quantification at different time points revealed a striking and gradually increasing accumulation of CD68$^+$ cells in the P-407-injected animals compared to the PBS-treated controls (Figures 4A and 4B). F4/80 staining of the liver confirmed the increased presence of macrophages (Figure S4C). In agreement with the intravital findings, the number of CD68$^+$ cells was already significantly increased 1 week after P-407 injections.

As recent papers have highlighted the self-renewal properties of tissue macrophages (Epelman et al., 2014), we used Ki-67, a well-known marker of proliferation, and found no evidence of proliferation (Figure 4C). Collectively, these data indicated that the drop in Gr1^{low} blood monocytes under hyper-TGRL conditions could be due to increased extravasation and tissue accumulation.

Gr1^{low} Monocytes Are Recruited into Tissues under Increased TGRL Conditions

To formally demonstrate that Gr1^{low} monocytes were indeed trafficking into the organs in the P-407-treated mice, we performed BM transplant and adoptive transfer experiments using the *Cx3cr1*$^{gfp/gfp}$ mice. Two months after the engraftment of the *Cx3cr1*$^{gfp/gfp}$ BM cells into B6 recipients, we detected a large number of GFP$^+$ cells in all the organs prior to any P-407 intervention (Figure S4D), probably as a result of the damage caused

(F) Representative images of neutral lipid content in blood leukocytes after 28 days of PBS or P-407 treatment. Leukocytes were stained for neutral lipid accumulation using the LipidTox kit according to manufacturer's instructions. Neutral lipid droplets are shown in green. Scale bar, 10 μm.
(G) Quantification of the lipid content. Data are expressed as mean fluorescent intensity (MFI) per cell (mean of three fields per mouse, 15 cells/field, and four mice per group; unpaired t test). Horizontal bars represent means. ns, nonsignificant.
See also Figures S1 and S2.

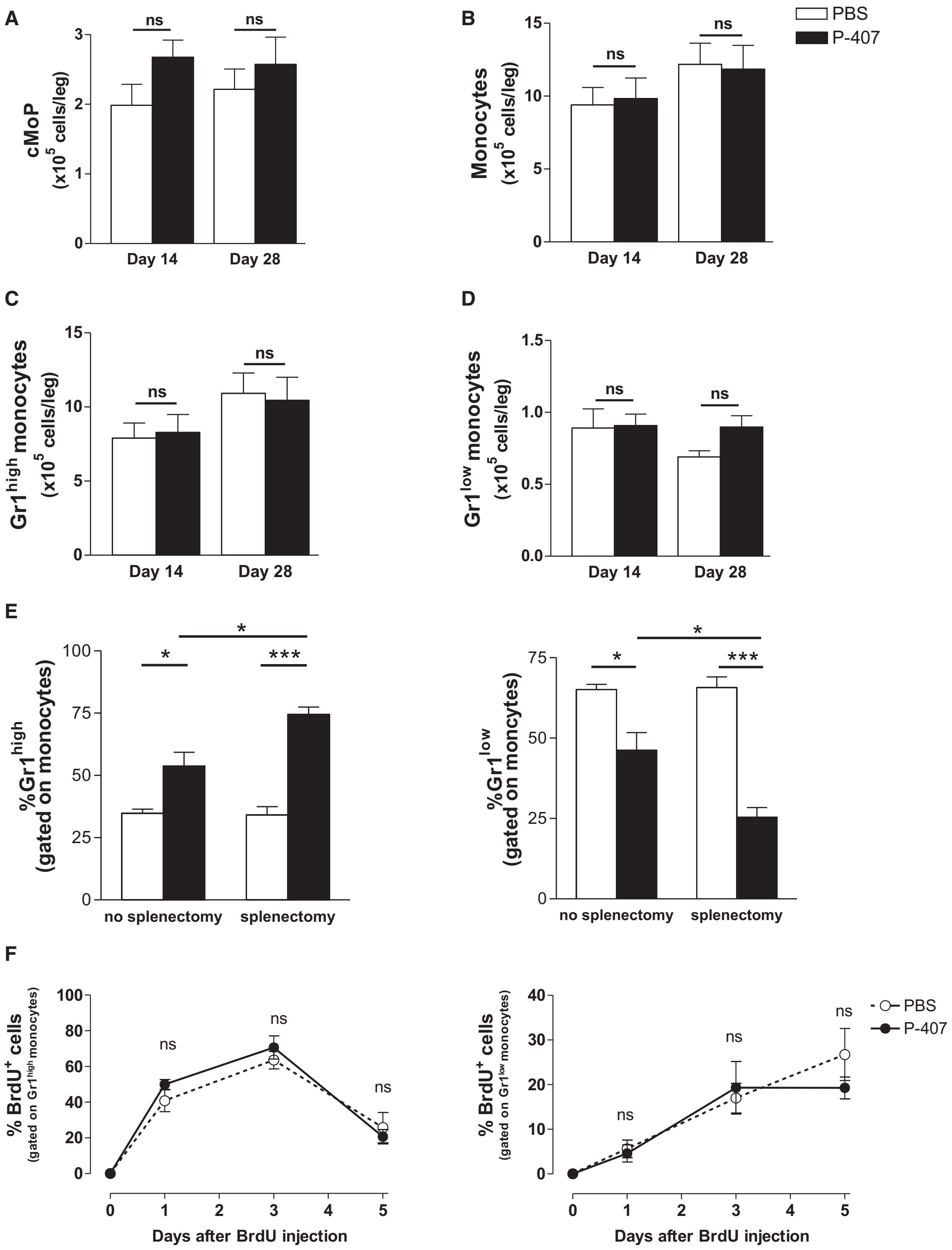

Figure 2. Modulation of BM and Blood Monocyte Dynamics in P-407-Treated Mice

(A–D) Quantitative analysis of cMoPs (A), monocytes (B), and Gr1^{high} (C) and Gr1^{low} monocytes (D) in the BM after 14 and 28 days of P-407 or PBS treatment. Results are expressed as mean ± SE, n = 4 per time point. p values are indicated (unpaired t test); ns, nonsignificant.

(E) Frequencies of Gr1^{high} and Gr1^{low} monocyte subsets after splenectomy in mice treated with PBS or P-407 for 14 days. Results are expressed as mean ± SE, n = 4. *p < 0.05 and ***p < 0.001 (unpaired t test).

(legend continued on next page)

by irradiation and repopulation of tissue resident macrophages from BM progenitors (Hashimoto et al., 2013), and thus, we abandoned this approach. We next adoptively transferred Gr1^{low}GFP^{high} or Gr1^{high}GFP^{low} monocytes, isolated from CD45.2*Cx3cr1*$^{gfp/gfp}$ mice, into CD45.1B6 animals pretreated with either P-407 or PBS for 2 weeks. Recipients were sacrificed 16 hr after the adoptive transfer. To exclude the contamination from circulating monocytes, we also injected an anti-CD11b antibody that labeled blood monocytes immediately prior to the perfusion of the organs. Adoptively transferred Gr1^{low}GFP^{high} monocytes were detected in the liver, spleen, and kidney of P-407-treated animals to a much greater extent than in the PBS-treated recipients (Figures 5A and 5B). There was no obvious recruitment in the heart, possible because of different kinetics of macrophage accumulation (Figure 5B). Of note, we found no increase of Gr1^{high}GFP^{low} monocytes in the P-407-treated organs (Figures 5A and 5B), corroborating our earlier assumption that it was mainly the Gr1^{low} subpopulation that had extravasated in the tissues in response to the hyper-TGRL environment. Furthermore, Gr1^{low}GFP^{high} monocytes isolated from P-407-treated CD45.2*Cx3cr1*$^{gfp/gfp}$ mice did not extravasate when adoptively transferred into PBS-treated recipients (Figure S5A). The monocyte expression of CD11b, CCR2, CD68, LFA-1, and CCR5 (Figures S5B–S5F) did not change in response to the P-407 treatment, demonstrating that neither the P-407-induced hyper-TGRL environment nor the compound itself had altered the phenotype of the monocytes. To determine if the TGRL-induced drop in the number of Gr1^{low} monocytes was a reversible process, we treated mice with P-407 for 2 weeks and then stopped the treatment. On stopping the P-407 administration, lipid levels returned to normal by 72 hr (see Figure S1) and the Gr1^{low} fraction returned to pre-treatment levels by 2 weeks (Figure 5C).

To investigate the mechanism(s) of the extravasation we quantified tissue mRNA expression of a large panel of chemokines/cytokines. As shown in Figure 6, this analysis showed a marked upregulation of CCL4 in the organs from the P-407-treated animals compared to those from the PBS controls. CCL2 and CCL3 also showed some increased expression. Of note, IL-6 mRNA expression showed a very different pattern and was reduced in the liver from P-407-treated animals (Figure 6D), confirming that the P-407 treatment did not elicit an overt inflammatory response. In keeping with the intravital data, the expression of CX3CL1 (fractalkine) was not affected by the P-407 treatment (Figure S6A). Similarly, other chemokines such as CXCL9, CXCL1, and CCL5 were expressed at similar levels in the two experimental groups (Figures S6B–S6D). As macrophages can produce a large amount of CCL4 (Maurer and von Stebut, 2004) and were increased in tissues following P-407 treatment, we hypothesized that they could represent a potential source of the CCL4. We therefore analyzed CCL4 mRNA expression in sorted CD45^{+}F4/80^{+} tissue macrophages after 2 weeks of PBS or P-407 treatment. We found that macrophages from heart and liver of P-407-treated animals had a more than 2-fold increase in CCL4 expression compared to those from PBS-treated controls (Figure 6E). We observed only a slight increase in CCL2 expression in the liver macrophages from P-407-treated mice (Figure 6E). We then measured CCL2 and CCL4 plasma levels. In keeping with the gene expression data, we detected CCL4 only in the animals treated with P-407 and not in the PBS controls (Figure 6F), demonstrating that a hyper-TGRL environment could induce the production of this chemokine. We also detected an increase in CCL2 levels in the P-407 mice, but the increase reached statistical significance only at day 14 (Figure 6F).

CCR5, which binds CCL4 and CCL3, was not selectively expressed in Gr1^{low} monocytes, regardless of the lipid levels (Figure S5F), making it an improbable candidate. Moreover, CX3CR1 expression in both monocyte subsets was lower following P-407 treatment, confirming that CX3CL1 signaling was not driving the extravasation (Figure S5G). To support the idea that a chemokine/chemokine receptor axis may contribute to the migration of Gr1^{low} monocytes into the peripheral tissue in P-407-treated mice, we assessed whether the drop in the Gr1^{low} compartment could be rectified by PT, a potent inhibitor of Gα_i-coupled receptor signaling, including chemokine receptors. A single administration of PT after 10 days of P-407 treatment prevented further extravasation of the Gr1^{low} monocytes, and the percentage of Gr1^{low} monocytes increased to levels similar to those in PBS-treated mice (Figure 6G). Additionally, transwell chemotaxis assays with fluorescence-activated cell-sorted Gr1^{low} or Gr1^{high} monocytes and recombinant mouse CCL4 confirmed that CCL4 preferentially induces migration of Gr1^{low} monocytes (Figure 6H), while CCL2 induces mainly migration of Gr1^{high} (Figure S6E). Collectively, these findings suggest that elevated levels of TGRLs are capable of promoting a differential migration of blood Gr1^{low} monocytes into the tissue that is likely to be mediated, at least in part, by CCL4.

DISCUSSION

In this study, we show that increased levels of TGRLs promote the migration of Gr1^{low} monocytes from the blood compartment into the surrounding tissue. The drop in the blood Gr1^{low} subset was accompanied by an increased crawling of these monocytes at the endothelial interface and a striking accumulation of CD68^{+} tissue macrophages in the heart, liver, and kidney. The changes in the monocyte subset distribution were not driven by systemic inflammation but were mediated, at least in part, by chemokines, mainly CCL4, via G-protein-coupled receptor(s).

While the cardiovascular disease (CVD) risks associated with increased levels of cholesterol-enriched lipoproteins, in particular low-density lipoprotein (LDL), are well known and have been extensively studied, the health-related consequences of elevated levels of TGRLs such as very low-density lipoprotein (VLDL) have been less well characterized and remain

(F) Percentage of BrdU incorporation into Gr1^{high} (left panel) and Gr1^{low} (right panel) monocytes over a period of 5 days following a single pulse of BrdU administered i.p. in three doses of 2 mg, 3 hr apart. Data are representative of two independent experiments. Results are expressed as mean ± SE, n = 3 in each group (unpaired t test); ns, nonsignificant.
See also Figure S3.

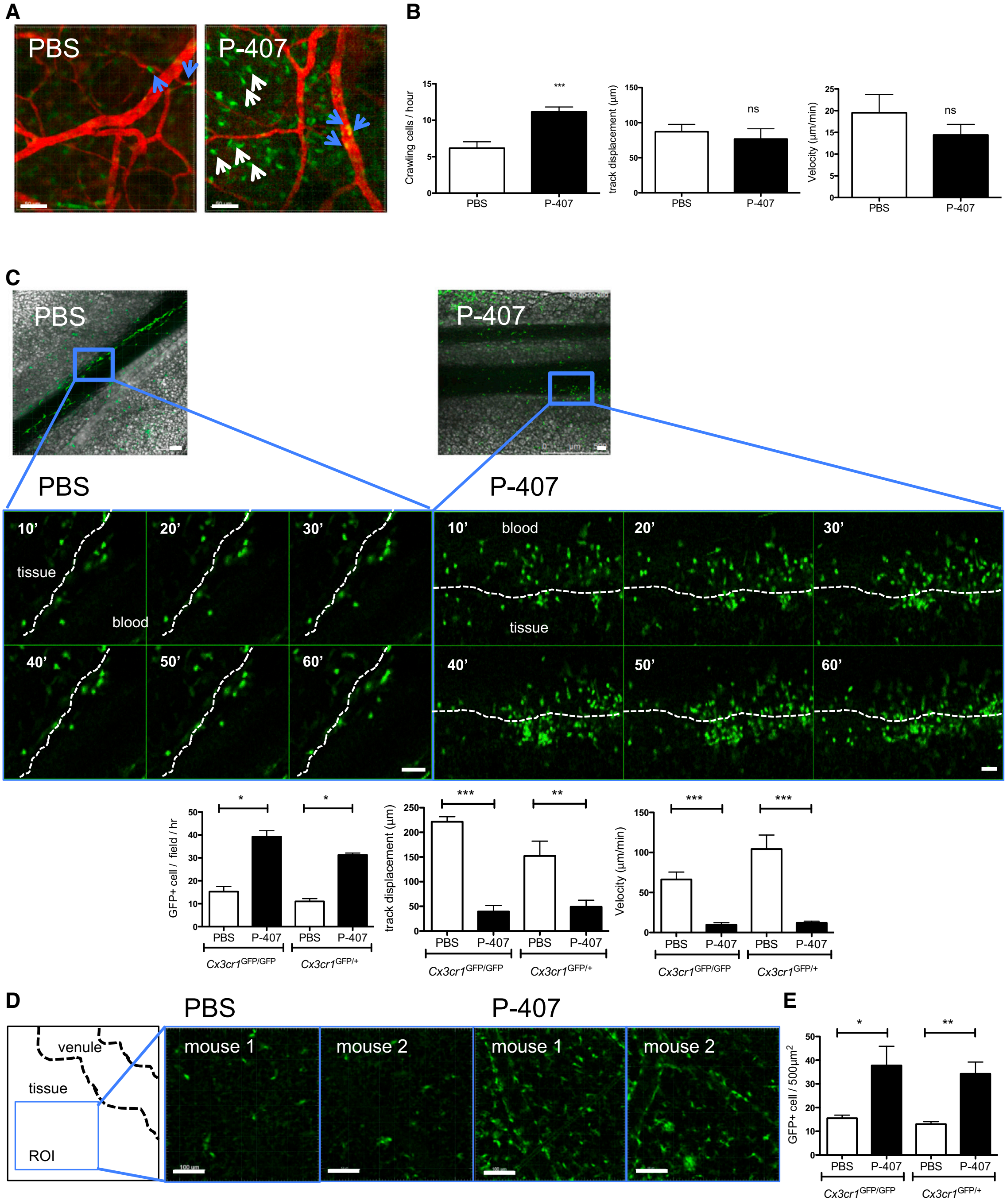

Figure 3. In Vivo Behavior of CX3CR1^{high} Monocytes under P-407-Induced Dyslipidemia

Cx3cr1$^{gfp/+}$ mice were treated with PBS or P-407 for 7 days and ear dermis vasculature imaged by intravital microscopy.

(A) Examples of intravascular crawling cells (blue arrow) or tissue cells (white arrow) with PBS or P-407 treatment. Scale bars, 50 μm.

(B) Quantitative representation of the number of intravascular crawling cells per hour, crawling velocity and track displacement after each treatment. n = 3 per group. ***$p < 0.001$ from PBS control. ns, nonsignificant. Scale bars, 50 μm.

(C) Same as (A) and (B) except that representative mesentery vasculature from *Cx3cr1*$^{gfp/gfp}$ or *Cx3cr1*$^{gfp/+}$ mice were imaged over 60 min. Region of interest (ROI) was selected which demonstrates gallery over time of CX3CR1^{high} cell accumulation at the endothelial interface (dotted line) following P-407 treatment. Representative of four mice; **$p < 0.01$. Scale bar, 50 μm.

(D and E) As in (C), where (D) shows examples of tissue from an ROI (500 μm^2) away from the venule showing accumulation of tissue GFP$^+$ cells after P-407 treatment. Two mice representative of four. Scale bar, 100 μm. (E) Quantitative representation of the number of tissue GFP$^+$ cells in PBS- and P-407-treated *Cx3cr1*$^{gfp/gfp}$ or *Cx3cr1*$^{gfp/+}$ mice. n = 3 per group. *$p < 0.05$ and **$p < 0.01$.

See also Figure S4.

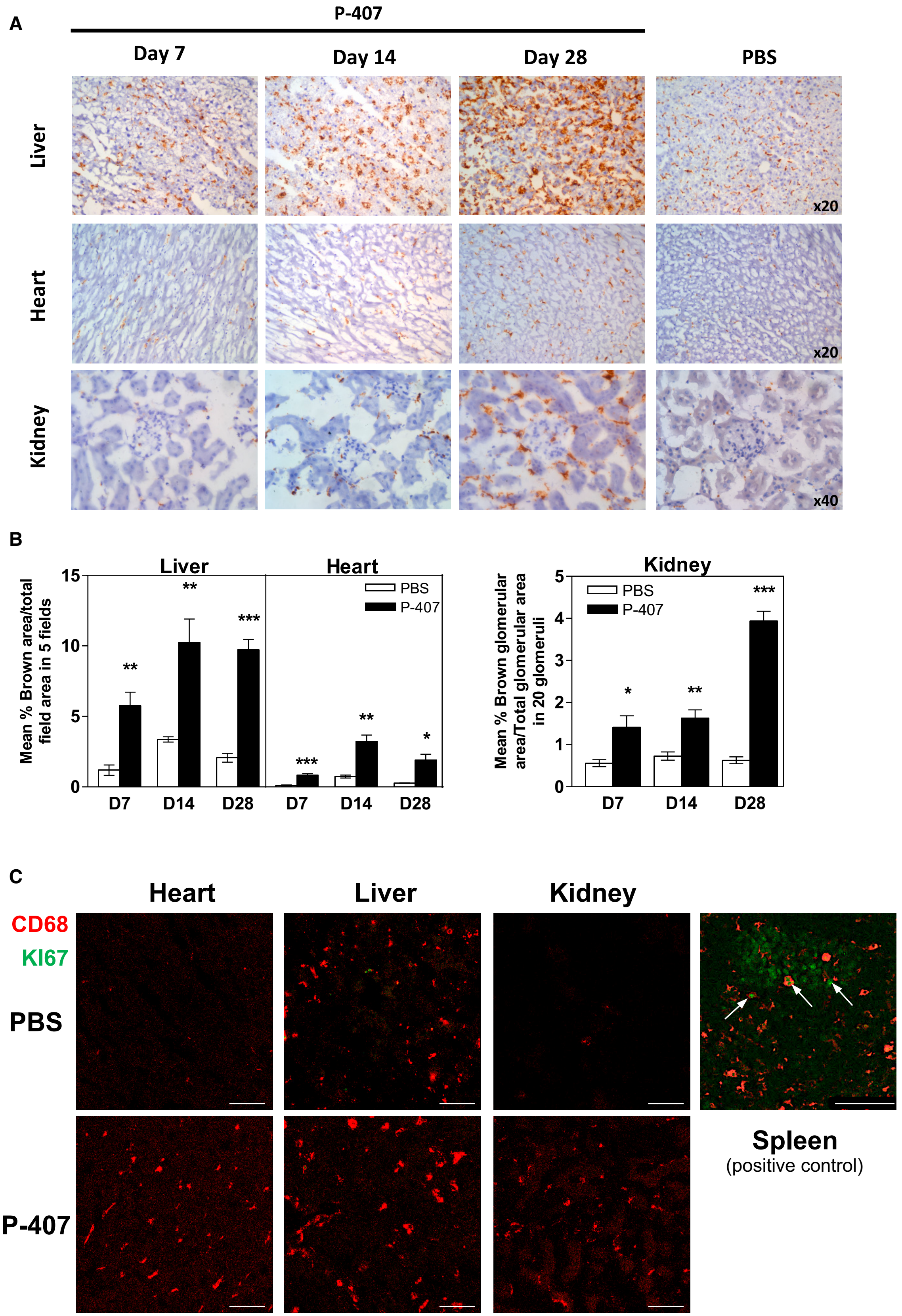

(legend on next page)

controversial (Sarwar et al., 2007). Increased TGRL levels are a key feature of the metabolic syndrome (Ninomiya et al., 2004) and may contribute to the associated morbidity. In addition, high TGRL levels are increasingly recognized as an independent risk factor for CVD. To investigate the impact of a hyper-TGRL environment, we used a murine model of P-407-induced hyper-TGRL (Johnston, 2004). We considered that the P-407 model was informative as (1) it induces a predominant increase in plasma TGRL, reaching levels similar to those detected in patients with familial hypertriglyceridemia or other genetic disorders of the triglyceride metabolism (Benlian et al., 1996; Pejic and Lee, 2006); (2) there is no overt inflammatory response, a confounding factor in other model of murine hyperlipidemia (Getz and Reardon, 2006); and (3) P-407 did not induce monocytosis or changes in monocyte surface phenotype, as previously reported in *Ldlr*$^{-/-}$ or *ApoE*$^{-/-}$ mice (Swirski et al., 2007; Tacke et al., 2007; Wu et al., 2009) and in human monocytes after a lipid-rich meal (Foster et al., 2013; Gower et al., 2011). Using the P-407 model, we demonstrated the migration of non-classical Gr1^{low} monocytes into surrounding tissues.

The relationship between blood monocytes and tissue macrophages remains enigmatic. There is evidence that, under steady-state conditions, monocytes are restricted to the blood compartment and adult tissue macrophages are embryonic in origin (Ginhoux and Jung, 2014). However, in certain tissue compartments, such as the skin and most notably the gut (Jaensson et al., 2008; Bain et al., 2014), there are experimental data indicating that circulating monocytes contribute significantly to the tissue macrophage compartment. These diverse findings suggest that blood monocyte migration to tissue compartments is influenced by environmental and cell-specific factors. The majority of the work has focused on the classical Gr1^{high} monocytes that can rapidly respond to inflammatory and bacterial signals. However, non-classical Gr1^{low} monocytes make up 40%–50% of the monocyte population in mice and ~10% in humans (Yona and Jung, 2010), and their functions are less well defined. There are reports that they can migrate in atherosclerotic settings (Nahrendorf et al., 2007) and can respond to viral and TLR7/8 cues (Carlin et al., 2013b; Cros et al., 2010), but it is their ability to patrol or survey the endothelial interface (Auffray et al., 2007; Carlin et al., 2013b; Cros et al., 2010) that has become widely recognized. Whether they can migrate and contribute to tissue macrophages under steady-state conditions remains unclear. Unexpectedly, our data indicated that the behavior of Gr1^{low} monocytes was markedly altered within a high-TGRL environment. We observed that in a high-TGRL environment, Gr1^{low} monocytes were depleted from the blood due to their retention at the endothelial interface. This was accompanied by a marked accumulation of CD68^{+} tissue macrophages in the heart, liver, and kidney. Adoptive transfer experiments of fluorescence-activated cell-sorted monocytes showed that, under our experimental conditions, Gr1^{low} monocytes were able to extravasate. This finding, together with the results of the Ki67 staining, ruled out the possibility that the increased number of tissue macrophages was due to in situ proliferation, as noted during inflammation (Davies et al., 2013).

We excluded the possibility that our findings were a direct effect of P-407. Consistent with the findings in patients with familial hypercholesterolemia (Mosig et al., 2009), we observed a similar drop in the Gr1^{low} monocytes in pre-atherosclerotic *Ldlr*$^{-/-}$ mice. Increased numbers of tissue macrophages have previously been described in high-fat-fed models (Fink et al., 2014; Lohmann et al., 2009). The production of monocytes is finely tuned by BM stem cell precursors, which can be influenced by dyslipidemia (Murphy et al., 2011). However, the P-407-induced increase in TGRLs did not cause obvious changes in the number of cMoPs or BM mature monocytes. Moreover, splenectomy did not alter the response of the Gr1^{low} monocytes to the hyper-TGRL environment, excluding splenic scavenging or extramedullary hematopoiesis as potential mechanisms. Collectively, our observations point toward a scenario where non-classical Gr1^{low} monocytes specifically respond to a rise in TGRL levels and, in the absence of overt inflammatory cues, extravasate into peripheral tissue and organs, contributing to the increased number of tissue CD68^{+} and F4/80^{+} macrophages. Our data, therefore, support the notion of a more dynamic role of Gr1^{low} monocytes in response to dyslipidemia rather than being just intravascular housekeepers or "blood macrophages" (Carlin et al., 2013b; Yona and Jung, 2010). Whether these cells extravasate in response to milder dyslipidemia or whether scavenging lipids in the tissue is part of their housekeeping functions requires further investigations.

Activation of Gr1^{low} monocytes and their human counterparts has been shown in murine lupus models and patients with SLE (Amano et al., 2005; Cros et al., 2010; Nakatani et al., 2010; Santiago-Raber et al., 2009; Yoshimoto et al., 2007), suggesting that Gr1^{low} monocytes might contribute to tissue injury (Misharin et al., 2014). In support of this, blockade of CX3CR1, which is highly expressed on Gr1^{low} monocytes, reduced monocyte recruitment to the kidney and the resulting inflammation (Inoue et al., 2005; Nakatani et al., 2010). Therefore, it is tempting to speculate that in conditions where there is abnormal lipid metabolism, such as chronic renal diseases, extravasated Gr1^{low}

Figure 4. Tissue Accumulation of CD68^{+} Cells under P-407 Treatment

(A) Representative photomicrographs of CD68 staining (brown) of PLP fixed liver, heart, and kidney sections after 7, 14, and 28 days of P-407 administration and after PBS treatment for 28 days. Magnification as indicated.

(B) Quantitative analysis of CD68^{+} cells in (A) specimens showing accumulation of CD68^{+} macrophages in the P-407-treated animals. For liver and heart, data are expressed as mean percentage ± SE of the brown-stained area in a selected field/total field area (five different fields per section). For the kidney, data represent mean percentage ± SE of the brown-stained glomerular area/total glomerular area for 20 glomeruli per section. Values represent the mean ± SE of at least four mice per group. p value by unpaired t test is shown (*p < 0.05, **p < 0.01, and ***p < 0.001).

(C) Representative images of the Ki-67 staining (green) and CD68 (red) of the sections (A) following 28 days of treatment with P-407 or PBS. Splenic sections were used as positive controls, and Ki-67^{+} cells are indicated with arrows. Scale bars, 75 μm.

See also Figure S4.

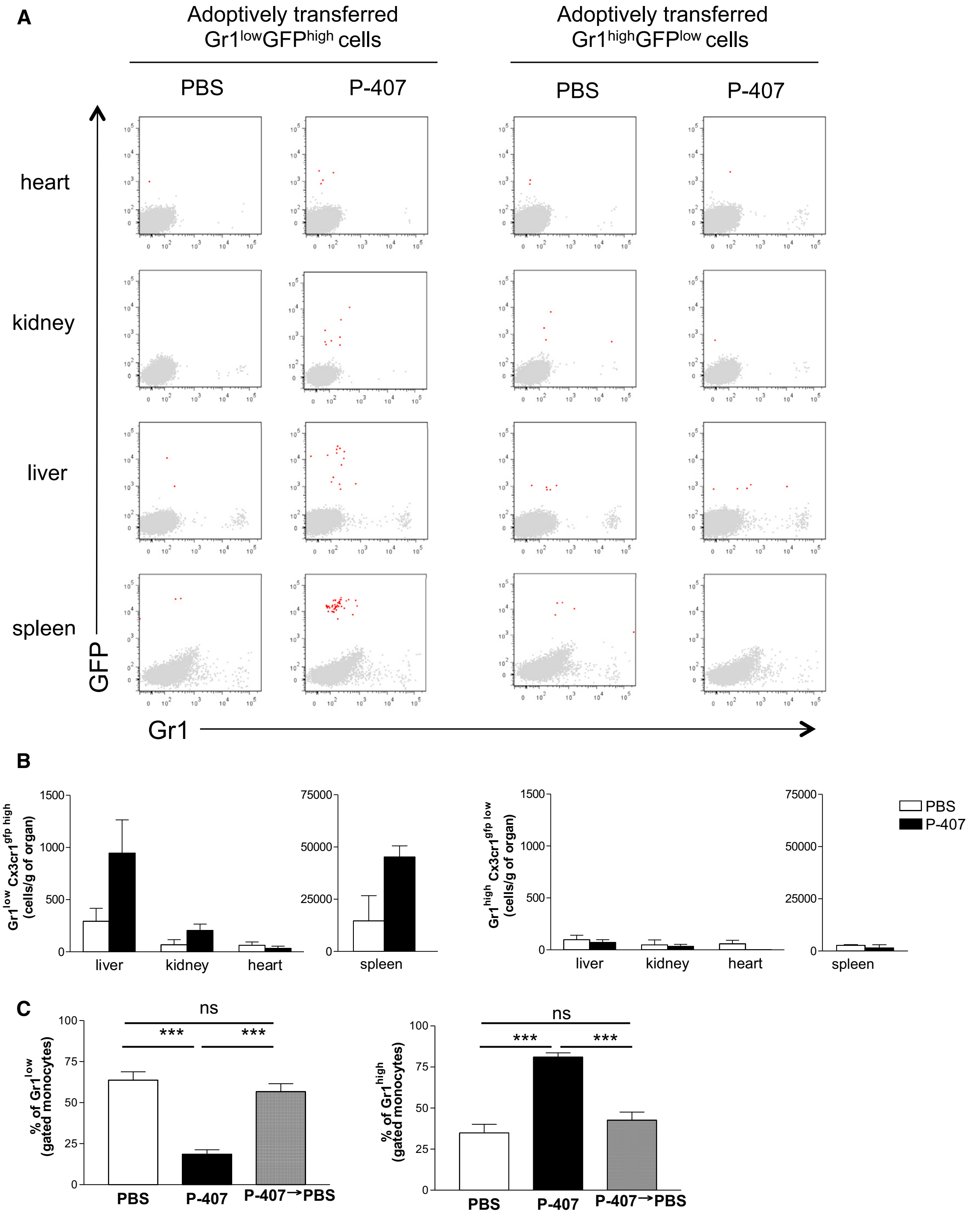

Figure 5. Tracing Monocyte Subset Migration by Adoptive Transfer

(A) $Gr1^{low}GFP^{high}$ or $Gr1^{high}GFP^{low}$ blood monocytes were sorted from CD45.2 *Cx3cr1*$^{gfp/gfp}$ mice. 0.1×10^6 $Gr1^{low}GFP^{high}$ or $Gr1^{high}GFP^{low}$ sorted monocytes were injected intravenously into CD45.1.B6 mice. At 16 hr, mice were injected with CD11b antibody to exclude blood contamination. Mice were perfused with PBS, and organs (liver, heart, kidney, and spleen) were analyzed for monocyte migration. Dot plot overlays display total cells (gray) and $CD45.2^{+}CD11b^{-}GFP^{+}$ cells (red).

(B) Quantitative representation of (A). Data are from two independent experiments (n = 4); values represent the mean ± SE of number of cells per gram of organ.

(C) B6 mice were treated with P-407 for 21 days or for 14 days followed by 7-day treatment with PBS. Mice treated with PBS for 21 days were used as controls. Data are expressed as percentage of $Gr1^{low}$ and $Gr1^{high}$ blood monocytes. Values represent mean ± SE, n = 4 mice per group. Significant p values are indicated (***$p < 0.001$; ns, nonsignificant; unpaired t test).

See also Figure S5.

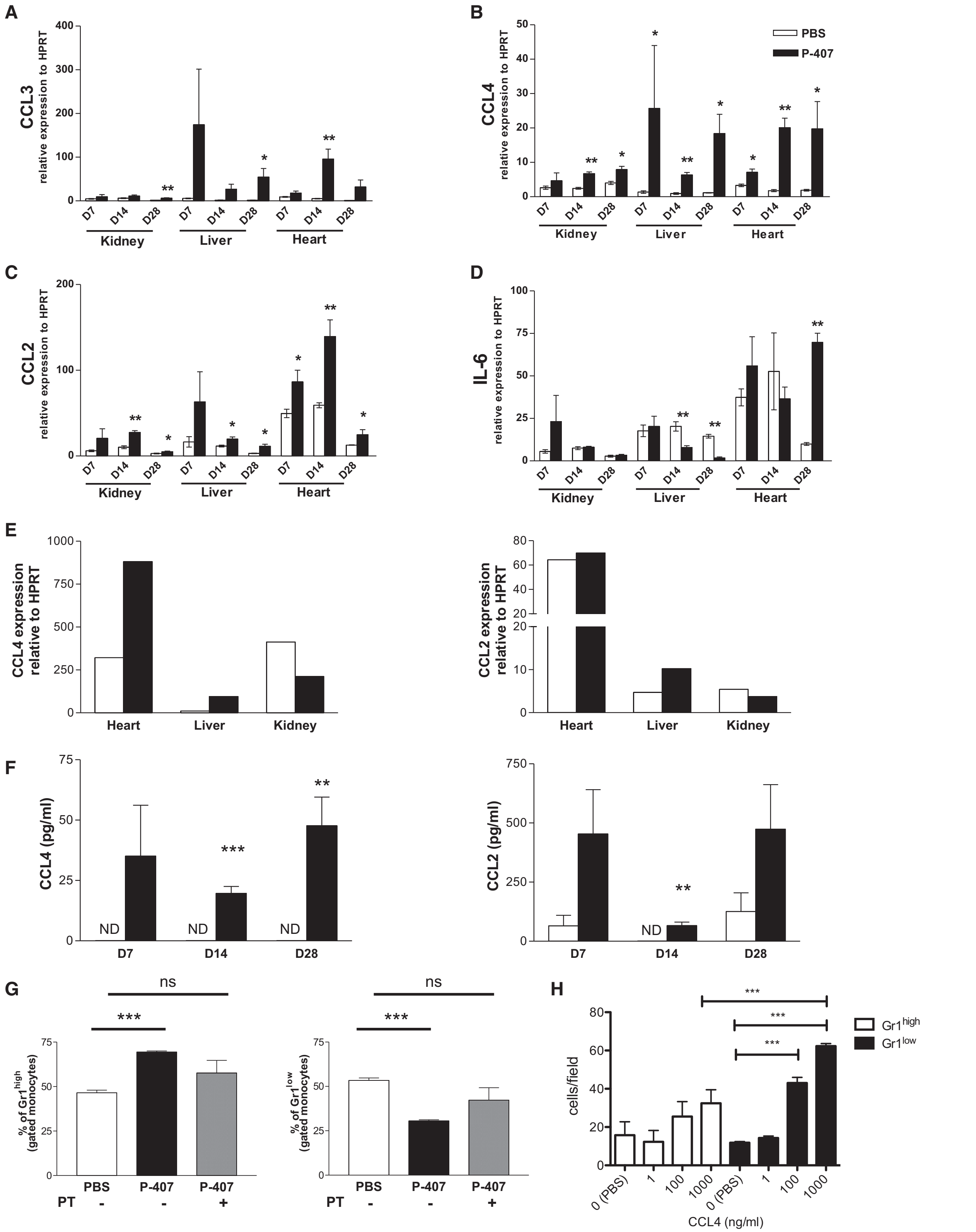

(legend on next page)

monocytes may convert into $CD68^+$/$F480^+$ macrophages and contribute to tissue damage. Monocyte-specific lineage reporters will be necessary to uncover the contribution of $Gr1^{low}$ monocytes to tissue homeostasis and pathology under hypertriglyceridemia.

We identified CCL4 as one of the potential molecules mediating the changes in the distribution of blood monocytes during hypertriglyceridemia. CCL4 is known to bind CCR5, a chemokine receptor expressed by both monocyte subpopulations (Tacke et al., 2007; Weber et al., 2000), a finding confirmed by us. TGRL levels did not alter the monocyte phenotype or modulate CCR5 expression, making it an unlikely candidate for the CCL4-mediated effect. However, administration of PT rectified some of the drop in blood $Gr1^{low}$ monocytes, confirming that a $G\alpha_i$ chemokine-receptor axis was involved. CCL4 can be produced by different cell types, including macrophages (Maurer and von Stebut, 2004). We found that CCL4 expression by macrophages from the heart and liver was enhanced in the hyper-TGRL environment. This suggests that a positive feedback loop may exist: foam cell formation, triggered by the hyperlipidemia, initiates $Gr1^{low}$ monocyte recruitment that in turn enhances foam cell formation, perpetuating the process. Considering that CCL4 has been shown to be expressed by macrophages in response to modified LDL (Wiesner et al., 2010) and that its receptor, CCR5, mediates $Gr1^{low}$ recruitment in atherosclerosis (Tacke et al., 2007), our data raise the possibility that this chemokine may play a key role in the increased CVD risk observed in chronic diseases associated with elevated TGRLs.

In conclusion, our study demonstrates that high TGRL levels alter monocyte subset distribution by promoting the extravasation of the non-classical $Gr1^{low}$ subset. These findings highlight the impact that an abnormal TGRL profile may have on the intravascular and extravascular behavior of blood monocytes.

EXPERIMENTAL PROCEDURES

Mice

C57BL/6, C57BL/6.CD45.1, C57BL/6.LDLR-deficient (*Ldlr*$^{-/-}$), and B6.129P-Cx3cr1tm1Litt/J (*Cx3cr1*$^{gfp/gfp}$) mice were used.

Poloxamer 407 Administration

Mice were injected i.p. with 10 mg Poloxamer 407 (Pluronic F-127, Sigma-Aldrich) solution or PBS every second day.

In Vitro Experiments

PBMCs from B6 mice were cultured overnight in the presence of P-407 concentration ranging from 50 μg/ml to 10 mg/ml and then stained for propidium iodide (PI) and Annexin V. PBMCs from B6 mice treated with PBS or P-407 for 28 days were stained for neutral lipid using the LipidTox kit (Invitrogen).

Cell Sorting and Adoptive Transfer Experiment

0.1×10^6 of fluorescence-activated cell-sorted $Gr1^{low}GFP^{high}$ or $Gr1^{high}$ GFP^{low} monocytes from CD45.2 *Cx3cr1*$^{gfp/gfp}$ were injected intravenously into congenic CD45.1 mice. Organs were collected 16 hr later.

PT

Mice treated for 10 days with P-407 were injected intravenously with 0.2 μg PT (Tocris Bioscience) or PBS. One day later, monocytes subsets were assessed by fluorescence-activated cell sorting analysis.

Splenectomy

Splenectomy was performed, and 4 weeks later, mice were treated with P-407 or PBS for 2 weeks. Monocyte subsets were then assessed by flow cytometry.

BrdU Pulsing

Three doses of BrdU (BD Pharmingen) were administered i.p. 3 hr apart. BrdU incorporation in blood monocytes was assessed by bleeding the mice 1, 3, and 5 days following BrdU injection.

Intravital Microscopy

Intravital microscopy was performed in mesentery and dermal ear microcirculation of *Cx3cr1*$^{gfp/+}$ and *Cx3cr1*$^{gfp/gfp}$ mice as previously described (Carlin et al., 2013a). See Supplemental Experimental Procedures for details.

Transwell Migration Assay

$Gr1^{low}$ and $Gr1^{high}$ blood monocytes were sorted using Aria II FACS (Becton-Dickson). 1×10^5 cells per well were seeded into 3-μm-pore transwell inserts (Corning), using a chemoattractant gradient of 1–1,000 ng/ml of recombinant mouse CCL4 or CCL2 (R&D Systems) or PBS and incubated at 37°C for 2 hr. Transwell inserts were fixed and slides were imaged using a 10× objective on an Olympus BX51 widefield fluorescence microscope and nuclei were manually counted from five fields.

Statistical Analysis

Comparisons between two groups were performed using two-tailed unpaired Student's t test or Mann-Whitney test as indicated in the figure legend. Statistically significant is defined as $p < 0.05$. N for each experiment is given in the figure or figure legends. *$p < 0.05$; **$p < 0.01$, ***$p < 0.001$.

SUPPLEMENTAL INFORMATION

Supplemental Information includes Supplemental Experimental Procedures, six figures, and four movies and can be found with this article online at http://dx.doi.org/10.1016/j.celrep.2015.08.020.

Figure 6. Chemokine Expression Triggered by P-407 Treatment

(A–D) The effect of P-407 on mRNA expression of chemokine/cytokine was analyzed by RT-PCR. Relative expression of (A) CCL3, (B) CCL4, (C) CCL2, and (D) IL-6 to the housekeeping gene HPRT. Kidney, liver, and heart specimens from mice treated with P-407 were compared with those from PBS-treated animals at each time point.

(E) CCL4 and CCL2 mRNA expression relative to HPRT in $CD45^+F4/80^+$ sorted tissue macrophages from liver, heart, and kidney after 14 days of P-407 or PBS treatment. Data are pooled from three animals per condition and representative of two independent experiments.

(F) Plasma concentrations of CCL4 and CCL2 after 7, 14, and 28 days of P-407 or PBS treatment. Values represent mean ± SE, n = 4 mice per group.

(G) Frequencies of $Gr1^{high}$ and $Gr1^{low}$ monocyte subsets in P-407-treated B6 mice injected with PT or PBS. PBS-treated mice were used as controls. Values represent mean ± SE, n = 3 mice per group. Results are representative of three independent experiments. Significant p values are indicated (*$p < 0.05$, **$p < 0.01$, and ***$p < 0.001$; unpaired t test). ND, nondetectable; ns, nonsignificant.

(H) Transwell migration assay. Fluorescence-activated cell-sorted $Gr1^{high}$ and $Gr1^{low}$ monocytes were added to a transwell chamber for 2 hr in the presence of recombinant mouse CCL4 (1–1,000 ng/ml) or PBS (0). The number of migrated cells per field was quantified (five fields per sample); pool of n = 3 mice in triplicate. Data are mean ± SE. ***$p < 0.001$ (unpaired t test).

See also Figure S6.

AUTHOR CONTRIBUTIONS

M.F.S. and L.B. conducted the experiments and analyzed the data; T.M., L.F.-J., M.R., and W.D.J. performed some experiments; M.C.P. assisted with data interpretation and edited the paper; H.T.C. analyzed the histology; and K.J.W and M.B. designed the experiments and wrote the paper.

ACKNOWLEDGMENTS

We thank the staff of the Central Biomedical Services at our institution for the care of the animals involved in this study. We are grateful to Lorraine Lawrence for the histological processing of the samples, Marta Szajna for her technical support, and Diane Scott for critical reading of the manuscript. This work was supported by the Wellcome Trust (grant number 088517). M.F.S. was supported by a studentship from King Saud University in Saudi Arabia and L.B. by a Swiss National Science Foundation fellowship.

Received: April 10, 2015
Revised: May 26, 2015
Accepted: August 6, 2015
Published: September 3, 2015

REFERENCES

Amano, H., Amano, E., Santiago-Raber, M.L., Moll, T., Martinez-Soria, E., Fossati-Jimack, L., Iwamoto, M., Rozzo, S.J., Kotzin, B.L., and Izui, S. (2005). Selective expansion of a monocyte subset expressing the CD11c dendritic cell marker in the Yaa model of systemic lupus erythematosus. Arthritis Rheum. *52*, 2790–2798.

Auffray, C., Fogg, D., Garfa, M., Elain, G., Join-Lambert, O., Kayal, S., Sarnacki, S., Cumano, A., Lauvau, G., and Geissmann, F. (2007). Monitoring of blood vessels and tissues by a population of monocytes with patrolling behavior. Science *317*, 666–670.

Bain, C.C., Bravo-Blas, A., Scott, C.L., Gomez Perdiguero, E., Geissmann, F., Henri, S., Malissen, B., Osborne, L.C., Artis, D., and Mowat, A.M. (2014). Constant replenishment from circulating monocytes maintains the macrophage pool in the intestine of adult mice. Nat. Immunol. *15*, 929–937.

Benlian, P., De Gennes, J.L., Foubert, L., Zhang, H., Gagné, S.E., and Hayden, M. (1996). Premature atherosclerosis in patients with familial chylomicronemia caused by mutations in the lipoprotein lipase gene. N. Engl. J. Med. *335*, 848–854.

Bruce, I.N. (2005). 'Not only…but also': factors that contribute to accelerated atherosclerosis and premature coronary heart disease in systemic lupus erythematosus. Rheumatology (Oxford) *44*, 1492–1502.

Carlin, L.M., Auffray, C., and Geissmann, F. (2013a). Measuring intravascular migration of mouse Ly6C(low) monocytes in vivo using intravascular microscopy. Curr. Protoc. Immunol. *Chapter 14*, 1–16, Unit 14.33.

Carlin, L.M., Stamatiades, E.G., Auffray, C., Hanna, R.N., Glover, L., Vizcay-Barrena, G., Hedrick, C.C., Cook, H.T., Diebold, S., and Geissmann, F. (2013b). Nr4a1-dependent Ly6C(low) monocytes monitor endothelial cells and orchestrate their disposal. Cell *153*, 362–375.

Cros, J., Cagnard, N., Woollard, K., Patey, N., Zhang, S.Y., Senechal, B., Puel, A., Biswas, S.K., Moshous, D., Picard, C., et al. (2010). Human CD14dim monocytes patrol and sense nucleic acids and viruses via TLR7 and TLR8 receptors. Immunity *33*, 375–386.

Davies, L.C., Rosas, M., Jenkins, S.J., Liao, C.T., Scurr, M.J., Brombacher, F., Fraser, D.J., Allen, J.E., Jones, S.A., and Taylor, P.R. (2013). Distinct bone marrow-derived and tissue-resident macrophage lineages proliferate at key stages during inflammation. Nat. Commun. *4*, 1886.

Epelman, S., Lavine, K.J., and Randolph, G.J. (2014). Origin and functions of tissue macrophages. Immunity *41*, 21–35.

Fink, L.N., Costford, S.R., Lee, Y.S., Jensen, T.E., Bilan, P.J., Oberbach, A., Blüher, M., Olefsky, J.M., Sams, A., and Klip, A. (2014). Pro-inflammatory macrophages increase in skeletal muscle of high fat-fed mice and correlate with metabolic risk markers in humans. Obesity (Silver Spring) *22*, 747–757.

Foster, G.A., Gower, R.M., Stanhope, K.L., Havel, P.J., Simon, S.I., and Armstrong, E.J. (2013). On-chip phenotypic analysis of inflammatory monocytes in atherogenesis and myocardial infarction. Proc. Natl. Acad. Sci. USA *110*, 13944–13949.

Geissmann, F., Jung, S., and Littman, D.R. (2003). Blood monocytes consist of two principal subsets with distinct migratory properties. Immunity *19*, 71–82.

Getts, D.R., Terry, R.L., Getts, M.T., Deffrasnes, C., Müller, M., van Vreden, C., Ashhurst, T.M., Chami, B., McCarthy, D., Wu, H., et al. (2014). Therapeutic inflammatory monocyte modulation using immune-modifying microparticles. Sci. Transl. Med. *6*, 219ra7.

Getz, G.S., and Reardon, C.A. (2006). Diet and murine atherosclerosis. Arterioscler. Thromb. Vasc. Biol. *26*, 242–249.

Ginhoux, F., and Jung, S. (2014). Monocytes and macrophages: developmental pathways and tissue homeostasis. Nat. Rev. Immunol. *14*, 392–404.

Gower, R.M., Wu, H., Foster, G.A., Devaraj, S., Jialal, I., Ballantyne, C.M., Knowlton, A.A., and Simon, S.I. (2011). CD11c/CD18 expression is upregulated on blood monocytes during hypertriglyceridemia and enhances adhesion to vascular cell adhesion molecule-1. Arterioscler. Thromb. Vasc. Biol. *31*, 160–166.

Hashimoto, D., Chow, A., Noizat, C., Teo, P., Beasley, M.B., Leboeuf, M., Becker, C.D., See, P., Price, J., Lucas, D., et al. (2013). Tissue-resident macrophages self-maintain locally throughout adult life with minimal contribution from circulating monocytes. Immunity *38*, 792–804.

Hettinger, J., Richards, D.M., Hansson, J., Barra, M.M., Joschko, A.C., Krijgsveld, J., and Feuerer, M. (2013). Origin of monocytes and macrophages in a committed progenitor. Nat. Immunol. *14*, 821–830.

Inoue, A., Hasegawa, H., Kohno, M., Ito, M.R., Terada, M., Imai, T., Yoshie, O., Nose, M., and Fujita, S. (2005). Antagonist of fractalkine (CX3CL1) delays the initiation and ameliorates the progression of lupus nephritis in MRL/lpr mice. Arthritis Rheum. *52*, 1522–1533.

Jaensson, E., Uronen-Hansson, H., Pabst, O., Eksteen, B., Tian, J., Coombes, J.L., Berg, P.L., Davidsson, T., Powrie, F., Johansson-Lindbom, B., and Agace, W.W. (2008). Small intestinal CD103+ dendritic cells display unique functional properties that are conserved between mice and humans. J. Exp. Med. *205*, 2139–2149.

Johnston, T.P. (2004). The P-407-induced murine model of dose-controlled hyperlipidemia and atherosclerosis: a review of findings to date. J. Cardiovasc. Pharmacol. *43*, 595–606.

Johnston, T.P., Beris, H., Zjumira, G., Kennedy, W., and Kennedy, J.L. (1993). Effects on splenic, hepatic, hematological, and growth parameters following high-dose poloxamer 407 administration to rats. Int. J. Pharm. *100*, 279–284.

Lohmann, C., Schäfer, N., von Lukowicz, T., Sokrates Stein, M.A., Borén, J., Rütti, S., Wahli, W., Donath, M.Y., Lüscher, T.F., and Matter, C.M. (2009). Atherosclerotic mice exhibit systemic inflammation in periadventitial and visceral adipose tissue, liver, and pancreatic islets. Atherosclerosis *207*, 360–367.

Ma, Y., Wang, W., Zhang, J., Lu, Y., Wu, W., Yan, H., and Wang, Y. (2012). Hyperlipidemia and atherosclerotic lesion development in Ldlr-deficient mice on a long-term high-fat diet. PLoS ONE *7*, e35835.

Maurer, M., and von Stebut, E. (2004). Macrophage inflammatory protein-1. Int. J. Biochem. Cell Biol. *36*, 1882–1886.

Misharin, A.V., Cuda, C.M., Saber, R., Turner, J.D., Gierut, A.K., Haines, G.K., 3rd, Berdnikovs, S., Filer, A., Clark, A.R., Buckley, C.D., et al. (2014). Nonclassical Ly6C(-) monocytes drive the development of inflammatory arthritis in mice. Cell Rep. *9*, 591–604.

Moore, K.J., and Tabas, I. (2011). Macrophages in the pathogenesis of atherosclerosis. Cell *145*, 341–355.

Mosig, S., Rennert, K., Krause, S., Kzhyshkowska, J., Neunübel, K., Heller, R., and Funke, H. (2009). Different functions of monocyte subsets in familial hypercholesterolemia: potential function of CD14+ CD16+ monocytes in detoxification of oxidized LDL. FASEB J. *23*, 866–874.

Murphy, A.J., Akhtari, M., Tolani, S., Pagler, T., Bijl, N., Kuo, C.L., Wang, M., Sanson, M., Abramowicz, S., Welch, C., et al. (2011). ApoE regulates

hematopoietic stem cell proliferation, monocytosis, and monocyte accumulation in atherosclerotic lesions in mice. J. Clin. Invest. *121*, 4138–4149.

Nahrendorf, M., Swirski, F.K., Aikawa, E., Stangenberg, L., Wurdinger, T., Figueiredo, J.L., Libby, P., Weissleder, R., and Pittet, M.J. (2007). The healing myocardium sequentially mobilizes two monocyte subsets with divergent and complementary functions. J. Exp. Med. *204*, 3037–3047.

Nakatani, K., Yoshimoto, S., Iwano, M., Asai, O., Samejima, K., Sakan, H., Terada, M., Hasegawa, H., Nose, M., and Saito, Y. (2010). Fractalkine expression and CD16+ monocyte accumulation in glomerular lesions: association with their severity and diversity in lupus models. Am. J. Physiol. Renal Physiol. *299*, F207–F216.

Ninomiya, J.K., L'Italien, G., Criqui, M.H., Whyte, J.L., Gamst, A., and Chen, R.S. (2004). Association of the metabolic syndrome with history of myocardial infarction and stroke in the Third National Health and Nutrition Examination Survey. Circulation *109*, 42–46.

Oh, J., and Hegele, R.A. (2007). HIV-associated dyslipidaemia: pathogenesis and treatment. Lancet Infect. Dis. *7*, 787–796.

Pejic, R.N., and Lee, D.T. (2006). Hypertriglyceridemia. J. Am. Board Fam. Med. *19*, 310–316.

Santiago-Raber, M.L., Amano, H., Amano, E., Baudino, L., Otani, M., Lin, Q., Nimmerjahn, F., Verbeek, J.S., Ravetch, J.V., Takasaki, Y., et al. (2009). Fcgamma receptor-dependent expansion of a hyperactive monocyte subset in lupus-prone mice. Arthritis Rheum. *60*, 2408–2417.

Sarwar, N., Danesh, J., Eiriksdottir, G., Sigurdsson, G., Wareham, N., Bingham, S., Boekholdt, S.M., Khaw, K.T., and Gudnason, V. (2007). Triglycerides and the risk of coronary heart disease: 10,158 incident cases among 262,525 participants in 29 Western prospective studies. Circulation *115*, 450–458.

Spann, N.J., and Glass, C.K. (2013). Sterols and oxysterols in immune cell function. Nat. Immunol. *14*, 893–900.

Swirski, F.K., Libby, P., Aikawa, E., Alcaide, P., Luscinskas, F.W., Weissleder, R., and Pittet, M.J. (2007). Ly-6Chi monocytes dominate hypercholesterolemia-associated monocytosis and give rise to macrophages in atheromata. J. Clin. Invest. *117*, 195–205.

Swirski, F.K., Nahrendorf, M., Etzrodt, M., Wildgruber, M., Cortez-Retamozo, V., Panizzi, P., Figueiredo, J.L., Kohler, R.H., Chudnovskiy, A., Waterman, P., et al. (2009). Identification of splenic reservoir monocytes and their deployment to inflammatory sites. Science *325*, 612–616.

Tacke, F., Ginhoux, F., Jakubzick, C., van Rooijen, N., Merad, M., and Randolph, G.J. (2006). Immature monocytes acquire antigens from other cells in the bone marrow and present them to T cells after maturing in the periphery. J. Exp. Med. *203*, 583–597.

Tacke, F., Alvarez, D., Kaplan, T.J., Jakubzick, C., Spanbroek, R., Llodra, J., Garin, A., Liu, J., Mack, M., van Rooijen, N., et al. (2007). Monocyte subsets differentially employ CCR2, CCR5, and CX3CR1 to accumulate within atherosclerotic plaques. J. Clin. Invest. *117*, 185–194.

Weber, C., Belge, K.U., von Hundelshausen, P., Draude, G., Steppich, B., Mack, M., Frankenberger, M., Weber, K.S., and Ziegler-Heitbrock, H.W. (2000). Differential chemokine receptor expression and function in human monocyte subpopulations. J. Leukoc. Biol. *67*, 699–704.

Wiesner, P., Choi, S.H., Almazan, F., Benner, C., Huang, W., Diehl, C.J., Gonen, A., Butler, S., Witztum, J.L., Glass, C.K., and Miller, Y.I. (2010). Low doses of lipopolysaccharide and minimally oxidized low-density lipoprotein cooperatively activate macrophages via nuclear factor kappa B and activator protein-1: possible mechanism for acceleration of atherosclerosis by subclinical endotoxemia. Circ. Res. *107*, 56–65.

Wu, H., Gower, R.M., Wang, H., Perrard, X.Y., Ma, R., Bullard, D.C., Burns, A.R., Paul, A., Smith, C.W., Simon, S.I., and Ballantyne, C.M. (2009). Functional role of CD11c+ monocytes in atherogenesis associated with hypercholesterolemia. Circulation *119*, 2708–2717.

Yona, S., and Jung, S. (2010). Monocytes: subsets, origins, fates and functions. Curr. Opin. Hematol. *17*, 53–59.

Yona, S., Kim, K.W., Wolf, Y., Mildner, A., Varol, D., Breker, M., Strauss-Ayali, D., Viukov, S., Guilliams, M., Misharin, A., et al. (2013). Fate mapping reveals origins and dynamics of monocytes and tissue macrophages under homeostasis. Immunity *38*, 79–91.

Yoshimoto, S., Nakatani, K., Iwano, M., Asai, O., Samejima, K., Sakan, H., Terada, M., Harada, K., Akai, Y., Shiiki, H., et al. (2007). Elevated levels of fractalkine expression and accumulation of CD16+ monocytes in glomeruli of active lupus nephritis. Am. J. Kidney Dis. *50*, 47–58.

Ziegler-Heitbrock, L. (2014). Reprint of: Monocyte subsets in man and other species. Cell. Immunol. *291*, 11–15.

Cell Reports

Supplemental Information

Triglyceride-Rich Lipoproteins Modulate the Distribution and Extravasation of Ly6C/Gr1^{low} Monocytes

Maha F. Saja, Lucie Baudino, William D. Jackson, H. Terence Cook, Talat H. Malik, Liliane Fossati-Jimack, Marieta Ruseva, Matthew C. Pickering, Kevin J. Woollard, and Marina Botto

Supplemental figure legends

Figure S1. P-407-induced dyslipidemia, Related to Figure 1. Total plasma TG (**A**) and CHOL (**B**) in B6 mice injected i.p. thrice weekly for 28 days with 10mg (equivalent to 0.5 g/kg) of P-407 or PBS while consuming a standard chow diet. TG and CHOL were always measured 24hrs after the P-407 injection and were significantly increased at each time point. Data are expressed as mean ±SE, n=4, unpaired t-test. Data representative of 2 independent experiments. *P<0.05, **P<0.01,*** P<0.001. (**C** and **D**) TG (**C**) and CHOL (**D**) levels following a single i.p. injection of P-407 (0.5 g/kg) or PBS. Lipid levels were measured at 0, 6, 24, 48, 72, and 96 hours post injection. Lipid concentrations peaked at 24hrs post P-407 injection and dropped sharply by 48hrs. Data are expressed as mean ±SE, n=3 in P-407 treated group. (**E** and **F**) Lipoprotein distribution of TG (**E**) and CHOL (**F**) in P-407-induced dyslipidemia assessed by chromatography on a Superose 6 HR 10/30 size-exclusion column. Pooled plasma (n=3 for each group) after 28 days. Very-low density lipoprotein (VLDL) corresponds to fractions 5 to 10, LDL to fractions 11-19 and high-density lipoprotein (HDL) to fractions 20-30. (**G**) Serum levels of apolipoprotein C-III (APOC3) and apolipoprotein E (APOE) after 7, 14 and 28 days of treatment with PBS or 0.5 g/kg P-407. Data are expressed as mean ±SE, n=4, unpaired t-test. *P<0.05, **P<0.01, ***P<0.001. (**H**) Circulating levels of IL-6, TNF-α and serum amyloid P component (SAP) at different time points during P-407 treatment. Results are expressed as mean ±SE, n=4 per time point, unpaired t-test. **P<0.01, ns = non significant. (**I** and **J**) Body (**I**) and spleen (**J**) weights at baseline and after 28 days of 0.5 g/kg P-407 treatment. Data are expressed as mean ±SE, n=6, unpaired t-test, **P<0.01. (**K**) Representative staining of liver (PAS), kidney, heart and spleen (H&E) after 28 days of P-407 injections. Arrows indicate foam cells in the P-407-treated group.

Figure S2. Peripheral blood cells in P-407-treated mice and blood monocyte distribution in high fat fed *Ldlr-/-* mice, Related to Figure 1 Quantitative analysis (numbers and percentages) of white blood cells (WBCs) (**A**), monocytes (**B**), T cells (**C** and **D**), B cells (**E** and **F**), PMNs (**G** and **H**) in mice injected with P-407 (closed circles) or PBS (open circles) over a period

of 28 days. Values represent mean ±SE, pooled data from 4 independent experiments, at least 7 mice per group at each time point, unpaired t-test. (**I**) Degree of survival of peripheral blood mononuclear cells over a range of P-407 concentrations (from 50 mg/ml to 10mg/ml). Frequency of Annexin V^+PI^+ monocytes (gated $CD11b^+CD115^+$ cells) at each P-407 concentration is represented. Representative data of 3 independent experiments. (**J** and **K**) Total plasma TG (**J**) and CHOL (**K**) in *Ldlr*-/- mice on high fat (HF) or low fat (LF) diets at day 0 and day 50. (**L**) Total number of blood monocytes in HF- or LF-fed *Ldlr*-/- mice after 50 days. (**M** and **N**) Quantitative analysis (numbers and percentages) of $Ly6C^{high}$ (equivalent to $Gr1^{high}$) and $Ly6C^{low}$ (equivalent to $Gr1^{low}$) monocytes in *Ldlr*-/- mice on a HF or LF diet for 50 days. (**J**-**N**) Data are expressed as mean ±SE, n=5, unpaired t-test. *P<0.05; **P<0.01, ***P<0.001.

Figure S3. Splenectomy and BrdU pulse experiment, Related to Figure 2. (**A**) Number of $Gr1^{high}$ and $Gr1^{low}$ monocytes after splenectomy in mice treated with PBS or P-407 for 14 days. Results are expressed as mean ±SE, n=3, *** P<0.0001, ns = non significant. (**B**) Representative dot plots showing BrdU incorporation into $Gr1^{high}$ and $Gr1^{low}$ blood monocytes over a period of 5 days following a single pulse of BrdU administered i.p. in three doses of 2mg, 3 hours apart. Percentages of BrdU incorporation in $Gr1^{low}$ monocytes (red rectangle) and $Gr1^{high}$ monocytes (blue rectangle) are indicated.

Figure S4. Behaviour of GFP^+ cells in extravascular tissue and quantitative analysis of F4/80^+ cells, Related to Figures 3-4. Migratory behaviour of GFP^+ cells in extravascular tissue of P-407- and PBS-treated C*x3cr1*$^{gfp/+}$ mice; **(A)** ear dermis: velocity, displacement and number of GFP+ cells; **(B)** mesentery tissue: velocity and displacement of GFP+ cells. **(C)** Representative photomicrographs and quantitative analysis of F4/80 staining (brown) of liver sections from PBS- and P-407-treated mice (14 days). Data expressed as mean percentage ±SE of brown-stained area in a selected field/total field area (5 fields per section), n=3. Data expressed as mean ±SE, n=4, unpaired t-test. * P<0.05; ***P<0.001, NS = non significant. **(D)** Bone marrow transplant. B6 mice were reconstituted with bone marrow cells from *Cx3cr1*$^{gfp/gfp}$ mice. Two months later, prior to any treatment, two mice were

culled and tissue macrophages analysed. Representative dot plots showing GFP^+ and $F4/80^+$ cells (gated $CD45^+$ cells).

Figure S5. Monocyte adoptive transfer and expression of surface markers on peripheral blood monocyte subsets following P-407 treatment, Related to Figure 5. (**A**) FACS-sorted $Gr1^{low}GFP^{high}$ ($0.1x10^6$) from CD45.2*Cx3cr1*$^{gfp/gfp}$ mice were injected i.v. into CD45.1B6 mice. 'P-407-recipient' represents PBS-exposed monocytes transferred into mice treated with P-407 for 14 days. 'P-407-donor' indicates monocytes isolated from P-407-treated mice (14 days) transferred to PBS-treated recipients. To exclude blood contamination an antibody against CD11b was injected prior to sacrificing the mice at 16hrs. Quantitative representation of number of cells in the liver per g of organ, n = 2-4, values represent the mean ±SE. **(B-F)** Expression of CD11b (**B**), CCR2 (**C**), CD68 (**D**), LFA1 (**E**) and CCR5 **(F)** on $Gr1^{high}$ and $Gr1^{low}$ monocytes in mice injected for 28 days with PBS or P-407. (**G**) CX3CR1 expression on $Gr1^{high}$ and $Gr1^{low}$ monocytes from *Cx3cr1*$^{gfp/+}$ mice. Values represent mean ±SE, unpaired t-test, n=3 per group, ns = non significant, *P<0.05; ***P<0.001. Data expressed as delta MFI (MFI=MFI-isotype control).

Figure S6. Chemokine expression triggered by P-407 treatment, Related to Figure 6. The effect of P-407 on mRNA expression of chemokine/cytokine was analysed by RT-PCR. Relative expression of (**A**) CX3CL1, (**B**) CXCL9, (**C**) CCL5, (**D**) CXCL1 to the house keeping gene HPRT. Kidney, liver, and heart specimens from mice treated with P-407 were compared with those from PBS-treated animals at each time point. Values represent mean ±SE, n= 4 mice per group, P values were all non significant, unpaired t-test. **(E).** FACS-sorted $Gr1^{high}$ and $Gr1^{low}$ monocytes ($1x10^5$) were added to 3µm transwells for 2hrs in the presence of recombinant mouse CCL2 (range 1-1000ng/ml) or PBS. The number of migrated cells/field was quantified (5 fields per sample), n=3 mice pooled in triplicate. Values represent mean ±SE, *P<0.05; *P<0.01 and ***P<0.001, unpaired t-test.

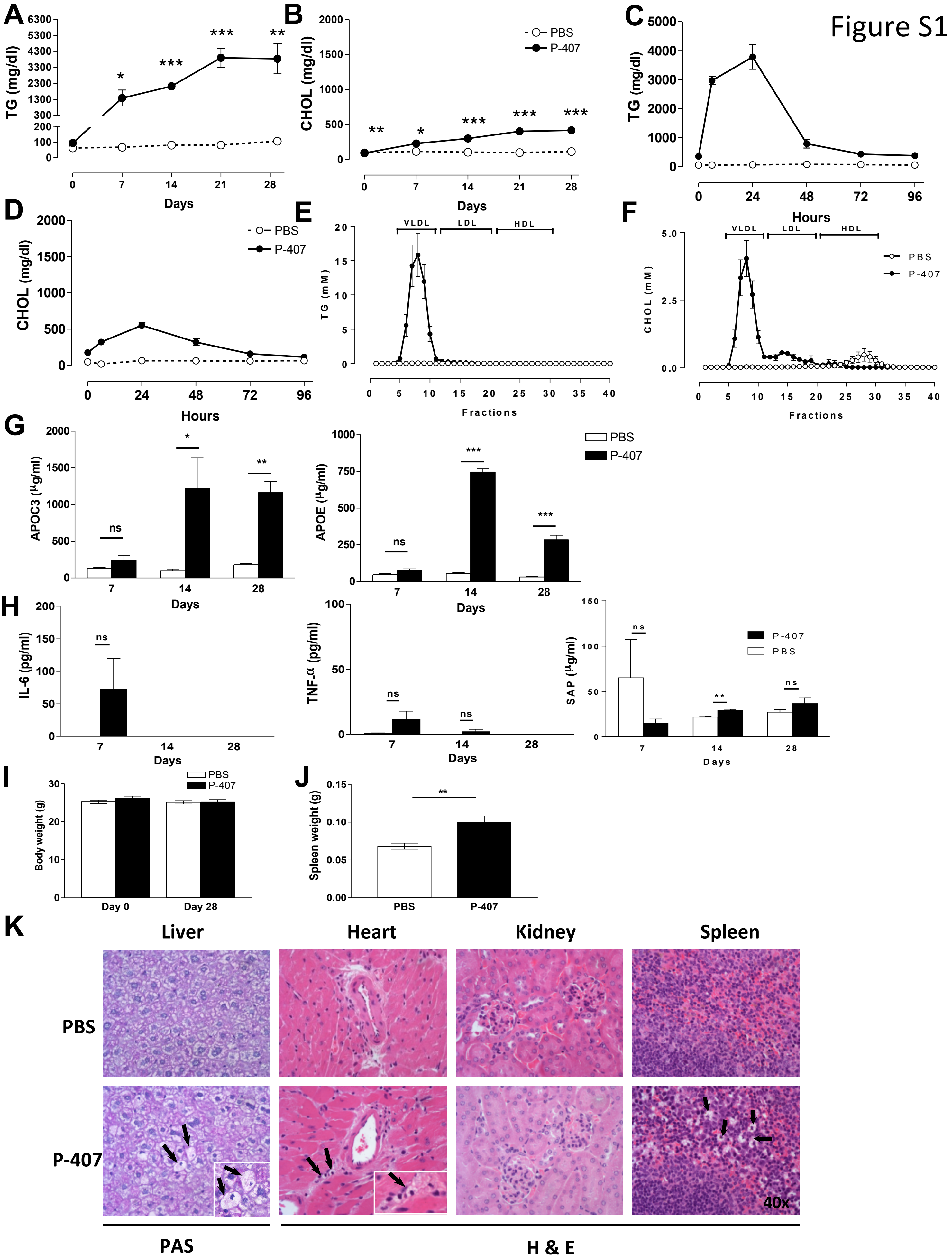
Figure S1
A
TG (mg/dl)
Days
B
CHOL (mg/dl)
PBS
P-407
Days
C
TG (mg/dl)
Hours
D
CHOL (mg/dl)
Hours
E
VLDL
LDL
HDL
TG (mM)
Fractions
F
CHOL (mM)
Fractions
G
APOC3 (µg/ml)
APOE (µg/ml)
Days
H
IL-6 (pg/ml)
TNF-α (pg/ml)
SAP (µg/ml)
Days
I
Body weight (g)
Day 0
Day 28
J
Spleen weight (g)
K
Liver
Heart
Kidney
Spleen
40x
PAS
H & E

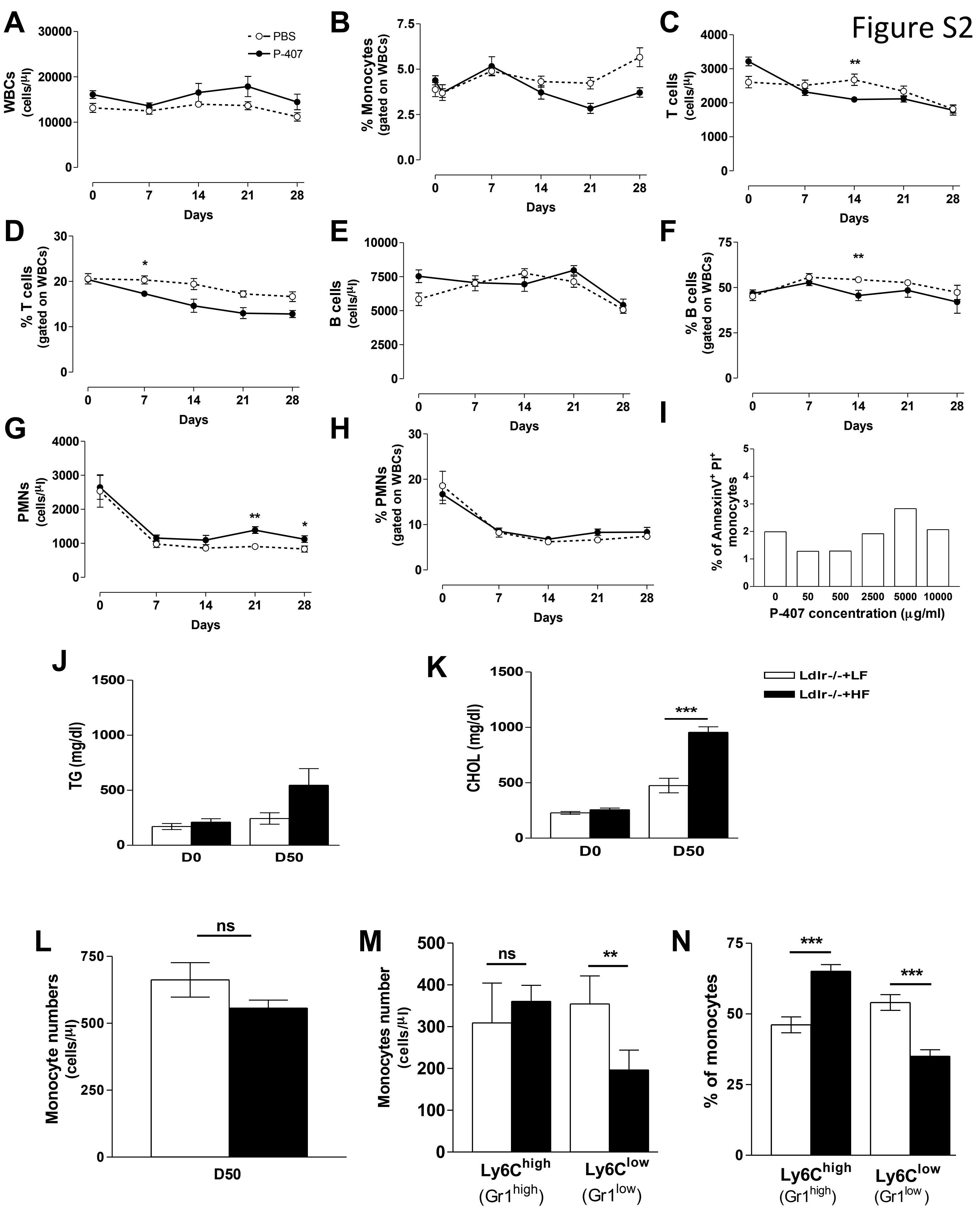
Figure S2
A
B
C
D
E
F
G
H
I
J
K
L
M
N
PBS
P-407
WBCs (cells/µl)
% Monocytes (gated on WBCs)
T cells (cells/µl)
% T cells (gated on WBCs)
B cells (cells/µl)
% B cells (gated on WBCs)
PMNs (cells/µl)
% PMNs (gated on WBCs)
% of AnnexinV+ PI+ monocytes
P-407 concentration (µg/ml)
Days
TG (mg/dl)
CHOL (mg/dl)
Ldlr-/-+LF
Ldlr-/-+HF
D0
D50
Monocyte numbers (cells/µl)
Monocytes number (cells/µl)
% of monocytes
Ly6Chigh (Gr1high)
Ly6Clow (Gr1low)
ns
**
***

Figure S3

A

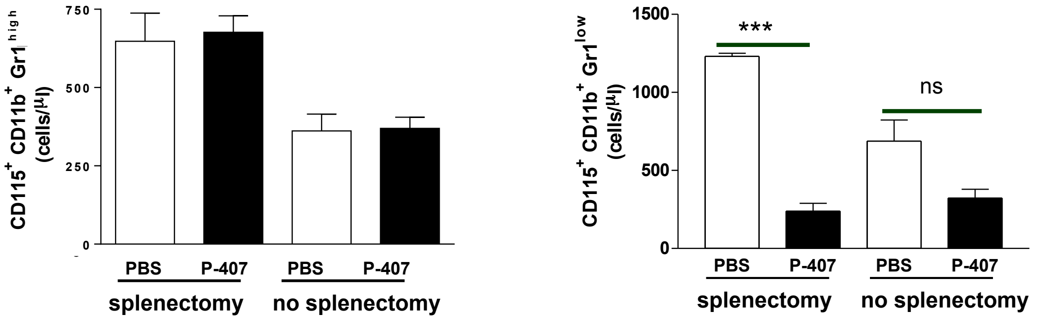

CD115+ CD11b+ Gr1 high (cells/µl)
750
500
250
0
PBS
P-407
PBS
P-407
splenectomy
no splenectomy
CD115+ CD11b+ Gr1 low (cells/µl)
1500
1000
500
0
***
ns

B

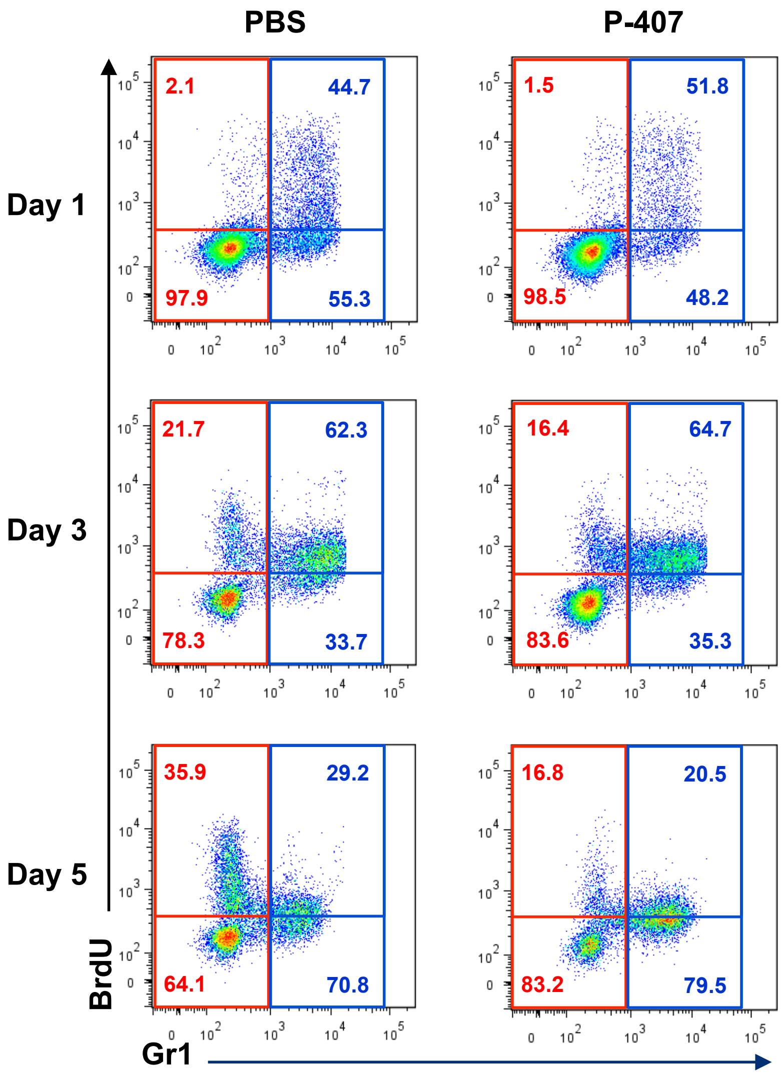

PBS
P-407
Day 1
Day 3
Day 5
2.1
44.7
97.9
55.3
1.5
51.8
98.5
48.2
21.7
62.3
78.3
33.7
16.4
64.7
83.6
35.3
35.9
29.2
64.1
70.8
16.8
20.5
83.2
79.5
BrdU
Gr1

Figure S4

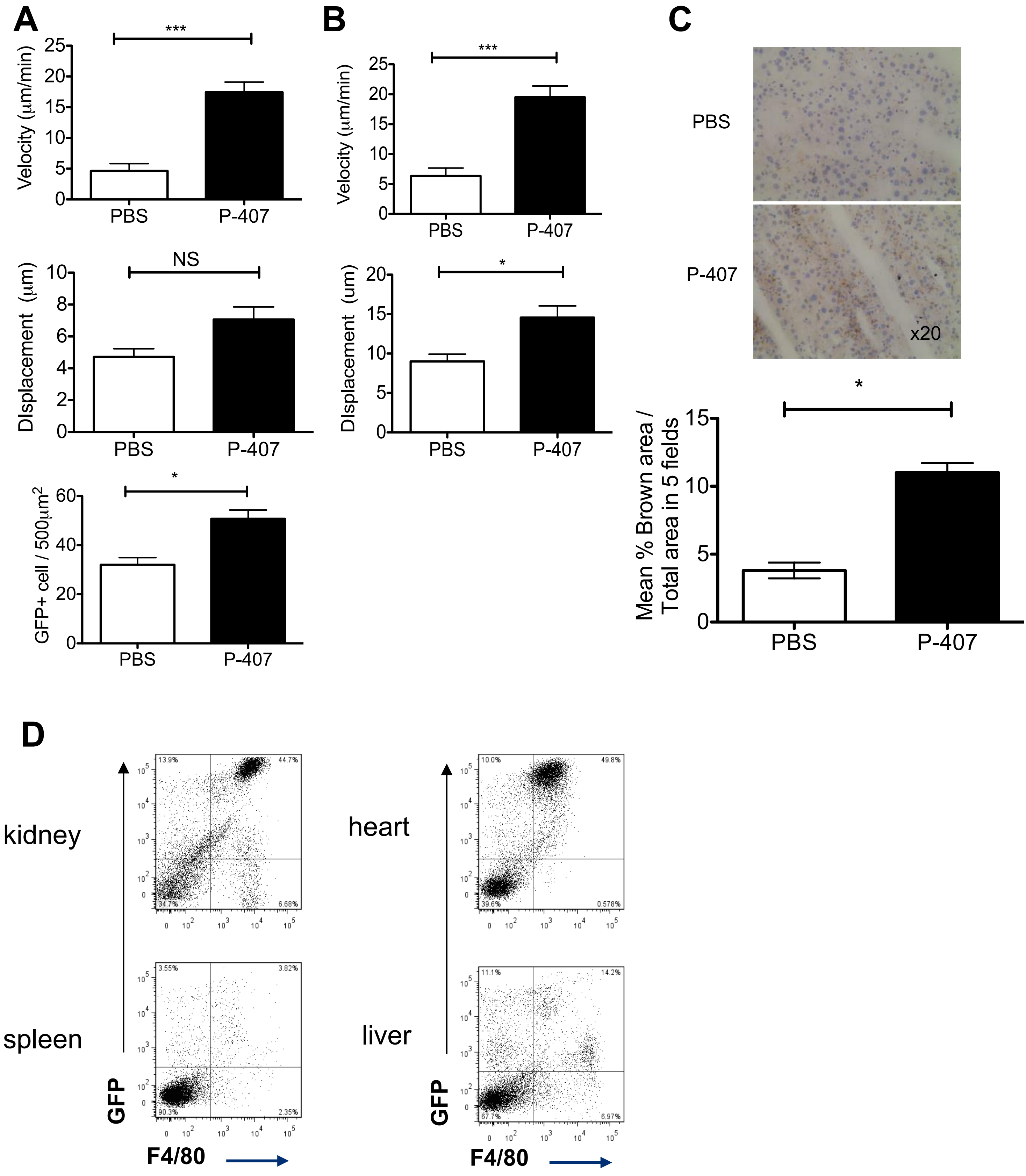
A
***
Velocity (μm/min)
PBS
P-407
NS
DIsplacement (μm)
*
GFP+ cell / 500μm2
B
***
Velocity (μm/min)
*
DIsplacement (um)
C
PBS
P-407
x20
Mean % Brown area / Total area in 5 fields
D
kidney
heart
spleen
liver
GFP
F4/80

Figure S5

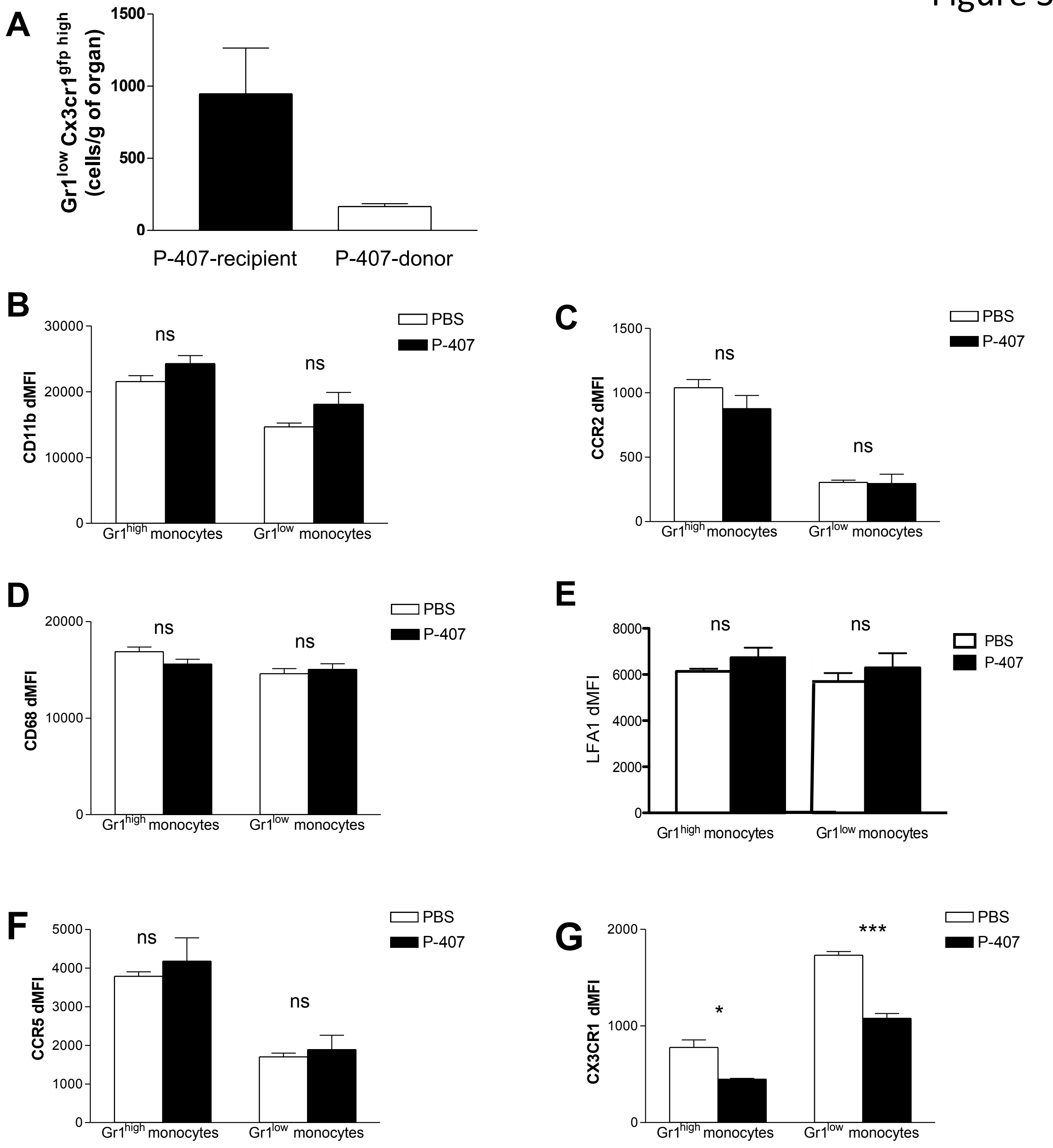
A
Gr1low Cx3cr1gfp high (cells/g of organ)
1500
1000
500
0
P-407-recipient
P-407-donor
B
CD11b dMFI
30000
20000
10000
0
ns
ns
PBS
P-407
Gr1high monocytes
Gr1low monocytes
C
CCR2 dMFI
1500
1000
500
0
ns
ns
PBS
P-407
Gr1high monocytes
Gr1low monocytes
D
CD68 dMFI
20000
10000
0
ns
ns
PBS
P-407
Gr1high monocytes
Gr1low monocytes
E
LFA1 dMFI
8000
6000
4000
2000
0
ns
ns
PBS
P-407
Gr1high monocytes
Gr1low monocytes
F
CCR5 dMFI
5000
4000
3000
2000
1000
0
ns
ns
PBS
P-407
Gr1high monocytes
Gr1low monocytes
G
CX3CR1 dMFI
2000
1000
0
*
***
PBS
P-407
Gr1high monocytes
Gr1low monocytes

Figure S6

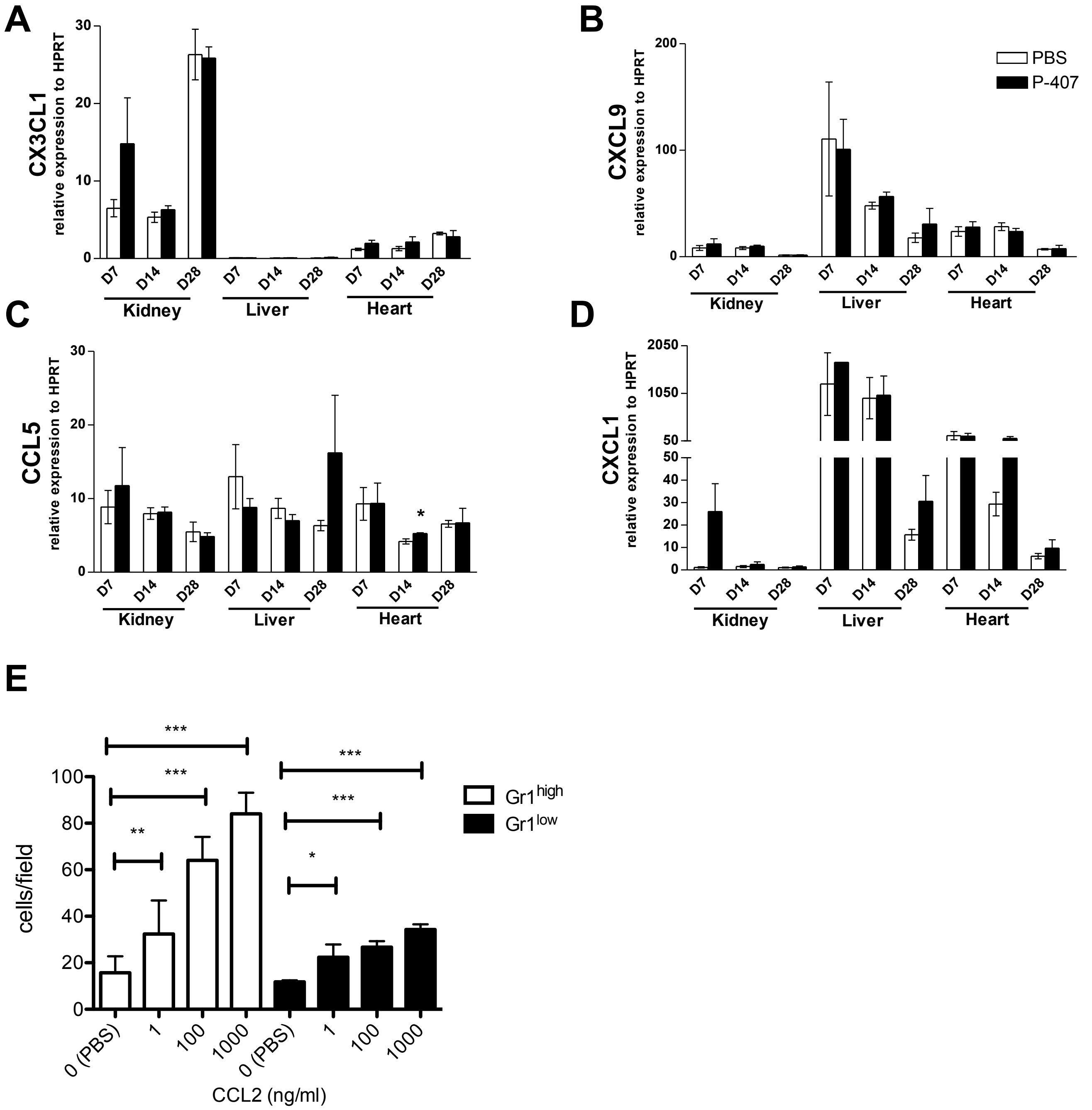

A
CX3CL1
relative expression to HPRT
B
CXCL9
relative expression to HPRT
PBS
P-407
C
CCL5
relative expression to HPRT
D
CXCL1
relative expression to HPRT
D7
D14
D28
Kidney
Liver
Heart
E
cells/field
Gr1high
Gr1low
0 (PBS)
1
100
1000
CCL2 (ng/ml)

Supplemental movies legends

Movie S1, related to Figure 3. Monocyte patrolling in control treated $CX3CR1^{GFP}$ ear dermis. Green is $CX3CR1^{high}$ (GFP+) monocyte/macrophage. Red = 70kDa Dextran. Scale bar = 50μm. Time = Hours:Mins:Secs.

Movie S2, related to Figure 3. Monocyte/macrophage in 7day P-407 (10mg) treated $CX3CR1^{GFP}$ ear dermis. Green is $CX3CR1^{high}$ (GFP+) monocyte/macrophage. Red = 70kDa Dextran. Scale bar = 50μm. Time = Hours:Mins:Secs.

Movie S3, related to Figure 3. Monocyte patrolling in control treated $CX3CR1^{GFP}$ mesentery venule. Green is $CX3CR1^{high}$ (GFP+) monocyte/macrophage. Scale bar = 50μm. Time = Hours:Mins:Secs.

Movie S4, related to Figure 3. Monocyte endothelial accumulation in 7day P-407 (10mg) treated $CX3CR1^{GFP}$ mesentery venule. Green is $CX3CR1^{high}$ (GFP+) monocyte/macrophage. Red = 70kDa Dextran. Scale bar = 50μm. Time = Hours:Mins:Secs.

Supplemental Experimental Procedures

Mice

C57BL/6 and C57BL/6.CD45.1 mice were purchased from Charles River. C57BL/6.LDLR-deficient mice (*Ldlr*$^{-/-}$) and B6.129P-*Cx3cr1*^{tm1Litt}/J (*Cx3cr1*$^{gfp/gfp}$) were purchased from Jackson Laboratory (Bar Harbor, USA). Mice of 8-12 weeks of age (weighing between 18-20g) were used for all experiments. All animals were housed in individually ventilated cages. All procedures were carried out according to the Institutional guidelines for the care and use of experimental animals and the ARRIVE guidelines. Animal studies were approved by the UK Home Office.

Poloxamer 407 administration and lipid levels

Poloxamer 407 (Pluronic F-127, Cat #P2443, Sigma-Aldrich, Dorset, UK) was dissolved overnight in cooled sterile PBS. Mice were injected i.p. with 200µl of P-407 solution (10mg, equivalent to 0.5 g/kg) or PBS every 2nd day. According to the experimental design blood samples were collected into heparin tubes at different time points prior to P-407 injection and 24hrs following drug administration. CHOL and TG levels were measured by a colorimetric assay using the Cholesterol and Triglyceride infinity Reagent (TR13421 & TR22421, respectively, Thermo-Scientific, Middletown, USA). CHOL and TG distribution in plasma lipoprotein fractions was determined on pooled sera by fast performance liquid chromatography gel filtration on a Superose 6 HR 10/30 size-exclusion column. Levels of plasma Apo-E and APOC3 were measured by ELISA (Cusabio, Hubei Province, P.R. China).

In vitro experiments

PBMC from B6 mice were cultured overnight in the presence of P-407 concentration ranging from 50ug/ml to 10mg/ml and then stained for propidium iodide (PI) and Annexin V. PBMC of B6 mice treated with PBS or P-407 for 28 days were stained for neutral lipid using the LipidTox kit (Invitrogen, Paisley, UK).

Bone marrow transplant

Eight week-old female mice were irradiated at 8 Gy using a 137Cs c-ray source and reconstituted with 10^7 bone marrow cells from *Cx3cr1*$^{gfp/gfp}$ mice. Two months later 3 mice were culled and organs (liver, heart, kidney and spleen) were digested at 37^C for 30 min in PBS containing 3% FCS, 1mg/ml collagenase D (Roche), 100U/ml DNaseI (Roche) and 2.4mg/ml Dispase. Cells were analysed by flow cytometry.

Flow cytometry

Peripheral blood was collected from the tail vein or by cardiac puncture under terminal anaesthesia in 5% EDTA tubes and immediately placed on ice. For bone marrow staining, femurs and tibia were harvested and bone marrow flushed. Red cells were lysed using FACS lysing buffer (BD) in accordance to manufacturer's instructions. The following antibodies were used: anti-CD115 (AFS98), anti-Gr1 (RB6-8C5), anti-CD11b (M1-70), anti-CCR5 (HM-CCR5), anti-Ly6C (HK1.4), anti-F4/80 (BM8), anti-CD117 (2B8), anti-LFA1 (M17/4) (all from eBiosciences); anti-CD19 (1D3), anti-CD45.2 (104), anti-CD62L (MEL-14) (all from BD Pharmingen); anti-CCR2 (475301) (R&D systems); anti-CD68 (FA11) and anti-thy1.2 (53-2.1) (Biolegend). Staining was

performed in the presence of a saturating concentration of 2.4G2 monoclonal antibody (anti-CD16/32). In the blood, monocyte subsets were identified as $CD11b^{+}CD115^{+}$ and $Gr1^{high}$ or $CD11b^{+}CD115^{+}$ and $Gr1^{low}$. In the bone marrow cMoPs were identified as $CD117^{+}CD115^{+}Ly6C^{+}CD11b^{-}$ cells, monocytes as $CD117\text{-}CD115^{+}CD11b^{+}$. Quantification of cell numbers in blood was performed by using AccuCheck counting beads (PCB100, Invitrogen). Flow cytometry was performed with a BD FACSVerse (BD Biosciences, CA, USA). Data were analysed using FlowJo software, version 7.6.5 (TreeStar Inc, Ashland, OR, USA).

Histology and immunohistochemistry

For staining, tissues were fixed in PLP (Periodate-Lysine-Paraformaldehyde) followed by 7% sucrose. Acetone fixed-5μm thick sections were then blocked with 10% milk and 0.03% hydrogen peroxide, sequentially. The sections were stained with anti-CD68 (FA-11, AbD serotec, MCA 1957) and developed using the Polink-2 plus HRP detection kit (GBI Labs, # D46-18). Analysis was performed using a Zeiss microscope. Quantification was carried as previously described (Bhatia et al., 2007). Paraffin sections were stained with H&E or Period Acid-Schiff (PAS) as indicated in the figure legend. For Ki-67 staining, cryostat sections of snap frozen tissue were fixed with acetone and blocked with 20% normal rabbit serum. Sections were sequentially stained with rat mAb against CD68 (Alexa Fluor® 488, FA-11, Biolegend), F4/80 (Biolegend, BM8) and Ki67 (eFluor® 570, SolA15, eBioscience). Coverslips mounted using Vectashield with DAPI (Vector Laboratories). Confocal fluorescence images were acquired on a Leica SP5 confocal microscope with a 40 or 63 x oil immersion objective.

Cell sorting and adoptive transfer experiment

$Gr1^{low}GFP^{high}$ or $Gr1^{high}GFP^{low}$ blood monocytes were sorted from CD45.2*Cx3cr1*$^{gfp/gfp}$ mice using Aria II FACS (Becton-Dickson). $0.1x10^6$ $Gr1^{low}GFP^{high}$ or $Gr1^{high}GFP^{low}$ sorted monocytes (>95% purity) were injected i.v. into congenic CD45.1 mice. The same protocol was used to isolated $Gr1^{low}GFP^{high}$ monocytes from CD45.2*Cx3cr1*$^{gfp/gfp}$ mice treated with P-407 for 14 days. At 16hrs mice were injected with 3μg of antibody against CD11b to exclude blood contamination. Mice were perfused with PBS and organs (liver, heart, kidney and spleen) were digested in PBS containing 3% FCS, 1mg/ml collagenase D (Roche), 100U/ml DNaseI (Roche) and 2.4mg/ml Dispase at 37^C for 30 min. The digested cell suspension was then passed through 70um cells strainers. Tissue macrophages from PBS or P-407 treated animals were identified using antibodies against CD45 and F4/80 and sorted using Aria II FACS. Pre-injection of 3μg of antibody against CD115 was used to exclude blood contamination in tissue preparation.

qPCR

RNA was purified using TRIzol reagent (Invitrogen, Paisley, UK) and treated with DNase I (Ambion). RNA was quantified by absorbance spectroscopy (Nanodrop) and cDNA synthesized by reverse-transcriptase reaction (Bio-rad). qRNA analysis was performed using the Real-time PCR Detection System and Brillant II SYBR Green (Agilent technology). mRNA was quantified using a standard curve generated with serial dilutions of input cDNA and normalized using HPRT mRNA concentration. The data are

expressed as relative expression to the HPRT housekeeping gene. Primers are listed below.

	forward primer	reverse primer
CCL2	AGGTGTCCCAAAGAAGCTGTA	ATGTCTGGACCCATTCCTTCT
CCL3	TGCCCTTGCTGTTCTTCTCT	CCCAGGTCTCTTTGGAGTCA
CCL4	GCCCTCTCTCTCCTCTTGCT	GTCTGCCTCTTTTGGTCAGG
CCL5	CCCTCACCATCATCCTCACT	CCCTCACCATCATCCTCACT
CX3CL1	ATTTGTGTACTCTGCTGCC	TCTCCAGGACAATGGCAC
CXCL9	TTTTCCTCTTGGGCATCATC	AGTCCGGATCTAGGCAGGTT
IL-6	TGATGGATGCTACCAAACTGG	TTCATGTACTCCAGGTAGCTATGG
HPRT1	TCAGTCAACGGGGGACATAAA	GGGGCTGTACTGCTTAACCAG

Cytokine levels

Plasma was collected on day 1, day 7 , day 14 and day 28 from mice treated with P-407 or PBS. IL-8, CCL2, IL-6 and TNF-α were measured using a bead multiplex assay (eBioscience) according to the manufacturer's instructions. Bead fluorescence emission was detected using FACSVerse (BD Biosciences) and data analysed using Flow Cytomix (eBiosciences). CCL4 and serum amyloid P componenet (SAP) were measured by ELISA (R&D system and Genway, respectively).

Pertussis Toxin

Mice treated for 10 days with P-407 were injected intravenously with 0.2ug of pertussis toxin (Tocris bioscience) or PBS. One day later monocytes subsets were assessed by FACS analysis.

Splenectomy

Splenectomy was performed and 4 weeks later mice were treated with P-407 or PBS for 2 weeks. Monocytes subsets were then assessed by flow cytometry.

BrdU pulsing

BrdU pulsing was done as described by Yona et al (Yona et al., 2013). Three doses of 2mg BrdU (5-bromo-2-deoxyuridine, BD Pharmingen) were administered i.p. 3 hrs apart. BrdU incorporation in blood monocytes was assessed by bleeding the mice 1, 3, and 5 days following BrdU injection. Monocytes were then stained for BrdU according to manufacturer's instructions (APC BrdU Flow Kit, BD Pharmingen).

Intravital microscopy and image analyses of Ear and Mesentery

Intravital microscopy was performed in mesentery and dermal ear microcirculation of *Cx3cr1*$^{gfp/+}$ and *Cx3cr1* $^{gfp/gfp}$ mice, as previously described (Carlin et al., 2013a). Mice were treated with PBS or P-407 (10mg) for 7 days and under anesthesia mesentery exposed by minimal surgical intervention or ear placed on custom microscopy plate for imaging. Animals were kept warmed and tissue superinfused with warmed fresh saline. For dermal ear imaging, mice were injected with 70kDa Tetramethylrhodamine-dextran (Molecular Probes) to label vasculature. Over 1hr, tissue vasculature was

imaged using Leica SP5 confocal microscope using 20x objective. Imaging exposure was kept minimal to prevent phototoxicity. Areas of interest were analysed using Imaris (Bitplane, version 7.7) and numbers of cells analysed within defined region of interest (ROI). Tracking of cell motility in four dimensions was performed using Imaris. Moving cells were automatically and manually tracked. Track displacement (distance between first and last cell's position) and velocity were calculated from these values.

Transwell migration assay

$Gr1^{low}$ and $Gr1^{high}$ blood monocytes were sorted using Aria II FACS (Becton-Dickson). 1 x 10^5 cells per well were seeded into 3µm-pore transwell inserts (Corning), using a chemoattractant gradient of 1-1000 ng/ml of recombinant mouse CCL4 or CCL2 (R&D systems) or PBS and incubated at 37°C for 2 hours. Non-adherent cells were removed and the transwell inserts were fixed in 4% paraformaldehyde (PFA). Non-migrated cells were removed using a cotton bud before the membranes were excised and mounted on microscope slides in Vectashield mounting media containing DAPI (Vector Labs). Slides were imaged using a 10X objective on an Olympus BX51 widefield fluorescence microscope and nuclei were manually counted from 5 fields.

Statistics

Comparisons between two groups were performed using two-tailed unpaired Student's *t*-test or Mann-Whitney test as indicated in the figure legend. Statistically significant is defined as $P < 0.05$. N for each experiment is given in the figure or figure legends. *P<0.05; **P<0.01, ***P<0.001

Supplemental References

Bhatia, V.K., Yun, S., Leung, V., Grimsditch, D.C., Benson, G.M., Botto, M.B., Boyle, J.J., and Haskard, D.O. (2007). Complement C1q reduces early atherosclerosis in low-density lipoprotein receptor-deficient mice. Am J Pathol *170*, 416-426.

Yona, S., Kim, K.W., Wolf, Y., Mildner, A., Varol, D., Breker, M., Strauss-Ayali, D., Viukov, S., Guilliams, M., Misharin, A., *et al.* (2013). Fate mapping reveals origins and dynamics of monocytes and tissue macrophages under homeostasis. Immunity *38*, 79-91.
